# Supplementary material for: Nanog Fluctuations in Embryonic Stem Cells Highlight the Problem of Measurement in Cell Biology
Source: Biophys J. 2017 Jun 20;112(12):2641–52. doi: 10.1016/j.bpj.2017.05.005 (PMC5479053; doi:10.1016/j.bpj.2017.05.005)
Supplement: Document S1. Supporting Materials and Methods, Figs. S1–S4, and Tables S1 and S2 [file mmc1.pdf]

Biophysical Journal, Volume *112*

Supplemental Information

Nanog Fluctuations in Embryonic Stem Cells Highlight the Problem of Measurement in Cell Biology

Rosanna C.G. Smith, Patrick S. Stumpf, Sonya J. Ridden, Aaron Sim, Sarah Filippi, Heather A. Harrington, and Ben D. MacArthur

Nanog Fluctuations in Embryonic Stem Cells Highlight the Problem of Measurement in Cell Biology

Supporting Material

Rosanna C G Smith[1], Patrick S Stumpf[1], Sonya J Ridden[2], Aaron Sim[3], Sarah Filippi[4], Heather A Harrington[5], and Ben D MacArthur[1,2,6,*]

[1]Centre for Human Development, Stem Cells and Regeneration, Faculty of Medicine, University of Southampton, SO17 1BJ, UK.
[2]Mathematical Sciences, University of Southampton, SO17 1BJ, UK.
[3]Department of Life Sciences, Imperial College London, SW7 2AZ, UK.
[4]Department of Mathematics, Department of Epidemiology and Biostatistics, Imperial College London, SW7 2AZ, UK.
[5]Mathematical Institute, University of Oxford, OX2 6GG, UK.
[6]Institute for Life Sciences, University of Southampton, SO17 1BJ, UK.
[*]To whom correspondence should be addressed: bdm@soton.ac.uk

Contents

1 Mathematical details **1**
1.1 Allelic synchronization and mRNA co-expression dynamics . . . 1
1.1.1 Two single-state alleles with upstream regulation . . . 1
1.1.2 One two-state allele with upstream regulation . . . 3
1.1.3 Two two-state alleles with upstream regulation . . . 5
1.2 Bifurcation curves for Nanog dynamics . . . 7
1.3 Reporter perturbations . . . 8
1.4 Single allele reporter strategies . . . 8
1.4.1 Knock-in reporters . . . 8
1.4.2 Pre/post (PP) reporters . . . 9
1.4.3 Multiple pre/post (MPP) reporters . . . 9
1.4.4 Fusion reporters . . . 10
1.4.5 BAC reporters . . . 10
1.5 Dual allele reporter strategies . . . 10
1.6 Decay constant mismatch . . . 11

2 Supporting Tables and Figures **11**

1 Mathematical details

1.1 Allelic synchronization and mRNA co-expression dynamics

1.1.1 Two single-state alleles with upstream regulation

Consider the transcriptional dynamics of 2 alleles of the same gene in a single cell. Let M_1 denote the mRNA transcript associated with allele 1, let M_2 denote the mRNA transcripts associated with allele 2, and assume that expression of both alleles are governed by linear birth-death processes with production rates $k_b^{(1)}$, $k_b^{(2)}$ and decay rates $k_d^{(1)}$, $k_d^{(2)}$. Thus, we are concerned with the dynamics of the following system of reactions:

$$\emptyset \underset{k_d^{(1)}}{\overset{k_b^{(1)}}{\rightleftarrows}} M_1 \qquad \emptyset \underset{k_d^{(2)}}{\overset{k_b^{(2)}}{\rightleftarrows}} M_2. \tag{1}$$

The number of species M_1 and M_2 are given by m_1 and m_2 respectively. Since the alleles are not coupled together they act independently and the stationary joint probability mass function (PMF) for this process is a prodcut of two independent Poisson processes:

$$p(m_1, m_2) = \frac{\lambda_1^{m_1}}{m_1!}\mathrm{e}^{-\lambda_1} \cdot \frac{\lambda_2^{m_2}}{m_2!}\mathrm{e}^{-\lambda_2}, \tag{2}$$

where $\lambda_i = k_b^{(i)}/k_d^{(i)}$ for $i \in \{1,2\}$. In order to couple the genes together we allow the transcription rates $k_b^{(1)}$ and $k_b^{(2)}$ to depend upon the concentration of a shared upstream regulator, gene X. Let x denote the concentration of X and let $\rho(x)$ be the stationary probability density function for x. Taking birth rate as $k_b^{(i)}x$, the stationary joint PMF is then obtained from Bayes' theorem:

$$\begin{aligned} p(m_1, m_2) &= \int_0^\infty p(m_1, m_2 \,|\, x)\rho(x)\, dx, \\ &= \int_0^\infty \frac{(\lambda_1 x)^{m_1}}{m_1!}\mathrm{e}^{-\lambda_1 x} \cdot \frac{(\lambda_2 x)^{m_2}}{m_2!}\mathrm{e}^{-\lambda_2 x}\rho(x)\, dx. \end{aligned} \tag{3}$$

If $x \sim \mathrm{Gamma}(r, \theta)$ then this gives

$$\begin{aligned} p(m_1, m_2) &= \int_0^\infty \frac{(\lambda_1 x)^{m_1}}{m_1!}\mathrm{e}^{-\lambda_1 x} \cdot \frac{(\lambda_2 x)^{m_2}}{m_2!}\mathrm{e}^{-\lambda_2 x} \cdot \frac{x^{r-1}e^{-\frac{x}{\theta}}}{\Gamma(r)\theta^r}\, dx, \\ &= \frac{\Gamma(m_1 + m_2 + r)}{m_1!\, m_2!\, \Gamma(r)}(1 - p - q)^r p^{m_1} q^{m_2}. \end{aligned} \tag{4}$$

where $p = \lambda_1\theta/[1+\theta(\lambda_1+\lambda_2)]$ and $q = ap$ with $a = \lambda_2/\lambda_1$. Thus, the joint PMF is a bivariate negative binomial distribution. Note that the marginal distributions are negative binomial distributions, with probability $p' = \lambda_1\theta/(1 + \lambda_1\theta)$ for allele 1 and $p'' = \lambda_2\theta/(1 + \lambda_2\theta)$ for allele 2. For instance, for allele 1:

$$\begin{aligned} p(m_1) &= \int_0^\infty p(m_1|x)\rho(x)\, dx, \\ &= \int_0^\infty \frac{(\lambda_1 x)^{m_1}}{m_1!}\mathrm{e}^{-\lambda_1 x} \cdot \frac{x^{r-1}e^{-\frac{x}{\theta}}}{\Gamma(r)\theta^r}\, dx, \\ &= \frac{\Gamma(m_1 + r)}{m_1!\Gamma(r)} p'^{m_1}(1 - p')^r. \end{aligned} \tag{5}$$

The covariance between m_1 and m_2,

$$\mathrm{Cov}(m_1, m_2) = \mathbb{E}(m_1 m_2) - \mathbb{E}(m_1)\mathbb{E}(m_2), \tag{6}$$

may be obtained from the probability generating function for $p(m_1, m_2)$, which in this case is:

$$\phi(u, v) = \mathbb{E}[u^{m_1} v^{m_2}] = [1 + \lambda_1\theta(1 - u) + \lambda_2\theta(1 - v)]^{-r}. \tag{7}$$

In particular,

$$\mathbb{E}(m_1) = \left.\frac{\partial \phi}{\partial u}\right|_{u,v=1} = \frac{rp}{1-(p+q)}, \tag{8}$$

$$\mathbb{E}(m_2) = \left.\frac{\partial \phi}{\partial v}\right|_{u,v=1} = \frac{arp}{1-(p+q)}, \tag{9}$$

$$\mathbb{E}(m_1 m_2) = \left.\frac{\partial^2 \phi}{\partial u \partial v}\right|_{u,v=1} = \frac{ap^2 r(r+1)}{(1-(p+q))^2}, \tag{10}$$

and therefore,

$$\text{Cov}(m_1, m_2) = \mathbb{E}(m_1 m_2) - \mathbb{E}(m_1)\mathbb{E}(m_2) = \frac{arp^2}{(1-(p+q))^2}. \tag{11}$$

This may be expressed in an alternative form as

$$\text{Cov}(m_1, m_2) = \lambda_1 \lambda_2 r \theta^2 = \lambda_1 \lambda_2 \text{Var}(x). \tag{12}$$

Thus, the covariance of the target genes is proportional to both the variance of the upstream regulator and the sensitivities of the two targets to the upstream regulator. The correlation between m_1 and m_2 may also be similarly calculated. We obtain:

$$\text{Corr}(m_1, m_2) = \sqrt{\frac{\lambda_1 \lambda_2 F^2(x)}{(1+\lambda_1 F(x))(1+\lambda_2 F(x))}}, \tag{13}$$

where $F(x) = \text{Var}(x)/\mathbb{E}(x)$ is the Fano factor (also known as the index of dispersion) of the upstream regulator x. Since,

$$\lim_{F(x)\to 0} \text{Corr}(m_1, m_2) = 0 \qquad \text{and} \qquad \lim_{F(x)\to \infty} \text{Corr}(m_1, m_2) = 1, \tag{14}$$

over-dispersion in the upstream regulator increases the correlation between downstream targets and under-dispersion reduces the correlation between targets. If the alleles are kinetically identical ($\lambda_1 = \lambda_2 = \lambda$) then

$$\text{Corr}(m_1, m_2) = \frac{\lambda F(x)}{1+\lambda F(x)}. \tag{15}$$

and the correlation between the alleles grows hyperbolically with the dispersion of the upstream regulator.

While the form of joint PMF given in Eq.(4) depends upon the upstream regulator being Gamma distributed, Eqs.(12)-(13) hold true for any upstream distribution $\rho(x)$ with nonnegative support. In general the probability generating function for the joint PMF $p(m_1, m_2)$ has the form:

$$\begin{aligned}
\phi(u,v) &= \sum_{m_1=0}^{\infty}\sum_{m_2=0}^{\infty} p(m_1,m_2) u^{m_1} v^{m_2}, \\
&= \sum_{m_1=0}^{\infty}\sum_{m_2=0}^{\infty} \left[\int_0^{\infty} \frac{(\lambda_1 x)^{m_1}}{m_1!} \mathrm{e}^{-\lambda_1 x} \cdot \frac{(\lambda_2 x)^{m_2}}{m_2!} \mathrm{e}^{-\lambda_2 x} \rho(x)\, dx\right] u^{m_1} v^{m_2}, \\
&= \int_0^{\infty} \mathrm{e}^{-x(\lambda_1+\lambda_2)} \rho(x) \left[\sum_{m_1=0}^{\infty}\sum_{m_2=0}^{\infty} \frac{(\lambda_1 u x)^{m_1} (\lambda_2 v x)^{m_2}}{m_1! m_2!}\right] dx, \\
&= \int_0^{\infty} \mathrm{e}^{-x(\lambda_1+\lambda_2)}\, \rho(x)\, \mathrm{e}^{x(\lambda_1 u + \lambda_2 v)}\, dx, \\
&= \int_0^{\infty} \rho(x)\, \mathrm{e}^{x(\lambda_1(u-1)+\lambda_2(v-1))}\, dx.
\end{aligned} \tag{16}$$

Thus,

$$\mathbb{E}(m_1) = \left.\frac{\partial \phi}{\partial u}\right|_{u,v=1} = \int_0^\infty \lambda_1\, x\, \rho(x)\, dx = \lambda_1 \mathbb{E}(x), \tag{17}$$

$$\mathbb{E}(m_2) = \left.\frac{\partial \phi}{\partial v}\right|_{u,v=1} = \int_0^\infty \lambda_2\, x\, \rho(x)\, dx = \lambda_2 \mathbb{E}(x), \tag{18}$$

$$\mathbb{E}(m_1 m_2) = \left.\frac{\partial^2 \phi}{\partial u \partial v}\right|_{u,v=1} = \int_0^\infty \lambda_1 \lambda_2\, x^2\, \rho(x)\, dx = \lambda_1 \lambda_2 \left(\text{Var}(x) + \mathbb{E}(x)^2\right). \tag{19}$$

Therefore,

$$\text{Cov}(m_1, m_2) = \mathbb{E}(m_1 m_2) - \mathbb{E}(m_1)\mathbb{E}(m_2) = \lambda_1 \lambda_2\, \text{Var}(x) \tag{20}$$

as before. To find the correlation of downstream targets, we also need to find Var(m_1) and Var(m_2). We do so by using:

$$\mathbb{E}(m_1(m_1 - 1)) = \left.\frac{\partial^2 \phi}{\partial u^2}\right|_{u,v=1} = \int_0^\infty \lambda_1^2\, x^2\, \rho(x)\, dx = \lambda_1^2 \left(\text{Var}(x) + \mathbb{E}(x)^2\right), \tag{21}$$

$$\mathbb{E}(m_2(m_2 - 1)) = \left.\frac{\partial^2 \phi}{\partial v^2}\right|_{u,v=1} = \int_0^\infty \lambda_2^2\, x^2\, \rho(x)\, dx = \lambda_2^2 \left(\text{Var}(x) + \mathbb{E}(x)^2\right). \tag{22}$$

Hence, as $\mathbb{E}(z(z-1)) = \text{Var}(z) - \mathbb{E}(z) - \mathbb{E}(z)^2$ we obtain

$$\text{Var}(m_1) = \mathbb{E}(m_1(m_1 - 1)) + \mathbb{E}(m_1) + \mathbb{E}(m_1)^2 = \lambda_1^2\, \text{Var}(x) + \lambda_1\, \mathbb{E}(x), \tag{23}$$

$$\text{Var}(m_2) = \mathbb{E}(m_2(m_2 - 1)) + \mathbb{E}(m_2) + \mathbb{E}(m_2)^2 = \lambda_2^2\, \text{Var}(x) + \lambda_2\, \mathbb{E}(x), \tag{24}$$

thus giving

$$\begin{aligned}\text{Corr}(m_1, m_2) &= \frac{\lambda_1 \lambda_2 \text{Var}(x)}{\sqrt{(\lambda_1^2\, \text{Var}(x) + \lambda_1\, \mathbb{E}(x))(\lambda_2^2\, \text{Var}(x) + \lambda_2\, \mathbb{E}(x))}}, \\ &= \sqrt{\frac{\lambda_1 \lambda_2 F^2(x)}{(1 + \lambda_1 F(x))(1 + \lambda_2 F(x))}},\end{aligned} \tag{25}$$

as before.

If the mRNA birth process is not linearly dependent on x, but instead is determined by some arbitrary dependence $f(x)$, then the probability generating function for $p(m_1, m_2)$ is given by

$$\phi(u, v) = \int_0^\infty \rho(x)\, \mathrm{e}^{f(x)\,(\lambda_1(u-1) + \lambda_2(v-1))}\, dx. \tag{26}$$

In this case, $\mathbb{E}(m_1)$, $\mathbb{E}(m_2)$, Var(m_1), Var(m_2) and Cov(m_1, m_2) take a similar form as above, but with $\mathbb{E}(f(x))$ and Var$(f(x))$ replacing $\mathbb{E}(x)$ and Var(x) respectively. Thus,

$$\text{Cov}(m_1, m_2) = \lambda_1 \lambda_2 \text{Var}(f(x)),$$

and

$$\text{Corr}(m_1, m_2) = \sqrt{\frac{\lambda_1 \lambda_2 F^2(f(x))}{(1 + \lambda_1 F(f(x)))(1 + \lambda_2 F(f(x)))}}. \tag{27}$$

1.1.2 One two-state allele with upstream regulation

Consider the following dynamics in which gene G transitions stochastically between in 2 different states G_+ and G_- at constant rates ω_+ and ω_-, with the rate of transcription of M depending upon the state of the gene:

$$G_- \underset{\omega_-}{\overset{\omega_+}{\rightleftarrows}} G_+, \qquad G_- \xrightarrow{k_b^-} G_- + M, \qquad G_+ \xrightarrow{k_b^+} G_+ + M, \qquad M \xrightarrow{k_d} \emptyset. \tag{28}$$

Without loss of generality we take $k_b^+ > k_b^-$. Let p_m^z denote the conditional probability $p(M = m|G = G_z)$ for $z \in \{+, -\}$. The dynamics are described by the master equation:

$$\frac{\partial p_m^z}{\partial t} = -k_b^z p_m^z - mk_d p_m^z + k_b^z p_{m-1}^z + k_d(m+1)p_{m+1}^z + \sum_{z' \neq z} \Omega_{zz'} p_m^{z'}, \tag{29}$$

where the matrix

$$\Omega_{zz'} = \begin{pmatrix} -\omega_+ & \omega_- \\ \omega_+ & -\omega_- \end{pmatrix} \tag{30}$$

is given in terms of the transition rates ω_+ and ω_- into the active and inactive states respectively. To solve Eq. (29) it is convenient to reformulate in terms of the probability generating functions $\phi_z(x) = \sum_n p_m^z x^n$, whence we obtain a pair of coupled partial differential equations for ϕ_+ and ϕ_-:

$$\frac{\partial \phi_\pm}{\partial t} = -y\left(\frac{\partial}{\partial y} - \lambda_\pm\right)\phi_\pm \pm \varepsilon_+\phi_- \mp \varepsilon_-\phi_+, \tag{31}$$

where $y = x - 1$, $\lambda_\pm = k_b^\pm/k_d$, $\varepsilon_\pm = \omega_\pm/k_d$, and we have rescaled time with the degradation rate k_d. In the limit $\varepsilon_\pm \to 0$ (i.e. transition rates between gene states are small with respect to the mRNA degradation rate) we may obtain an approximation to the stationary solution to Eq. (31) by considering an asymptotic expansion of the form $\phi_\pm = \phi_\pm^0 + \varepsilon_\pm\phi_\pm^1 + \ldots$. Substituting this anzatz into Eq. (31) we obtain $\phi_\pm^0 = \exp\left(\lambda_\pm(x-1)\right)$, which is the probability generating function for the Poisson distribution. Thus,

$$p_m^\pm = \frac{\lambda_\pm^m}{m!}\mathrm{e}^{-\lambda_\pm} + \mathcal{O}(\varepsilon_\pm). \tag{32}$$

The leading order stationary distribution $p(m)$ may then be obtained from Bayes' theorem:

$$\begin{aligned} p(m) &\sim \sum_z p(z)p(m|z), \\ &\sim \sum_z p(z)p_m^z, \\ &\sim wp_m^+ + (1-w)p_m^-, \end{aligned} \tag{33}$$

where w is the probability of the gene being in the positive state (and therefore $1 - w$ is the probability that the gene is in the negative state). By conservation of probability

$$\sum_{z'} \Omega_{zz'}\pi_{z'} = 0, \tag{34}$$

which gives $w = \omega_+/(\omega_- + \omega_+)$. Thus, in the limit $\varepsilon_\pm \to 0$ the stationary pmf for y is approximated by a Poisson mixture:

$$p(m) = w\frac{\lambda_+^m}{m!}\mathrm{e}^{-\lambda_+} + (1-w)\frac{\lambda_-^m}{m!}\mathrm{e}^{-\lambda_-} + \mathcal{O}(\varepsilon^*), \tag{35}$$

where $\varepsilon^* = \max \varepsilon_\pm$. If we now allow the transcription rates of m from each state to be proportional to the Gamma distributed concentration of the upstream regulator X as before then via Bayes theorem we obtain:

$$p(m) \approx \int_0^\infty \left[w\frac{(\lambda_+x)^m}{m!}\mathrm{e}^{-\lambda_+x} + (1-w)\frac{(\lambda_-x)^m}{m!}\mathrm{e}^{-\lambda_-x}\right] \times \left[\frac{x^{r-1}}{\theta^k\Gamma(r)}\mathrm{e}^{-x/\theta}\right] dx. \tag{36}$$

Integrating gives,

$$p(m) \sim w\frac{\Gamma(m+r)}{\Gamma(r)m!}p_1^m(1-p_1)^r + (1-w)\frac{\Gamma(m+r)}{\Gamma(r)m!}p_2^m(1-p_2)^r, \tag{37}$$

where $p_1 = \theta\lambda_+/(1+\theta\lambda_+)$ and $p_2 = \theta\lambda_-/(1+\theta\lambda_-)$. Thus, m follows a two-component negative binomial mixture, characterised by 4 parameters (w, r, p_1, p_2). This argument may be extended to a gene with n states, each with different sensitivities to the upstream regulator. In this case the target follows an n-component negative binomial mixture, i.e. $m \sim \sum_{i=1}^n w_i\mathrm{NB}(r, p_i)$, with $p_i = \theta\lambda_i/(1+\theta\lambda_i)$ and $w_i = \omega_i/\sum_i \omega_i$.

1.1.3 Two two-state alleles with upstream regulation

Now consider the following dynamics in which there are 2 genes, $G^{(i)}$ ($i = 1, 2$), which both transition stochastically between in 2 different states, $G_+^{(i)}$ and $G_-^{(i)}$, at constant rates $\omega_+^{(i)}$ and $\omega_-^{(i)}$. Let m_1 denote the number of M_1 mRNA transcripts associated with gene $G^{(1)}$; and let m_2 denote the number of M_2 mRNA transcripts associated with gene $G^{(2)}$. Both genes respond to an upstream regulator X with concentration x which is Gamma distributed. The dynamics are as follows:

$$G_-^{(i)} \underset{\omega_+^{(i)}}{\overset{\omega_-^{(i)}}{\rightleftarrows}} G_+^{(i)}, \qquad G_- \xrightarrow{k_{b-}^{(i)}x} G_- + M_i, \qquad G_+^{(i)} \xrightarrow{k_{b+}^{(i)}x} G_+^{(i)} + M_i, \qquad M_i \xrightarrow{k_d^{(i)}} \emptyset, \tag{38}$$

for $i = 1, 2$. Assuming that $\omega_+^{(i)}, \omega_-^{(i)} \ll k_d^{(i)}$ the stationary marginal distributions are both approximated by two component negative binomial mixtures, characterised by the parameters $w^{(i)}, \lambda_+^{(i)}, \lambda_-^{(i)}, r, p_1^{(i)}, p_2^{(i)}$ (exactly as before, see Eq. (37)). Since the expression of M_1 and M_2 are independent conditioned on the concentration of the upstream regulator x [i.e. $p(m_1, m_2|x) = p(m_1|x)p(m_2|x)$] the leading order stationary joint distribution is given by:

$$\begin{aligned} p(m_1, m_2) = \int_0^\infty & \left[w^{(1)} \frac{(\lambda_+^{(1)}x)^{m_1} e^{-\lambda_+^{(1)}x}}{m_1!} + (1 - w^{(1)}) \frac{(\lambda_-^{(1)}x)^{m_1} e^{-\lambda_-^{(1)}x}}{m_1!} \right] \\ & \times \left[w^{(2)} \frac{(\lambda_+^{(2)}x)^{m_2} e^{-\lambda_+^{(2)}x}}{m_2!} + (1 - w^{(2)}) \frac{(\lambda_-^{(2)}x)^{m_2} e^{-\lambda_-^{(2)}x}}{m_2!} \right] \rho(x)dx. \end{aligned} \tag{39}$$

Assuming that $x \sim \text{Gamma}(r, \theta)$ we obtain:

$$\begin{aligned} p(m_1, m_2) \sim\ & w^{(1)}w^{(2)}\text{BNB}(r, p_a, q_a) \\ & + w^{(1)}(1 - w^{(2)})\text{BNB}(r, p_b, q_b) \\ & + w^{(2)}(1 - w^{(1)})\text{BNB}(r, p_c, q_c) \\ & + (1 - w^{(1)})(1 - w^{(2)})\text{BNB}(r, p_d, q_d), \end{aligned} \tag{40}$$

where

$$\begin{aligned} p_a &= \tfrac{\lambda_+^{(1)}\theta}{1+\theta(\lambda_+^{(1)}+\lambda_+^{(2)})}, & q_a &= \alpha_a p_a, & \alpha_a &= \tfrac{\lambda_+^{(2)}}{\lambda_+^{(1)}}, \\ p_b &= \tfrac{\lambda_+^{(1)}\theta}{1+\theta(\lambda_+^{(1)}+\lambda_-^{(2)})}, & q_b &= \alpha_b p_b, & \alpha_b &= \tfrac{\lambda_-^{(2)}}{\lambda_+^{(1)}}, \\ p_c &= \tfrac{\lambda_-^{(1)}\theta}{1+\theta(\lambda_-^{(1)}+\lambda_+^{(2)})}, & q_c &= \alpha_c p_c, & \alpha_c &= \tfrac{\lambda_-^{(2)}}{\lambda_-^{(1)}}, \\ p_d &= \tfrac{\lambda_-^{(1)}\theta}{1+\theta(\lambda_-^{(1)}+\lambda_-^{(2)})}, & q_d &= \alpha_d p_d, & \alpha_d &= \tfrac{\lambda_-^{(2)}}{\lambda_-^{(1)}} \end{aligned} \tag{41}$$

and $\text{BNB}(r, p, q)$ denotes the bivariate negative binomial distribution with PMF

$$p(m_1, m_2) = \frac{\Gamma(m_1 + m_2 + r)}{m_1!\, m_2!\, \Gamma(r)} (1 - p - q)^r p^{m_1} q^{m_2}. \tag{42}$$

Following a similar process as in section 1.1.2, it is possible to generalise this result for any upstream regulator distribution. Eq. (39) can be rewritten as

$$p(m_1, m_2) = \int_0^\infty (AC + BC + AD + BD)\rho(x)dx, \tag{43}$$

where

$$\begin{aligned} A &= w^{(1)} \tfrac{(\lambda_+^{(1)}x)^{m_1} e^{-\lambda_+^{(1)}x}}{m_1!}, & B &= (1 - w^{(1)}) \tfrac{(\lambda_-^{(1)}x)^{m_1} e^{-\lambda_-^{(1)}x}}{m_1!}, \\ C &= w^{(2)} \tfrac{(\lambda_+^{(2)}x)^{m_2} e^{-\lambda_+^{(2)}x}}{m_2!}, & D &= (1 - w^{(2)}) \tfrac{(\lambda_-^{(2)}x)^{m_2} e^{-\lambda_-^{(2)}x}}{m_2!}. \end{aligned} \tag{44}$$

The probability generating function can therefore be written as:

$$\begin{aligned}
\phi(u,v) &= \sum_{m_1=0}^{\infty}\sum_{m_2=0}^{\infty} p(m_1,m_2)u^{m_1}v^{m_2}, \\
&= \sum_{m_1=0}^{\infty}\sum_{m_2=0}^{\infty}\left[\int_0^{\infty}(AC+BC+AD+BD)\rho(x)\,dx\right]u^{m_1}v^{m_2}, \\
&= \int_0^{\infty}\rho(x)\left[\sum_{m_1=0}^{\infty}\sum_{m_2=0}^{\infty} ACu^{m_1}v^{m_2}\right] + \int_0^{\infty}\rho(x)\left[\sum_{m_1=0}^{\infty}\sum_{m_2=0}^{\infty} BCu^{m_1}v^{m_2}\right] \\
&+ \int_0^{\infty}\rho(x)\left[\sum_{m_1=0}^{\infty}\sum_{m_2=0}^{\infty} ADu^{m_1}v^{m_2}\right] + \int_0^{\infty}\rho(x)\left[\sum_{m_1=0}^{\infty}\sum_{m_2=0}^{\infty} BDu^{m_1}v^{m_2}\right], \\
&= X+Y+Z+T
\end{aligned} \tag{45}$$

Where

$$\begin{aligned}
X &= \int_0^{\infty}\rho(x)\left[\sum_{m_1=0}^{\infty}\sum_{m_2=0}^{\infty} ACu^{m_1}v^{m_2}\right], \\
&= \int_0^{\infty} w^{(1)}w^{(2)}\rho(x)e^{-x(\lambda_+^{(1)}+\lambda_+^{(2)})}\sum_{m_1=0}^{\infty}\sum_{m_2=0}^{\infty}\left[\frac{(\lambda_+^{(1)}ux)^{m_1}(\lambda_+^{(2)}vx)^{m_2}}{m_1!m_2!}\right]dx, \\
&= \int_0^{\infty} w^{(1)}w^{(2)}\rho(x)e^{-x(\lambda_+^{(1)}+\lambda_+^{(2)})}e^{x(\lambda_+^{(1)}u+\lambda_+^{(2)}v)}dx, \\
&= \int_0^{\infty} w^{(1)}w^{(2)}\rho(x)e^{x\lambda_+^{(1)}(u-1)}e^{x\lambda_+^{(2)}(v-1)}dx.
\end{aligned} \tag{46}$$

Similarly,

$$Y = \int_0^{\infty}(1-w^{(1)})w^{(2)}\rho(x)e^{x\lambda_-^{(1)}(u-1)}e^{x\lambda_+^{(2)}(v-1)}dx, \tag{47}$$

$$Z = \int_0^{\infty} w^{(1)}(1-w^{(2)})\rho(x)e^{x\lambda_+^{(1)}(u-1)}e^{x\lambda_-^{(2)}(v-1)}dx, \tag{48}$$

$$T = \int_0^{\infty}(1-w^{(1)})(1-w^{(2)})\rho(x)e^{x\lambda_-^{(1)}(u-1)}e^{x\lambda_-^{(2)}(v-1)}dx. \tag{49}$$

Expected values are found from the probability generating function:

$$\mathbb{E}(m_1) = \left.\frac{\partial\phi}{\partial u}\right|_{u,v=1} = \left.\frac{\partial X}{\partial u}\right|_{u,v=1} + \left.\frac{\partial Y}{\partial u}\right|_{u,v=1} + \left.\frac{\partial Z}{\partial u}\right|_{u,v=1} + \left.\frac{\partial T}{\partial u}\right|_{u,v=1}. \tag{50}$$

As

$$\begin{aligned}
\left.\frac{\partial X}{\partial u}\right|_{u,v=1} &= w^{(1)}w^{(2)}\int_0^{\infty}\rho(x)x\lambda_+^{(1)}dx = w^{(1)}w^{(2)}\lambda_+^{(1)}\mathbb{E}(x), \\
\left.\frac{\partial Y}{\partial u}\right|_{u,v=1} &= (1-w^{(1)})w^{(2)}\int_0^{\infty}\rho(x)x\lambda_-^{(1)}dx = (1-w^{(1)})w^{(2)}\lambda_-^{(1)}\mathbb{E}(x), \\
\left.\frac{\partial Z}{\partial u}\right|_{u,v=1} &= w^{(1)}(1-w^{(2)})\int_0^{\infty}\rho(x)x\lambda_+^{(1)}dx = w^{(1)}(1-w^{(2)})\lambda_+^{(1)}\mathbb{E}(x), \\
\left.\frac{\partial T}{\partial u}\right|_{u,v=1} &= (1-w^{(1)})(1-w^{(2)})\int_0^{\infty}\rho(x)x\lambda_-^{(1)}dx = (1-w^{(1)})(1-w^{(2)})\lambda_-^{(1)}\mathbb{E}(x).
\end{aligned} \tag{51}$$

This results in:

$$\mathbb{E}(m_1) = (w^{(1)}\lambda_+^{(1)} + (1 - w^{(1)})\lambda_-^{(1)})\mathbb{E}(x). \tag{52}$$

Similarly,

$$\mathbb{E}(m_2) = (w^{(2)}\lambda_+^{(2)} + (1 - w^{(2)})\lambda_-^{(2)})\mathbb{E}(x). \tag{53}$$

In order to find the covariance of the joint distribution we also need to calculate $\mathbb{E}(m_1, m_2)$:

$$\mathbb{E}(m_1, m_2) = \frac{\partial \phi}{\partial u, \partial v}\bigg|_{u,v=1} = \frac{\partial X}{\partial u, \partial v}\bigg|_{u,v=1} + \frac{\partial Y}{\partial u, \partial v}\bigg|_{u,v=1} + \frac{\partial Z}{\partial u, \partial v}\bigg|_{u,v=1} + \frac{\partial T}{\partial u, \partial v}\bigg|_{u,v=1}. \tag{54}$$

Using,

$$\begin{aligned}
\frac{\partial X}{\partial u, \partial v}\bigg|_{u,v=1} &= w^{(1)}w^{(2)}\lambda_+^{(1)}\lambda_+^{(2)}\int_0^\infty x^2\rho(x)dx = w^{(1)}w^{(2)}\lambda_+^{(1)}\lambda_+^{(2)}(\text{Var(x)} + \mathbb{E}(x)^2),\\
\frac{\partial Y}{\partial u, \partial v}\bigg|_{u,v=1} &= (1 - w^{(1)})w^{(2)}\lambda_-^{(1)}\lambda_+^{(2)}\int_0^\infty x^2\rho(x)dx = (1 - w^{(1)})w^{(2)}\lambda_-^{(1)}\lambda_+^{(2)}(\text{Var(x)} + \mathbb{E}(x)^2),\\
\frac{\partial Z}{\partial u, \partial v}\bigg|_{u,v=1} &= w^{(1)}(1 - w^{(2)})\lambda_+^{(1)}\lambda_-^{(2)}\int_0^\infty x^2\rho(x)dx = w^{(1)}(1 - w^{(2)})\lambda_+^{(1)}\lambda_-^{(2)}(\text{Var(x)} + \mathbb{E}(x)^2),\\
\frac{\partial T}{\partial u, \partial v}\bigg|_{u,v=1} &= (1 - w^{(1)})(1 - w^{(2)})\lambda_-^{(1)}\lambda_-^{(2)}\int_0^\infty x^2\rho(x)dx = (1 - w^{(1)})(1 - w^{(2)})\lambda_-^{(1)}\lambda_-^{(2)}(\text{Var(x)} + \mathbb{E}(x)^2),
\end{aligned} \tag{55}$$

it can be seen that,

$$\mathbb{E}(m_1, m_2) = (w^{(1)}\lambda_+^{(1)} + (1 - w^{(1)})(w^{(2)}\lambda_+^{(2)} + (1 - w^{(2)})\lambda_-^{(2)}))(\text{Var}(x) + \mathbb{E}(x)^2). \tag{56}$$

In general, the covariance between the output mRNA of two bursting genes is given by:

$$\begin{aligned}
\text{Cov}(m_1, m_2) &= \mathbb{E}(m_1, m_2) - \mathbb{E}(m_1)\mathbb{E}(m_2),\\
&= (w^{(1)}\lambda_+^{(1)} + (1 - w^{(1)})(w^{(2)}\lambda_+^{(2)} + (1 - w^{(2)})\lambda_-^{(2)}))\,\text{Var}(x).
\end{aligned} \tag{57}$$

For two alleles of the same gene, we assume that the switching rates between gene states and the transcription rates from each state are the same; $\lambda_+^{(1)} = \lambda_+^{(2)} = \lambda_+$, $\lambda_-^{(1)} = \lambda_-^{(2)} = \lambda_-$ and $w^{(1)} = w^{(2)} = w$. Hence,

$$\text{Cov}(m_1, m_2) = (w\lambda_+ + (1 - w)\lambda_-)^2\,\text{Var}(x). \tag{58}$$

As for one-state genes, the covariance between allelic mRNA outputs of two-state genes is dependent on the variance of a common upstream regulator. Fig. S1 demonstrates how increasing variance of an upstream regulator (shown for Gamma-distributed regulator concentration, x), affects the joint distribution of downstream alleles for a two-state gene and increases covariance.

1.2 Bifurcation curves for Nanog dynamics

The dynamics for all the reporter strategies that we consider can be described by the following dimensionless ordinary differential equation (ODE) for total Nanog concentration $\bar{n}$ (see main text and below),

$$\frac{d\bar{n}}{d\tau} = \alpha + \frac{\bar{n}^H}{\gamma^H + \bar{n}^H} - \bar{n}. \tag{59}$$

Fixed points solutions, in which $d\bar{n}/d\tau = 0$, satisfy the polynomial

$$0 = \gamma^H(\alpha - \bar{n}) + (\alpha + 1)\bar{n}^H - \bar{n}^{H+1}. \tag{60}$$

This polynomial has either 1 or three real solutions, depending on the values of α and γ and the Hill coefficient H. When there is only one real solution the system has one stable fixed point and the resulting Nanog is unimodal; when there are three real

solutions, two of them are stable and the Nanog distribution is bimodal. The threshold between these regimes occurs when there is a repeated solution, which occurs when the discriminant Δ of Eq. (60) is zero. In the case $H = 2$

$$\Delta = \gamma^2 + \left(2\alpha^2 - 5\alpha - \frac{1}{4}\right)\gamma + \alpha\left(1 + \alpha^3\right). \tag{61}$$

Thus $\Delta = 0$ is a quadratic for γ which has roots

$$\gamma_{\pm}(\alpha) = -\left(\alpha^2 - \frac{5}{2}\alpha - \frac{1}{8}\right) \pm \left(\frac{1}{4} - 2\alpha\right)^{\frac{3}{2}}, \tag{62}$$

which are the bifurcation curves given in the main text. If the model parameters fall *inside* the region enclosed by these curves then Nanog expression is bimodal, corresponding to the coexistence of a Nanog high and Nanog low expressing sub-populations of cells; if the model parameters fall *outside* this region then Nanog expression is unimodal, corresponding to a homogeneous population of Nanog high or Nanog low expressing cells. A similar calculation may be performed for arbitrary $H \in \mathbb{Z}^+$.

1.3 Reporter perturbations

To understand how reporters may affect endogeneous Nanog dynamics we compared the dynamics of Nanog in various different reporter lines with those in wild-type ES cells. To recap from the main text, Nanog expression in wild-type cells is described by the following ODEs:

$$\frac{dn_1}{dt} = c_b + \frac{c_f n^H}{K^H + n^H} - c_d n_1, \tag{63}$$

$$\frac{dn_2}{dt} = c_b + \frac{c_f n^H}{K^H + n^H} - c_d n_2, \tag{64}$$

where n_i denotes the concentration of the Nanog protein output of allele $i \in \{1, 2\}$, $n = (n_1 + n_2)$ is total Nanog concentration. Combining these equations we obtain an ODE for the total Nanog protein concentration:

$$\frac{dn}{dt} = 2c_b + 2\frac{c_f n^H}{K^H + n^H} - c_d n \tag{65}$$

Nondimensionalizing using the scalings $n = 2c_f c_d^{-1}\bar{n}$, and $t = c_d^{-1}\tau$ we obtain:

$$\frac{d\bar{n}}{d\tau} = \alpha + \frac{\bar{n}^H}{\gamma_{\text{wt}}^H + \bar{n}^H} - \bar{n} \tag{66}$$

where $\bar{n}$ is the dimensionless total Nanog concentration and τ is dimensionless time. The dimensionless constants $\alpha = c_b/c_f$ and $\gamma = \gamma_{\text{wt}} = c_d K/2c_f$ measure the strength of the baseline production rate and the strength of the Nanog autoregulatory feedback loop respectively. We now consider how Nanog dynamics given by Eq.(66) are perturbed by a variety of different kinds of reporters. In all cases, for clarity of exposition, the reporter proteins are assumed to decay with the same kinetics as Nanog. This assumption may be weakened without affecting our conclusions. The results of this section are also summarised in Supporting Tables 1 & 2 which also give the relationships between reporter and Nanog concentrations at equilibrium as a measure of the quantitative accuracy of each reporter.

1.4 Single allele reporter strategies

1.4.1 Knock-in reporters

Knock-in reporters reporters remove the Nanog protein coding region from one allele and replace it with a reporter gene under the same promoter control. In this case, the kinetics described by Eqs. (65) are modified to

$$\frac{dn}{dt} = c_b + \frac{c_f n^H}{K^H + n^H} - c_d n, \tag{67}$$

$$\frac{dr}{dt} = c_b + \frac{c_f n^H}{K^H + n^H} - c_d r, \tag{68}$$

where n denotes Nanog concentration and r denotes reporter concentration. Using the scalings $n = c_f c_d^{-1}\bar{n}$ and $t = c_d^{-1}\tau$ the dimensionless equation for total Nanog concentration in the knock-in reporter line is:

$$\frac{d\bar{n}}{d\tau} = \alpha + \frac{\bar{n}^H}{\gamma_{\text{ki}}^H + \bar{n}^H} - \bar{n}, \tag{69}$$

where α is as before, but $\gamma_{\text{ki}} = 2\gamma_{\text{wt}}$. In this case, the loss of Nanog production from one allele has the effect of diminishing the functional Nanog production rate by a factor of two, which effectively weakens the endogenous feedback mechanisms and thereby doubles γ. Since the magnitude of γ determines if Nanog is homogeneously or heterogeneously expressed in the population, this change can induce a heterogeneous Nanog expression pattern in a reporter cell line that is not characteristic of the wild-type (or vice versa). From Eqs. (67)-(68) at equilibrium $r = n$ so it is expected that the knock-in reporter signal will faithfully represent Nanog expression in the engineered line.

1.4.2 Pre/post (PP) reporters

Single allele pre/post reporters insert the reporter gene either directly before or after the Nanog protein coding region on one Nanog allele. We assume that this insertion alters the Nanog production rate from the reporter allele by a factor $0 \leq \epsilon$. The following ODEs describe the dynamics in this case:

$$\frac{dn_1}{dt} = c_b + \frac{c_f n^H}{K^H + n^H} - c_d n_1, \tag{70}$$

$$\frac{dn_2}{dt} = \epsilon c_b + \epsilon \frac{c_f n^H}{K^H + n^H}, -c_d n_2 \tag{71}$$

$$\frac{dr}{dt} = \epsilon c_b + \epsilon \frac{c_f n^H}{K^H + n^H}, -c_d r \tag{72}$$

where n_i denotes the concentration of the Nanog protein output of allele $i \in \{1, 2\}$ and r denotes the reporter concentration (assumed without loss of generality to be produced from allele 2). Combining these equations for total Nanog $n = n_1 + n_2$ and using the scalings $n = c_f(1 + \epsilon)c_d^{-1}\bar{n}$ and $t = c_d^{-1}\tau$, the dimensionless equation for total Nanog is:

$$\frac{d\bar{n}}{d\tau} = \alpha + \frac{\bar{n}^H}{\gamma_{\text{pp}}^H + \bar{n}^H} - \bar{n}, \tag{73}$$

where $\gamma_{\text{pp}} = 2\gamma_{\text{wt}}/(1+\epsilon)$. Thus, if the addition of the reporter gene completely halts transcription from allele 2 then $\epsilon = 0$ and $\gamma_{\text{pp}} = \gamma_{\text{ki}} = 2\gamma_{\text{wt}}$; if the reporter halves the rate of transcription from allele 2, as in Fig. 2C, then $\epsilon = 1/2$ and $\gamma_{\text{pp}} = 4\gamma_{\text{wt}}/3$; if the reporter does not affect the rate of transcription from allele 1, then $\epsilon = 1$ and $\gamma_{\text{pp}} = \gamma_{\text{wt}}$. For $0 < \epsilon < 1$ pre/post reporters are less likely than knock-in reporters to induce qualitative changes in Nanog expression dynamics, yet are still subject to similar systemic risk. Similar results are obtained for $1 < \epsilon$, see Fig. S2. From Eqs. (70)-(72) at equilibrium $r = \epsilon n/(1+\epsilon)$ so it is expected that, in addition to any qualitative perturbations, the PP reporter signal will quantitatively misrepresent Nanog expression by a factor $\epsilon/(1 + \epsilon)$.

1.4.3 Multiple pre/post (MPP) reporters

If multiple (m) repeats of the reporter gene are inserted on the reporter allele then we assume that any production rates changes due to the reporter construct are compounded and transcription rate is altered by a factor ϵ_m where $0 \leq \epsilon_m \leq \epsilon < 1$ for a rate decrease and $1 < \epsilon \leq \epsilon_m$ for a rate increase. If m copies of the reporter transcript are produced (for non-tandem repeats) then the dynamics become

$$\frac{dn_1}{dt} = c_b + \frac{c_f n^H}{K^H + n^H} - c_d n_1, \tag{74}$$

$$\frac{dn_2}{dt} = \epsilon_m c_b + \epsilon_m \frac{c_f n^H}{K^H + n^H} - c_d n_2, \tag{75}$$

$$\frac{dr}{dt} = m\epsilon_m c_b + m\epsilon_m \frac{c_f n^H}{K^H + n^H} - c_d r. \tag{76}$$

Combining these equations and using the scalings $n = c_f(1 + \epsilon_m)c_d^{-1}\bar{n}$ and $t = c_d^{-1}\tau$, the dimensionless equation for total Nanog is:

$$\frac{d\bar{n}}{d\tau} = \alpha + \frac{\bar{n}^H}{\gamma_{\text{mpp}}^H + \bar{n}^H} - \bar{n}, \tag{77}$$

where $\gamma_{\text{mpp}} = 2\gamma_{\text{wt}}/(1+\epsilon_m)$. If a single insert slows transcription by a factor $0 \leq \epsilon \leq 1$ and each of the m inserts is identical, then $\epsilon_m = \epsilon/(m(1-\epsilon)+\epsilon) \leq \epsilon$ (with equality if and only if $m = 1$). Thus, although multiple reporter additions improve fluorescent signal, the systemic risk is increased with each additional reporter insert. As m becomes large $\epsilon_m \to 0$ and this risk approaches that of the knock-in reporters. From Eqs. (74)-(76) at equilibrium $r = m\epsilon_m n/(1+\epsilon_m)$ so it is expected that, in addition to any qualitative perturbations, the MPP reporter signal will quantitatively misrepresent Nanog expression by a factor $m\epsilon_m/(1+\epsilon_m)$.

1.4.4 Fusion reporters

Fusion reporters produce a modified version of Nanog, which includes a fluorescence structure as part of the Nanog protein. For single allele fusion reporters, the fusion protein (concentration n_2) is produced from one allele, and the wild-type protein (concentration n_1) is produced from the other. This has two effects on the dynamics: (1) the rate of transcription from the reporter allele is reduced by a factor $0 \leq \epsilon \leq 1$ due to the additional DNA that must be transcribed, as for a PP reporter, and (2) the function of the Nanog from the reporter allele is compromised by a factor $0 \leq \delta \leq 1$ due to the addition of a cumbersome fluorescent protein to the native Nanog. The dynamics in this case are:

$$\frac{dn_1}{dt} = c_b + \frac{c_f n_{\text{eff}}^H}{K^H + n_{\text{eff}}^H} - c_d n_1, \tag{78}$$

$$\frac{dn_2}{dt} = \epsilon c_b + \epsilon \frac{c_f n_{\text{eff}}^H}{K^H + n_{\text{eff}}^H} - c_d n_2, \tag{79}$$

where $n_{\text{eff}} = n_1 + \delta n_2$. Combining these equations and nondimensionalising using the scalings $n_{\text{eff}} = c_f(1+\epsilon\delta)c_d^{-1}\bar{n}_{\text{eff}}$ and $t = c_d^{-1}\tau$ we obtain the following equation for the effective Nanog concentration:

$$\frac{d\bar{n}_{\text{eff}}}{d\tau} = \alpha + \frac{\bar{n}_{\text{eff}}^H}{\gamma_{\text{fus}}^H + \bar{n}_{\text{eff}}^H} - \bar{n}_{\text{eff}}, \tag{80}$$

where $\gamma_{\text{fus}} = 2\gamma_{\text{wt}}/(1+\epsilon\delta)$. If $\delta = 0$ then the Nanog-reporter fusion is not functional, while for $\delta = 1$ the Nanog-reporter fusion functions as the native Nanog protein. For $0 < \delta < 1$, $\gamma_{\text{fus}} > \gamma_{\text{pp}}$, therefore fusion reporters are more likely than pre/post reporters to induce qualitative changes in expression dynamics. From Eqs. (78)-(79) at equilibrium $r = \epsilon n/(1+\epsilon)$ so it is expected that, in addition to any qualitative perturbations, the fusion reporter signal will quantitatively misrepresent Nanog expression by a factor $r = \epsilon/(1+\epsilon)$.

1.4.5 BAC reporters

Bacterial artificial chromosome (BAC) reporters introduce a piece of extra-genomic DNA into the cell that encodes the Nanog gene under the control of the endogenous Nanog promoter and regulatory regions. Because this construct does not disturb the kinetics of either of the wild-type alleles, it (uniquely amongst the reporters we consider) does not directly affect the endogenous feedback mechanisms and is therefore the least likely reporter strategy to induce qualitative changes in Nanog dynamics. However, because the reporter construct is physically separated from the Nanog alleles, it is expected that the reporter protein expression is subject to extrinsic stochastic fluctuations which are independent to those of endogenous Nanog expression. For this reason we expect that BAC reporters are more susceptible to *technical* errors that the other constructs we consider.

1.5 Dual allele reporter strategies

Dual allele reporters can either express the same reporter molecule from both allele (e.g. both drive transcription of GFP) or may express different reporter molecules from different alleles (e.g. GFP from one allele, and a red fluorescent protein from the other). The analysis of the single allele reporters above may be easily modified to account for dual reporter strategies. The dynamics for total Nanog in dual reporter systems are given by:

$$\frac{d\bar{n}}{d\tau} = \alpha + \frac{\bar{n}^H}{\gamma_{\text{dual}}^H + \bar{n}^H} - \bar{n}, \tag{81}$$

where: (1) $\gamma_{\text{dual}} = \gamma_{\text{wt}}/\epsilon_m$ in the case of dual multiple pre/post reporters that produce m copies of the same fluorescent signal from both alleles (which reduces the rate of transcription from both alleles by a factor $0 \leq \epsilon_m \leq 1$ from both alleles); (2) $\gamma_{\text{dual}} = 2\gamma_{\text{wt}}/(\epsilon_{m1} + \epsilon_{m2})$ in the case of dual pre/post reporters that produce different reporters from the two alleles

(which reduces the rate of transcription from alleles 1 and 2 by factors $0 \leq \epsilon_{m1} \leq 1$ and $0 \leq \epsilon_{m2} \leq 1$ respectively); (3) $\gamma_{\text{dual}} = \gamma_{\text{wt}}/\epsilon\delta$ for dual fusion reporters that produce the same fusion protein from each allele (which reduces the rate of transcription from both alleles by a factor $0 \leq \epsilon \leq 1$ from both alleles, and compromises Nanog function by a factor δ); (4) $\gamma_{\text{dual}} = 2\gamma_{\text{wt}}/(\epsilon_1\delta_1 + \epsilon_2\delta_2)$ for dual fusion reporters that produce different fusion proteins from each allele (which reduce the rates of transcription by factors $0 \leq \epsilon_1 \leq 1$ and $0 \leq \epsilon_2 \leq 1$ from alleles 1 and 2 respectively, and compromise Nanog function by factors δ_1 and δ_2 respectively). It should be noted for dual allele fusion reporters there is no wild-type protein in the system at all which could have further unintended consequences, including off target effects. In all cases γ_{dual} is larger than the corresponding value of γ for the single allele reporters. Thus, while more technically accurate, dual allele reporters carry raised risk of systemic perturbations to the endogenous kinetics. In addition to any qualitative perturbations, dual reporter systems may also quantitatively misrepresent Nanog expression in similar ways to the corresponding single allele constructs. These perturbations are detailed in Supporting Table 2.

1.6 Decay constant mismatch

All the simplified reporters described above have identical decay rate constants for the reporter protein and Nanog. The effect of mismatched decay rates can be seen by examining the BAC reporter, which does not perturb α or γ. Governing ODEs are given below when Nanog and reporter molecules have decay rate constants c_{dn} and c_{dr} respectively:

$$\frac{dn}{dt} = 2c_b + 2\frac{c_f n^H}{K^H + n^H} - c_{dn}n, \tag{82}$$

$$\frac{dr}{dt} = c_b + \frac{c_f n^H}{K^H + n^H} - c_{dr}r. \tag{83}$$

Non-dimensionalisation using the scalings $t = c_{dn}^{-1}\tau$, $n = 2c_f c_{dn}^{-1}\bar{n}$ and $r = c_f c_{dr}^{-1}\bar{r}$, leads to the following dimensionless ODEs:

$$\frac{d\bar{n}}{d\tau} = \alpha + \frac{\bar{n}^H}{\gamma_d^H + \bar{n}^H} - \bar{n},$$

$$\frac{c_{dn}}{c_{dr}}\frac{d\bar{r}}{d\tau} = \alpha + \frac{\bar{n}^H}{\gamma_d^H + \bar{n}^H} - \bar{r}, \tag{84}$$

where $\gamma_d = \gamma_{\text{wt}} = c_{dn}K/2c_f$. Since the qualitative nature of the dynamics depends upon solutions to $d\bar{n}/d\tau = 0$, decay mismatch does not alter expression patterns qualitatively. However, the reporter concentration does depend quantitatively on the ratio c_{dr}/c_{dn}. In the same way, in PP and MPP reporters, if the reporter protein(s) have decay constants that are different to that of Nanog, then this will not change to α or γ, and so will not change the dynamics qualitatively. By contrast, in fusion reporters if the Nanog-reporter fusion has a different decay rate to that of the wild-type Nanog then there is the potential to cause a qualitative perturbation to the dynamics, as the value of c_{dn} and hence γ will be altered.

2 Supporting Tables and Figures

Supplementary Table 1

Reporter type	ODEs	Dimensionless variables	γ	Dimensionless ODEs	Reporter-Nanog relationship at eqbm.
BAC	$\frac{dn}{dt} = 2c_b + 2\frac{c_f n^H}{K^H + n^H} - c_d n$<br>$\frac{dr}{dt} = c_b + \frac{c_f n^H}{K^H + n^H} - c_d r$	$n = \frac{2c_f}{c_d}\bar{n}$<br>$r = \frac{c_f}{c_d}\bar{r}$	$\gamma_{\mathrm{b}} = \gamma_{\mathrm{wt}}$	$\frac{d\bar{n}}{d\tau} = \alpha + \frac{\bar{n}^H}{\gamma_{\mathrm{b}}^H + \bar{n}^H} - \bar{n}$<br>$\frac{d\bar{r}}{d\tau} = \alpha + \frac{\bar{n}^H}{\gamma_{\mathrm{b}}^H + \bar{n}^H} - \bar{r}$	$r = \frac{n}{2}$
Knock-in	$\frac{dn}{dt} = c_b + c_f\frac{n^H}{K^H + n^H} - c_d n$<br>$\frac{dr}{dt} = c_b + c_f\frac{n^H}{K^H + n^H} - c_d r$	$n = \frac{c_f}{c_d}\bar{n}$<br>$r = \frac{c_f}{c_d}\bar{r}$	$\gamma_{\mathrm{ki}} = 2\gamma_{\mathrm{wt}}$	$\frac{d\bar{n}}{d\tau} = \alpha + \frac{\bar{n}^H}{\gamma_{\mathrm{ki}}^H + \bar{n}^H} - \bar{n}$<br>$\frac{d\bar{r}}{d\tau} = \alpha + \frac{\bar{n}^H}{\gamma_{\mathrm{ki}}^H + \bar{n}^H} - \bar{r}$	$r = n$
Pre/post	$\frac{dn_1}{dt} = c_b + c_f\frac{n^H}{K^H + n^H} - c_d n_1$<br>$\frac{dn_2}{dt} = \epsilon c_b + \epsilon c_f\frac{n^H}{K^H + n^H} - c_d n_2$<br>$\frac{dr}{dt} = \epsilon c_b + \epsilon c_f\frac{n^H}{K^H + n^H} - c_d r$<br>$\frac{dn}{dt} = (1+\epsilon)\big(c_b + c_f\frac{n^H}{K^H + n^H}\big) - c_d n$	$n = \frac{c_f(1+\epsilon)}{c_d}\bar{n}$<br>$r = \frac{c_f\epsilon}{c_d}\bar{r}$	$\gamma_{\mathrm{pp}} = \frac{2\gamma_{\mathrm{wt}}}{1+\epsilon}$	$\frac{d\bar{n}}{d\tau} = \alpha + \frac{\bar{n}^H}{\gamma_{\mathrm{pp}}^H + \bar{n}^H} - \bar{n}$<br>$\frac{d\bar{r}}{d\tau} = \alpha + \frac{\bar{n}^H}{\gamma_{\mathrm{pp}}^H + \bar{n}^H} - \bar{r}$	$r = \frac{\epsilon n}{1+\epsilon}$
Fusion	$\frac{dn_1}{dt} = c_b + c_f\frac{n_{\mathrm{eff}}^H}{K^H + n_{\mathrm{eff}}^H} - c_d n_1$<br>$\frac{dn_2}{dt} = \epsilon c_b + \epsilon c_f\frac{n_{\mathrm{eff}}^H}{K^H + n_{\mathrm{eff}}^H} - c_d n_2$<br>$\frac{dn}{dt} = (1+\epsilon)\big(c_b + c_f\frac{n_{\mathrm{eff}}^H}{K^H + n_{\mathrm{eff}}^H}\big) - c_d n$<br>$\frac{dn_{\mathrm{eff}}}{dt} = (1+\epsilon\delta)\big(c_b + c_f\frac{n_{\mathrm{eff}}^H}{K^H + n_{\mathrm{eff}}^H}\big) - c_d n_{\mathrm{eff}}$	$n_1 = \frac{c_f(1+\epsilon\delta)}{c_d}\bar{n}_1$<br>$n_2 = \frac{c_f(1+\epsilon\delta)}{c_d}\bar{n}_2$<br>$n = \frac{c_f(1+\epsilon\delta)}{c_d}\bar{n}$<br>$n_{\mathrm{eff}} = \frac{c_f(1+\epsilon\delta)}{c_d}\bar{n}_{\mathrm{eff}}$	$\gamma_{\mathrm{fus}} = \frac{2\gamma_{\mathrm{wt}}}{1+\epsilon\delta}$	$\frac{d\bar{n}_1}{d\tau} = \frac{1}{1+\epsilon\delta}\big(\alpha + \frac{\bar{n}_{\mathrm{eff}}^H}{\gamma_{\mathrm{fus}}^H + \bar{n}_{\mathrm{eff}}^H}\big) - \bar{n}_1$<br>$\frac{d\bar{n}_2}{d\tau} = \frac{\epsilon}{1+\epsilon\delta}\big(\alpha + \frac{\bar{n}_{\mathrm{eff}}^H}{\gamma_{\mathrm{fus}}^H + \bar{n}_{\mathrm{eff}}^H}\big) - \bar{n}_2$<br>$\frac{d\bar{n}}{d\tau} = \frac{1+\epsilon}{1+\epsilon\delta}\big(\alpha + \frac{\bar{n}_{\mathrm{eff}}^H}{\gamma_{\mathrm{fus}}^H + \bar{n}_{\mathrm{eff}}^H}\big) - \bar{n}$<br>$\frac{d\bar{n}_{\mathrm{eff}}}{d\tau} = \alpha + \frac{\bar{n}_{\mathrm{eff}}^H}{\gamma_{\mathrm{fus}}^H + \bar{n}_{\mathrm{eff}}^H} - \bar{n}_{\mathrm{eff}}$	$r = n_2 = \frac{\epsilon n}{1+\epsilon}$

Supporting Table 1: **Perturbations by single allele reporters.** Summary of analysis of reporter perturbations to wild-type dynamics for single allele reporters.

Supplementary Table 2

Reporter type	ODEs	Dimensionless variables	γ		Dimensionless ODEs	Reporter-Nanog relation at eqbm.	
			Same reporters	Different reporters		Same reporters	Different reporters
Pre/post	$\frac{dn_1}{dt} = \epsilon_1 c_b + \epsilon_1 c_f \frac{n^H}{K^H + n^H} - c_d n_1$<br>$\frac{dn_2}{dt} = \epsilon_2 c_b + \epsilon_2 c_f \frac{n^H}{K^H + n^H} - c_d n_2$<br>$\frac{dr_1}{dt} = \epsilon_1 c_b + \epsilon_1 c_f \frac{n^H}{K^H + n^H} - c_d r_1$<br>$\frac{dr_2}{dt} = \epsilon_2 c_b + \epsilon_2 c_f \frac{n^H}{K^H + n^H} - c_d r_2$<br>$\frac{dn}{dt} = (\epsilon_1 + \epsilon_2)(c_b + c_f \frac{n^H}{K^H + n^H}) - c_d n$	$n = \frac{c_f(\epsilon_1 + \epsilon_2)}{c_d}\bar{n}$<br>$r_1 = \frac{c_f \epsilon_1}{c_d}\bar{r}_1$<br>$r_2 = \frac{c_f \epsilon_2}{c_d}\bar{r}_2$	$\frac{\gamma_{\text{wt}}}{\epsilon}$	$\frac{2\gamma_{\text{wt}}}{\epsilon_1 + \epsilon_2}$	$\frac{d\bar{n}}{d\tau} = \alpha + \frac{\bar{n}^H}{\gamma_{\text{pp2}}^H + \bar{n}^H} - \bar{n}$<br>$\frac{d\bar{r}_1}{d\tau} = \alpha + \frac{\bar{n}^H}{\gamma_{\text{pp2}}^H + \bar{n}^H} - \bar{r}_1$<br>$\frac{d\bar{r}_2}{d\tau} = \alpha + \frac{\bar{n}^H}{\gamma_{\text{pp2}}^H + \bar{n}^H} - \bar{r}_2$	$r = n$	$r = r_1 + r_2 = n$<br>$r_1 = \frac{\epsilon_1 n}{\epsilon_1 + \epsilon_2}$<br>$r_2 = \frac{\epsilon_2 n}{\epsilon_1 + \epsilon_2}$
Fusion	$\frac{dn_1}{dt} = \epsilon_1 c_b + \epsilon_1 c_f \frac{n_{\text{eff}}^H}{K^H + n_{\text{eff}}^H} - c_d n_1$<br>$\frac{dn_2}{dt} = \epsilon_2 c_b + \epsilon_2 c_f \frac{n_{\text{eff}}^H}{K^H + n_{\text{eff}}^H} - c_d n_2$<br>$\frac{dn}{dt} = (\epsilon_1 + \epsilon_2)(c_b + c_f \frac{n_{\text{eff}}^H}{K^H + n_{\text{eff}}^H}) - c_d n$<br>$\frac{dn_{\text{eff}}}{dt} = (\epsilon_1\delta_1 + \epsilon_1\delta_2)(c_b + c_f \frac{n_{\text{eff}}^H}{K^H + n_{\text{eff}}^H}) - c_d n_{\text{eff}}$	$n_1 = \frac{c_f(\epsilon_1\delta_1 + \epsilon_2\delta_2)}{c_d}\bar{n}_1$<br>$n_2 = \frac{c_f(\epsilon_1\delta_1 + \epsilon_2\delta_2)}{c_d}\bar{n}_2$<br>$n = \frac{c_f(\epsilon_1\delta_1 + \epsilon_2\delta_2)}{c_d}\bar{n}$<br>$n_{\text{eff}} = \frac{c_f(\epsilon_1\delta_1 + \epsilon_2\delta_2)}{c_d}\bar{n}_{\text{eff}}$	$\frac{\gamma_{\text{wt}}}{\epsilon\delta}$	$\frac{2\gamma_{\text{wt}}}{\epsilon_1\delta_1 + \epsilon_2\delta_2}$	$\frac{d\bar{n}_1}{d\tau} = \frac{\epsilon_1}{\epsilon_1\delta_1 + \epsilon_2\delta_2}(\alpha + \frac{\bar{n}_{\text{eff}}^H}{\gamma_{\text{fus2}}^H + \bar{n}_{\text{eff}}^H}) - \bar{n}_1$<br>$\frac{d\bar{n}_2}{d\tau} = \frac{\epsilon_2}{\epsilon_1\delta_1 + \epsilon_2\delta_2}(\alpha + \frac{\bar{n}_{\text{eff}}^H}{\gamma_{\text{fus2}}^H + \bar{n}_{\text{eff}}^H}) - \bar{n}_2$<br>$\frac{d\bar{n}}{d\tau} = \frac{\epsilon_1 + \epsilon_2}{\epsilon_1\delta_1 + \epsilon_2\delta_2}(\alpha + \frac{\bar{n}_{\text{eff}}^H}{\gamma_{\text{fus2}}^H + \bar{n}_{\text{eff}}^H}) - \bar{n}$<br>$\frac{d\bar{n}_{\text{eff}}}{d\tau} = \alpha + \frac{\bar{n}_{\text{eff}}^H}{\gamma_{\text{fus2}}^H + \bar{n}_{\text{eff}}^H} - \bar{n}_{\text{eff}}$	$r = n$	$r = n_1 + n_2 = n$<br>$n_1 = \frac{\epsilon_1 n}{\epsilon_1 + \epsilon_2}$<br>$n_2 = \frac{\epsilon_2 n}{\epsilon_1 + \epsilon_2}$

Supporting Table 2: **Perturbations by dual allele reporters.** Summary of analysis of reporter perturbations to wild-type dynamics for dual allele reporters.

Figure S1

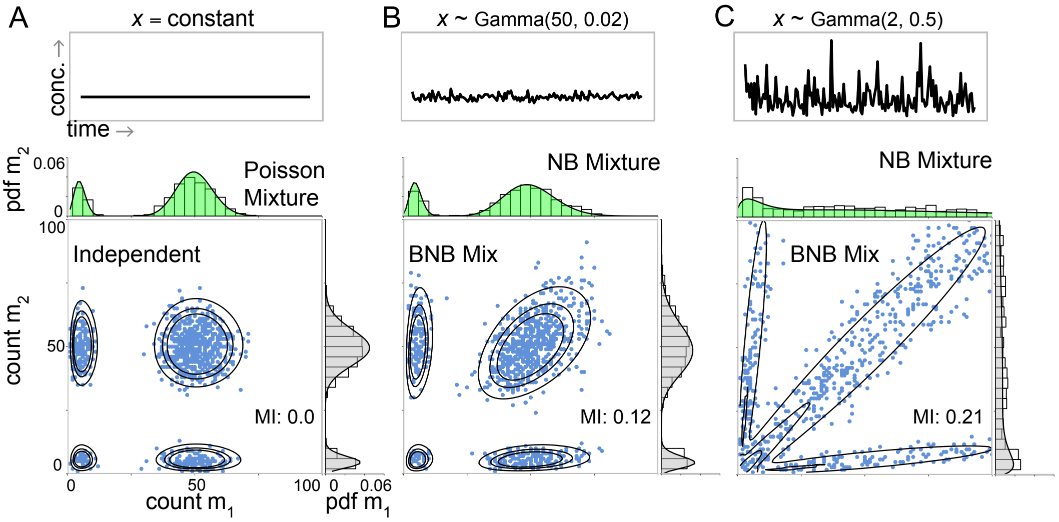

Supporting Figure 1: **Reporter accuracy depends upon regulatory context: 2-state genes.** Identical alleles of the same gene produce mRNA molecules M_1 and M_2. **Top panels**: Fluctuations of upstream regulator concentration, x. Panels show constant x (**A**) and $x \sim \text{Gamma}(r, \theta)$, for low regulator dispersion $\theta = 0.02$ (**B**) and high regulator dispersion $\theta = 0.5$ (**C**). **Bottom panels**: Joint and marginal distributions of m_1 and m_2 with upstream regulation given in top panel for two-state genes: Joint distribution given by the product of two Poisson mixtures (Eqn. (35)) or by bivariate BNB mixtures (Eqn. (40)), with $w = 0.8, \lambda_+ = 50$ and $\lambda_- = 5$ in all cases. w is the probability the gene is in the active state $(w_+/(w_+ + w_-))$ and λ_+, λ_- are the effective production rates in the 2 states. Marginal distributions are Poisson mixtures and negative binomial mixtures. For all joint distributions, contours show probabilities: 0.0001 inner, 0.0003 middle, 0.0005 outer. Scatter plots, histograms and mutual information (nats) are shown for a random sample of 1000 draws. The same scales apply to all comparable plots.

Figure S2

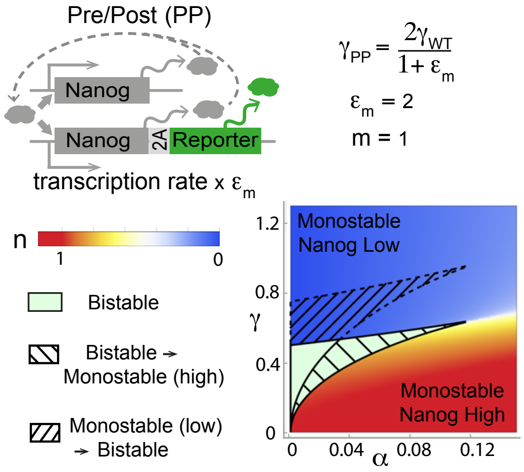

Supporting Figure 2: **Pre/Post (PP) Reporter with increased transcription rate.** The Pre/Post reporters can result in a change of transcription rate and this can either be a decrease (as shown in Figure 2) or an increase as shown in this case. When transcription rate increases by a factor of 2 ($\epsilon_m = 2$), γ_{pp} is reduced compared to γ_{wt}. Hatched areas indicate at risk regions of the parameter plane.

Figure S3

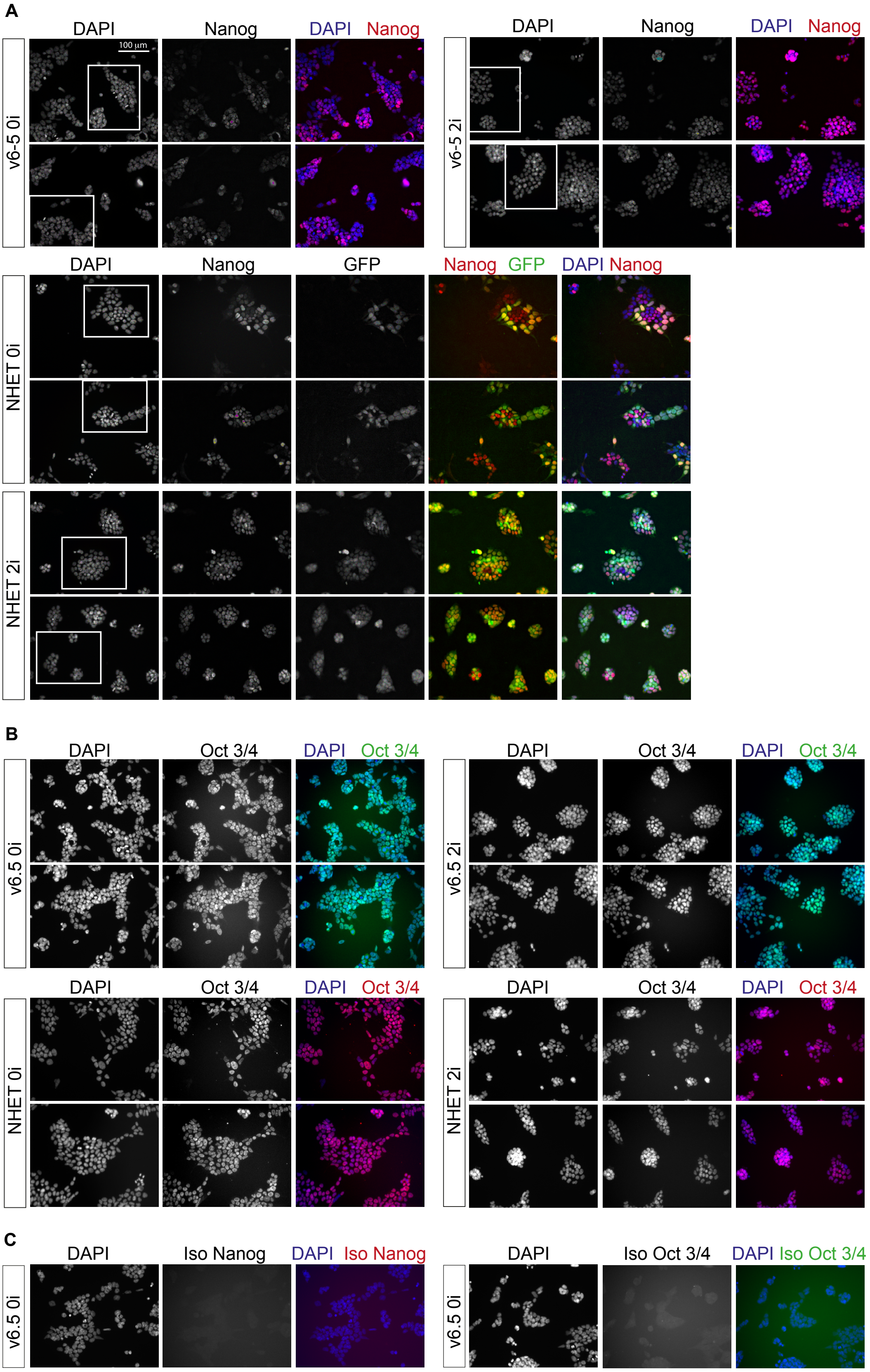

Supporting Figure 3: **Nanog and Oct3/4 immunofluorescence. A** Grayscale and composite RGB images of DAPI staining, Nanog immunofluorescence and GFP fluorescence in v6.5 and NHET cells in 0i and 2i conditions. Boxes indicate the regions of the image shown in Figure 1 main text. Variability of Nanog fluorescence can be seen for both v6.5 and NHET cells, with substantially greater variability in 0i conditions. **B** Grayscale and composite RGB images for Oct3/4 immunofluorescence from v6.5 and NHET cells in 0i and 2i cultures. Oct3/4 is less variably expressed than Nanog. **C** Grayscale and composite RGB images for Nanog and Oct3/4 antibody isotype controls for v6.5 0i cells.

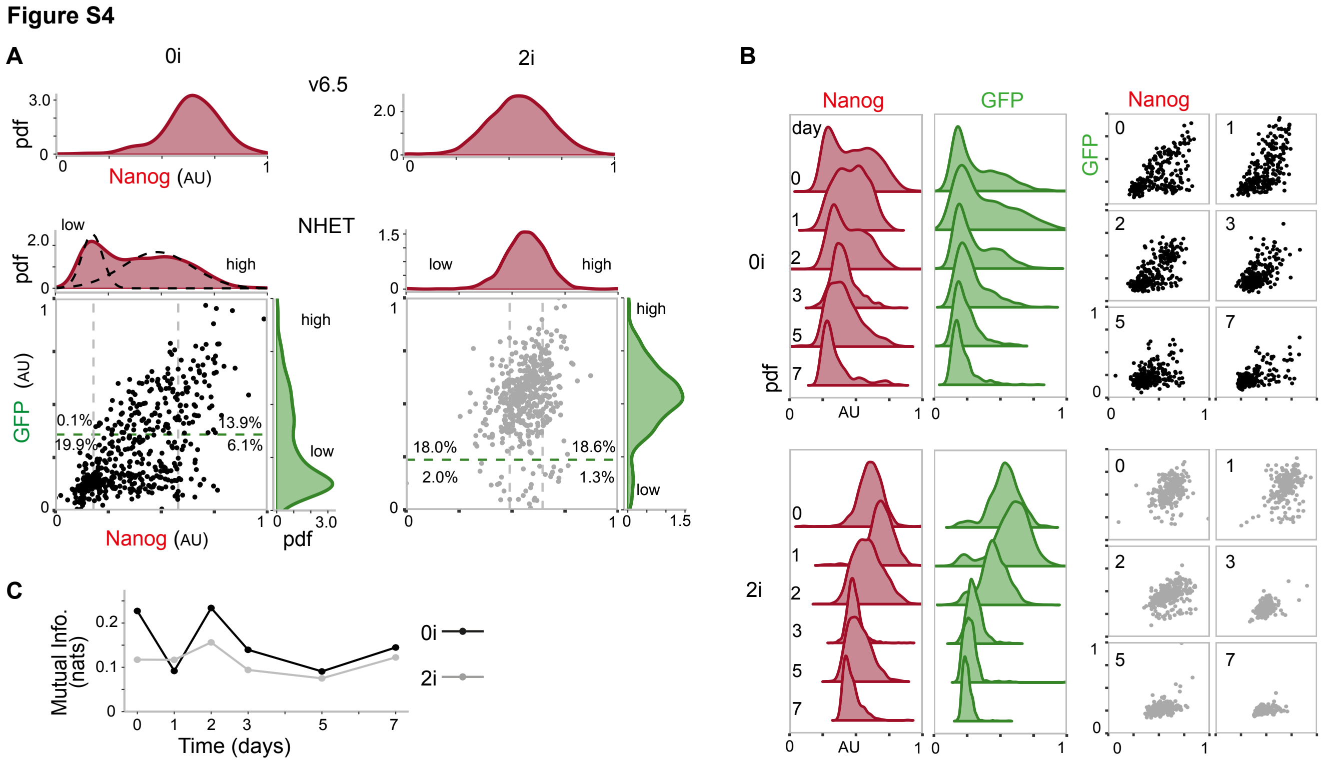

Supporting Figure 4: **Image analysis of Nanog variability in v6.5 and NHET cells. A** Examples of Nanog immunofluorescence distributions for v6.5 cells and joint Nanog-GFP distributions for NHET cells in 0i and 2i conditions, assessed by image analysis. NHET populations are split by GFP expression (high/low) and Nanog expression (highest 20% /lowest 20%). Percentages are shown for outer subpopulations. **B**. Assessment of joint Nanog-GFP distributions in NHET cells during differentiation subsequent to LIF-withdrawal starting from 0i and 2i cultures, using data assessed by image analysis. **C**. Mutual information between Nanog and GFP during differentiation, using data assessed by image analysis.
