# Supplementary material for: Nanog Fluctuations in Embryonic Stem Cells Highlight the Problem of Measurement in Cell Biology
Source: Biophys J. 2017 Jun 20;112(12):2641–52. doi: 10.1016/j.bpj.2017.05.005 (PMC5479053; doi:10.1016/j.bpj.2017.05.005)
Supplement: Document S2. Article plus Supporting Material [file mmc2.pdf]

Biophysical Journal
Article

Biophysical Society

Nanog Fluctuations in Embryonic Stem Cells Highlight the Problem of Measurement in Cell Biology

Rosanna C. G. Smith,[1] Patrick S. Stumpf,[1] Sonya J. Ridden,[2] Aaron Sim,[3] Sarah Filippi,[4,5] Heather A. Harrington,[6] and Ben D. MacArthur[1,2,7,*]

[1]Centre for Human Development, Stem Cells, and Regeneration, Faculty of Medicine and [2]Mathematical Sciences, University of Southampton, Southampton, United Kingdom; [3]Department of Life Sciences, [4]Department of Mathematics, and [5]Department of Epidemiology and Biostatistics, Imperial College London, London, United Kingdom; [6]Mathematical Institute, University of Oxford, Oxford, United Kingdom; and [7]Institute for Life Sciences, University of Southampton, Southampton, United Kingdom

ABSTRACT A number of important pluripotency regulators, including the transcription factor Nanog, are observed to fluctuate stochastically in individual embryonic stem cells. By transiently priming cells for commitment to different lineages, these fluctuations are thought to be important to the maintenance of, and exit from, pluripotency. However, because temporal changes in intracellular protein abundances cannot be measured directly in live cells, fluctuations are typically assessed using genetically engineered reporter cell lines that produce a fluorescent signal as a proxy for protein expression. Here, using a combination of mathematical modeling and experiment, we show that there are unforeseen ways in which widely used reporter strategies can systematically disturb the dynamics they are intended to monitor, sometimes giving profoundly misleading results. In the case of Nanog, we show how genetic reporters can compromise the behavior of important pluripotency-sustaining positive feedback loops, and induce a bifurcation in the underlying dynamics that gives rise to heterogeneous Nanog expression patterns in reporter cell lines that are not representative of the wild-type. These findings help explain the range of published observations of Nanog variability and highlight the problem of measurement in live cells.

INTRODUCTION

Fluorescence has been used to report expression of gene products in live cells since green fluorescent protein (GFP) was first cloned and utilized as a tracer (1,2). Live cell fluorescence imaging and analysis techniques allow investigation of temporal changes in protein expression and have consequently become an essential tool in modern molecular biology (3). However, their proper use requires the reporter signal to be representative of expression of the protein of interest at the scale of interest. In particular, if the reporter is to be used as a proxy for protein expression within a single cell, then, to be able to draw accurate conclusions, the reporter signal should be representative of protein expression in that particular cell. This issue is particularly relevant when functional assays are performed after cell sorting based upon reporter signal intensity, and can present a significant problem if the long-term outcome of any subsequent assays are driven by rare subpopulations of misidentified cells. As interest in single cell biology has increased, some generalized concerns about the fidelity of standard live-cell reporter strategies have been raised (4,5). However, the ways in which the genetic manipulations involved in generating reporter cell lines affect endogenous gene expression kinetics are not well understood.

Here, we explore how commonly used fluorescent reporter strategies can fail to accurately represent protein expression at the single cell level. For systems that use nonlinear feedback control mechanisms, we find that the introduction of reporter constructs can perturb important endogenous regulatory kinetics and induce qualitative changes in the protein expression patterns they are intended to measure. Because predicting when these problems will occur requires a priori knowledge of the underlying regulatory control mechanisms of the system under study—which is typically the knowledge that the reporter was introduced to provide—our results highlight a basic measurement problem in cell biology, reminiscent of that encountered in quantum physics (6,7), in which the act of measuring disturbs the

Submitted October 31, 2016, and accepted for publication May 5, 2017.

*Correspondence: bdm@soton.ac.uk

Editor: Jochen Guck.

http://dx.doi.org/10.1016/j.bpj.2017.05.005

© 2017 Biophysical Society.

This is an open access article under the CC BY license (http://creativecommons.org/licenses/by/4.0/).

CrossMark

system being measured. To illustrate these ideas, we consider the complications that can arise when using fluorescent reporters to monitor the expression dynamics of Nanog, a central element in the pluripotency regulatory network in mouse embryonic stem (ES) cells.

It has been widely observed that expression of a number of important pluripotency-associated transcription factors appears to fluctuate stochastically in individual ES cells (8–13). Although this heterogeneity has been linked to functional variability, its full developmental significance is still not well understood (14–16). The most widely studied of these fluctuating factors is Nanog, a core member of the regulatory network for pluripotency that is able to maintain pluripotency in vitro in the absence of Leukemia Inhibitory Factor (LIF), a cytokine normally required for the maintenance of self-renewal and prevention of differentiation (17–20).

Interest in Nanog expression variability began with a 2005 study by Hatano et al. (21) in which it was observed by direct immunostaining that mouse ES cell cultures displayed markedly heterogeneous patterns of Nanog protein expression, with a significant proportion of Oct4 positive pluripotent cells being negative for Nanog. A corresponding bimodal expression pattern was also observed via fluorescence in the first Nanog reporter ES cell lines (8,21). Subsequent studies indicated that Nanog levels appear to fluctuate stochastically in individual cells, thus providing a putative mechanism for the observed heterogeneity in expression (8,9,16,22). Importantly, during times of transient high Nanog expression, cells were observed to be resistant to differentiation cues; yet during times of transient low Nanog expression, cells became sensitive to differentiation-inducing stimuli. These results suggested that Nanog fluctuations are central to its role as molecular gatekeeper for pluripotency (8,9,23–26).

Many articles have followed up on this line of thought, and fluctuations in other factors have been investigated using similar strategies (10,11,27,28). Because these studies have focused on nuclear factors, they have generally employed fluorescence reporters to measure protein abundances, and it is typically implicitly assumed that the reporter signal is representative of expression of the factor of interest within individual cells, and, furthermore, that the observed patterns of expression in reporter cell lines are representative of those in (genetically unperturbed) wild-type cells. However, some concern has been raised that commonly used reporter strategies may not be representing Nanog expression patterns as accurately as they should. Faddah et al. (4) observed low correlation of reporter and Nanog mRNA levels in the original heterozygous reporter constructs; and both Faddah et al. (4) and Filipczyk et al. (5) have argued that observed heterogeneity of Nanog may be, at least in part, a reporter artifact.

To gain a more nuanced understanding of Nanog dynamics, more recent studies have employed a range of nonreporter (29,30) and live-imaging techniques (31–33), as well as more sophisticated protein and mRNA reporters (26,31,32). These methods include: construction of reporter constructs that use self-cleaving 2A peptide linkers and do not disrupt the Nanog coding region (4,19); fusion constructs in which Nanog is directly fused to a fluorescent protein (5,32); and bacterial artificial chromosome (BAC) transgenes, which carry a plasmid with the reporter gene under the Nanog promoter, leaving the wild-type Nanog alleles unchanged (22,26). Dual allele reporter systems have also been used to compare allele-specific Nanog abundances and assess total Nanog expression (4,19). Although self-cleaving, fusion, and BAC lines typically report more consistent Nanog expression patterns than the original heterozygous knock-in (21,34) and loss-of-function (8) reporter lines, there is still no consensus concerning the extent to which observed Nanog expression variability depends upon reporter type, cellular genetic background, and culture conditions.

Here, we address these issues using a combination of mathematical modeling and experiment. In the first part of the article we use a mathematical argument to show why it should not generally be expected that reporters will faithfully reflect gene expression dynamics at the single cell level, and why reporter accuracy depends strongly upon regulatory context. Surprisingly, this analysis also suggests that expression noise can improve, rather than degrade, reporter accuracy. To illustrate these general results, we then consider the case of Nanog, and find that a range of commonly used reporter strategies can alter the kinetics of endogenous Nanog regulatory control mechanisms and can induce a bifurcation in the underlying dynamics that gives rise to heterogeneous Nanog expression patterns in reporter lines that are not representative of the wild-type. We finish with a discussion of the general relevance of these results, and some suggestions for designing more effective reporters.

MATERIALS AND METHODS

Cell culture

Pluripotent mouse embryonic stem cells were cultivated in Dulbecco's Modified Eagle Medium with 1% Penicillin/Streptomycin, further supplemented with 10% fetal bovine serum, 1× Modified Eagle Medium nonessential amino acids, 1× GlutaMAX (GIBCO/Thermo Fisher Scientific, Waltham, MA), and 50 μM β-Mercaptoethanol. LIF was added at a dilution of 1:1000 (produced in-house). This is 0i culture medium. For 2i culture medium, 0i medium was supplemented with 1:10,000 10 mM PD0325901 (Cat. No. 4197; Tocris Bioscience, Bristol, UK) and 1:3000 10 mM CHIR99021 (Cat. No. 27-H76; Reagents Direct, Encinitas, CA). After transfer from 0i media, cells were adapted to 2i media over six passages. Cells were initially cultured on 0.1% gelatin-coated tissue culture plates pre-seeded with γ-irradiated mouse embryonic fibroblasts. After two passages, cells were cultivated on 0.1% gelatin-coated tissue culture plates without mouse embryonic fibroblasts. Cells were maintained at 37°C, 5% CO_2, routinely passaged every other day using Trypsin/EDTA detachment, and media was replaced every day. The wild-type male embryonic stem cell line v6.5 was purchased from Novus Biologicals (Cat. No. NBP1-41162; Littleton, CO). Nanog reporter cell line NHET was kindly provided by Jianlong Wang (Icahn School of Medicine, New York, NY). In this cell line,

originally generated by Maherali et al. (34) using the design of Hatano et al. (21), the endogenous Nanog open reading frame has been substituted by a gene cassette containing GFP in series with a Puromycin resistance casette, separated by an internal ribosome entry site (IRES). For 0i and 2i cultures, three technical replicates were assessed for Nanog and GFP distributions by flow cytometry and image analysis at passage number 11 (v6.5s) and passage number 20 (NHETs, also day 0 in time-course experiments). For undirected differentiation time-course experiments, three replicates from each initial condition (0i and 2i) were cultured separately for seven days after withdrawal of LIF from culture media on day 0. Cultures were passaged every two days and assessed by flow cytometry and image analysis on days 0, 1, 2, 3, 5, and 7.

Immunocytochemistry and flow cytometry

Cells for flow cytometry were detached using Trypsin/EDTA. Cells cultures for imaging were briefly washed in PBS. All cells were fixed for 20 min at room temperature (RT) in 4% Paraformaldehyde in PBS and washed three times with PBS. Cell and nuclear membranes were permeabilized using 0.1% Triton-X-100 in PBS for 10 min at RT. Unspecific antibody binding was blocked with 0.1% Triton-X-100 in PBS with 10% fetal bovine serum for 45 min at RT. Blocked cells were washed three times with blocking solution and resuspended in blocking solution containing either primary antibodies overnight at 4°C. Cell suspensions were under continuous agitation and cell plates were under continuous gentle motion. All experimental results in the main article used directly conjugated primary antibodies: Mouse anti-mouse Nanog (1:200, Cat. No. 560279, Alexa Fluor 647; Thermo Fisher Scientific, Waltham, MA), mouse IgG1κ isotype control (Cat. 557732, Alexa Fluor 647; Thermo Fisher Scientific), rat anti-histone H3 (pS28) (Cat. 560606, Alexa Fluor v450; Thermo Fisher Scientific), and rat IgG2a κ isotype control (Cat. No. 560377, Alexa Fluor v450; Thermo Fisher Scientific). Samples were washed three times with PBS and for cell imaging, nuclei were incubated with 20 μg/mL DAPI (Invitrogen, Carlsbad, CA) for 15 min before imaging. The following nonconjugated primary antibodies were also used: Mouse anti-Oct3/4 (c-10) (Cat. No. SC5279; Santa Cruz Biotechnology, Dallas, TX) and murine IgG2b isotype control (Cat. No. SAB4700729; Sigma-Aldrich, St. Louis, MO). After incubating with primary antibodies overnight, these samples were washed three times with blocking solution and incubated with secondary antibodies for 1 h at RT. Secondary antibodies: goat anti-mouse (IgG H&L) (Cat. No. abA11017, Alexa Fluor 488; Abcam, Abcam, Cambridge, UK) and goat anti-mouse IgG (Cat. No. 405322, Alexa Fluor 647; BioLegend, San Diego, CA). Images were recorded using an Eclipse Ti microscope (Nikon, Melville, NY). Cell suspension samples were analyzed using a BD FACS Aria II fluorescence activated cell sorting (FACS) device and BD FACSDIVA software (Becton-Dickinson, Oxford, UK). Flow cytometry analysis was performed using the softwares FlowJo (FlowJo LLC, Ashland, OR) and MATLAB (The MathWorks, Natick, MA), and the R programming language (35,36). Nanog and GFP fluorescence were quantified in terms of molecules of equivalent soluble fluorophore (MESF) units using Quantum Alexa Fluor 488 and 647 MESF calibration beads (Bangs Laboratories, Fishers, IN). Fluorescence probability distributions for nondirected differentiation experiments were aligned at the first percentile of Nanog and GFP observations between days.

Image analysis

Image analysis was carried out on grayscale fluorescence image sets using the software CellProfiler (http://cellprofiler.org/) (37). Each image set consisted of a 5×5 grid of adjacent images from a given cell culture. Nuclei were identified automatically based on DAPI signal and hand curated to exclude mitotic cells, unresolved or split nuclei, and those at the image edge. Spatially variable background fluorescence for each fluorescence channel was accounted for by determining the average illumination correction function per image set (38). The illumination correction function for each image was calculated by finding the minimum value pixel within a given block size (75 pixels) and then smoothed using a polynomial fit smoothing function. The average illumination correction function for each image set was subtracted from the Nanog and GFP signal images before measurement of the mean fluorescence intensity per nuclear area.

Model fitting

Nanog expression distributions from FACS and image analysis were fitted to a Gaussian mixture model with one or two components using expectation maximization. Model selection was conducted using Bayes information criterion. When fitting to two-component mixtures, to ensure that robustly bimodal distributions were identified, we required both components to have a weight >0.1 and excluded those models for which the peak probability density of one component was less than the probability density of the other component at the same point.

Mutual information calculation

Mutual information (MI) was estimated using the James-Stein-type shrinkage estimator (39). For MI between GFP and Nanog expression levels, discretization of each variable at each time point was performed separately via the Bayesian blocks method (40). Because MI is invariant to smooth reparameterization, we worked with the aligned, rescaled, log-transformed fluorescence values.

RESULTS

Regulatory noise and reporter accuracy

The first generation of Nanog studies used knock-in reporters to assess Nanog dynamics (8,21). Due to their simplicity, these and similar reporter designs are still widely used to assess expression fluctuations of Nanog and other key transcription factors in ES cells. In these constructs, one of the alleles for the gene of interest (for example, *Nanog*) is replaced with a reporter gene, often encoding for a fluorescent protein, perhaps with additional features such as an antibiotic selection cassette (3). Due to the loss of one gene copy, these are often described as heterozygous loss-of-function reporters. For such constructs to be effective at the single cell level, the fluorescence signal driven from the reporter allele should accurately represent protein expression from the wild-type allele. We therefore begin by considering a simple model of transcriptional coactivity, to explore the conditions under which two alleles that are subject to the same regulatory control may either synchronize or decouple in their activity, and thereby the conditions under which the output of one allele may be used to report on the other. For simplicity, we will focus on mRNA dynamics, but similar reasoning may also be extended to the protein level, and the general conclusions that we draw are not limited to heterozygous reporters (see Supporting Material and the following section for details). We will start with a simple model of a pair of constitutively active alleles before moving onto more realistic models of Nanog expression in the following sections.

Consider the transcriptional dynamics of two alleles of the same gene in a single cell. Let M_1 and M_2 denote the mRNA products of alleles 1 and 2, respectively; let $m_1(t)$ and $m_2(t)$ denote the number of mRNA transcripts in the cell associated with alleles 1 and 2, respectively, at time t; and assume that expression from both alleles is governed by linear birth-death processes with production rates ${k_b}^{(1)}$, ${k_b}^{(2)}$ and decay rates ${k_d}^{(1)}$, ${k_d}^{(2)}$. Thus, we are concerned with the dynamics of the following system of reactions:

$$\varnothing \underset{k_d^{(1)}}{\overset{k_b^{(1)}}{\rightleftarrows}} M_1 \qquad \varnothing \underset{k_d^{(2)}}{\overset{k_b^{(2)}}{\rightleftarrows}} M_2 \,. \tag{1}$$

This is clearly a simplistic view of transcription, yet it suffices to illustrate some of the essential issues regarding the reliability of reporters and is analytically tractable. Because the alleles are not coupled, they act independently and the stationary joint probability mass function (PMF) for this process is the product of two Poisson distributions (Fig.1 *A*), as follows:

$$p(m_1, m_2) = \frac{\lambda_1^{m_1}}{m_1!} e^{-\lambda_1} \times \frac{\lambda_2^{m_2}}{m_2!} e^{-\lambda_2}, \tag{2}$$

where $\lambda_i = k_b^{(i)}/k_d^{(i)}$ for $i = 1,2$. As we have not allowed for coregulation of expression, this model is rather artificial. In reality, we expect that if the alleles are both under the same promoter control then they will be regulated by the same upstream factors, and this coregulation may coordinate their dynamics. To couple the alleles, we allow the transcription rates to be driven by a shared upstream regulator X. Let x denote the concentration of X and let $\rho(x)$ be the stationary probability density function for x. Assuming that the mRNA birth rate is now given by $k_b^{(i)}x$, the stationary joint PMF is then obtained from Bayes' theorem, as follows:

$$\begin{aligned} p(m_1, m_2) &= \int_0^\infty p(m_1, m_2 \,|\, x)\rho(x)dx, \\ &= \int_0^\infty \frac{(\lambda_1 x)^{m_1}}{m_1!} e^{-\lambda_1 x} \times \frac{(\lambda_2 x)^{m_2}}{m_2!} e^{-\lambda_2 x} \rho(x)dx. \end{aligned} \tag{3}$$

Because the joint PMF $p(m_1,m_2)$ depends upon the distribution of the upstream regulator, an appropriate form for $\rho(x)$ must be chosen. It is commonly observed that protein concentrations are Gamma-distributed, so this is a natural choice (41). In the case $x \sim \text{Gamma}(r,\theta)$, and the joint PMF is as follows:

$$p(m_1, m_2) = \frac{\Gamma(m_1 + m_2 + r)}{m_1!\, m_2!\, \Gamma(r)} (1 - p - q)^r p^{m_1} q^{m_2}, \tag{4}$$

where $p = \lambda_1\theta/[1 + \theta(\lambda_1 + \lambda_2)]$, $q = ap$ with $a = \lambda_2 = \lambda_1$, and Γ is the Gamma function. The marginal distributions for the two allele products are then negative binomials (NB) and the joint PMF is a bivariate negative binomial distribution (BNB), as shown in Fig. 1, *B* and *C*. If the two alleles are kinetically identical ($\lambda_1 = \lambda_2$), then the marginal distributions will be identical, and the product of either allele may be used to report on the other at the population level assuming that the same dynamics occur within each cell in the population. However, this does not guarantee any

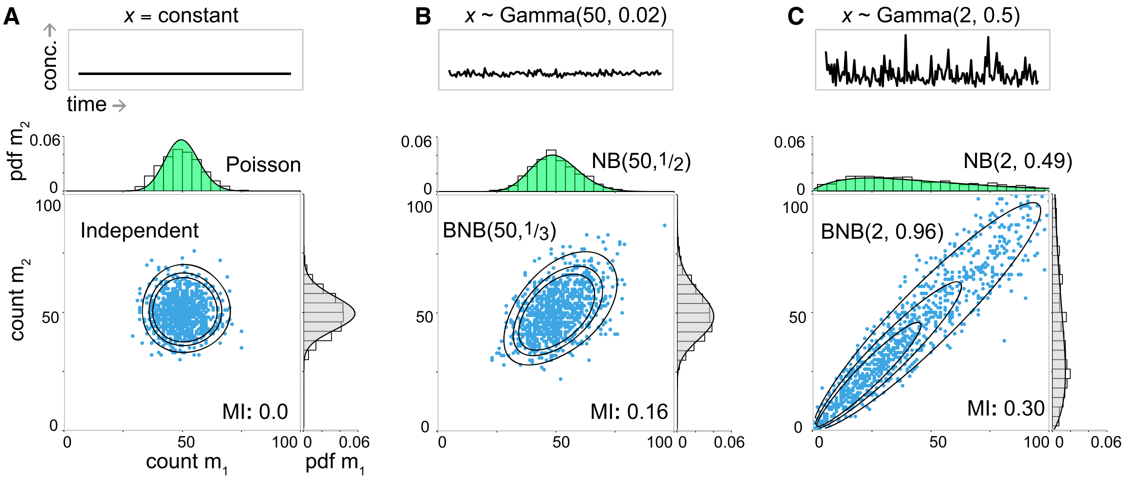

FIGURE 1 Reporter accuracy depends upon regulatory context. Identical alleles of the same gene produce mRNA molecules M_1 and M_2. Alleles 1 and 2 behave independently when there is no common upstream regulator or regulator concentration (x) is constant, yet become coupled if the upstream regulator fluctuates. (*Top panels*) Shown here are fluctuations of upstream regulator concentration. Panels show constant x (*A*) and $x \sim \text{Gamma}(r,\theta)$, for low regulator dispersion $\theta = 0.02$ (*B*) and high regulator dispersion $\theta = 0.5$ (*C*). (*Bottom panels*) Shown here are joint and marginal distributions for m_1 and m_2. Joint distributions are the product of two Poisson distributions (*A*, Eq. 2) and bivariate negative binomials, BNB(r,p), with $p = q$ for identical alleles (*B* and *C*; Eq. 4). All distributions use $\lambda = 50$ for both alleles and contours show probabilities: 0.0001 inner, 0.0003 middle, and 0.0005 outer. NB(r,p/(1$-p$)) denotes "negative binomial". Scatter plots, histograms, and MI (in nats) are shown for a random sample of 1000 draws. The same scales apply to all comparable plots. To see this figure in color, go online.

association between the allelic outputs at the individual cell level. To measure the degree of association between alleles within an individual cell, we must consider the covariance between their outputs, which is easily calculated in this case (see Supporting Material) and has a particularly simple form, given as follows:

$$\mathrm{Cov}(m_1, m_2) = \lambda_1 \lambda_2 \, \mathrm{Var}(x). \tag{5}$$

Thus, the covariance between the two allele products is proportional to the variance of the upstream regulator and the sensitivities of the two alleles to the upstream regulator. Whereas the form of joint PMF given in Eq. 4 depends upon the upstream regulator being Gamma-distributed, Eq. 5 holds for any upstream probability distribution $\rho(x)$, including, for example, the Gumbel distribution, which has been used to characterize extrinsic noise (42) (see Supporting Material). A comparable result may be obtained when transcription from each allele occurs at rate $k_b^{(i)}f(x)$, for any smooth function $f(x)$ (see Supporting Material). Similarly, it may also be shown that the correlation between the two alleles depends in a monotonic positive way on the Fano factor (or index of dispersion) of the upstream regulator (see Supporting Material).

Because the covariance between the two alleles is proportional to the variance of the upstream regulator, these results indicate that regulatory noise upstream can increase the coordination of alleles downstream and therefore improve reporter accuracy. Thus, although this model is the simplest possible to account for stochastic transcription from two alleles, it nevertheless provides insight into the effect of extrinsic fluctuations in a common upstream regulator on the coordination of two alleles. However, this model does not account for the fact that transcription from each allele typically occurs in bursts (43–46), and therefore cannot account for the effects of intrinsic noise on reporter accuracy.

A simple model of bursty transcription from a pair of alleles may be obtained by modifying this basic model to allow each allele to have two transcriptional states—one with a high rate of transcription, the other with a low rate of transcription—that they switch between stochastically at constant rates (45). In this case, the effective rates of transcription from each allele are now themselves stochastic processes and the mRNA coexpression dynamics are therefore described by a doubly stochastic process, which has accordingly more complex solutions (47). However, if the rate of switching between transcriptional states is slow with respect to mRNA degradation rate, then approximate solutions to this system may be obtained, and the covariance between alleles may again be found analytically (see Fig. S1 and Supporting Material for details). In particular, assuming that transcription from both alleles is driven by the same upstream regulator X and both alleles are kinetically identical, the covariance is as follows:

$$\mathrm{Cov}(m_1, m_2) = (w\lambda_+ + (1-w)\lambda_-)^2 \, \mathrm{Var}(x), \tag{6}$$

where w is the proportion time each allele spends in the active state, and $\lambda_+ + \lambda_-$ are the effective transcription rates from the active and inactive states, respectively. If $w = 1$, then then both alleles are constitutively active, and Eq. 6 reduces to Eq. 5 with $\lambda_+ = \lambda_1 = \lambda_2$. However, for $w < 1$ this equation highlights the different effects that intrinsic and extrinsic noise can have on allelic coordination. Because the covariance between alleles is again proportional to the variance of the upstream regulator, extrinsic fluctuations upstream always increase the covariance between alleles downstream and thereby improve reporter accuracy, as before. By contrast, because $w\lambda_+ + (1-w)\,\lambda_- < \lambda_+$, intrinsic noise due to transcriptional bursting always decreases the covariance between alleles and so always compromises reporter accuracy. These observations are in accordance with previous work in which dual and single allele reporters were used to assess the relative effects of intrinsic and extrinsic noise on gene expression (48–53).

Taken together, these results indicate that a reporter's accuracy depends on the regulatory context in which it is placed, here represented by the dynamics of the upstream regulator X, which are external to its design. In a complex regulatory environment, these external factors may not be fully (or even partially) known, and their effect on the reporter may be correspondingly hard to predict or control. Furthermore, because extrinsic regulatory factors are not usually constitutively expressed (as assumed in this basic model) but rather are themselves regulated by complex mechanisms—often involving feedback with the product of the gene being monitored—the dynamics of the regulatory environment may be intrinsically coupled to that of the target gene and its reporter(s). In this case, the insertion of a reporter construct may have unforeseen effects on endogenous regulatory kinetics, and change the dynamics of the system being studied in unpredictable ways. Because the ways in which such perturbations may arise will depend upon the particular details of system under study, it is helpful to consider a specific example. Here we examine the case of Nanog, an important transcriptional regulator of pluripotency in ES cells, which is known to be regulated by a complex network of direct and indirect feedback loops and for which different reporters have given different assessments of Nanog dynamics.

Reporter perturbation of Nanog dynamics

It has been widely observed that Nanog expression fluctuates stochastically in individual ES cells (8,9,11,26,29,32,33). However, different reporter constructs have given different assessments of the strength and developmental significance of these fluctuations (4,8,14–16,19,22,25) and some concerns have been raised that the use of reporters may be introducing artifacts that are confounding, rather than clarifying, our understanding of pluripotency (4,5). To address this issue, we

will consider a simple mathematical model of Nanog dynamics in ES cells in the presence of different kinds of reporter constructs. Nanog levels are regulated in pluripotent cells by a complex network of molecular interactions that involve both protein-protein and protein-DNA interactions (54–57). Given the complexity of this regulation, the fully stochastic framework used in the section above is not practical because all but the simplest stochastic processes are analytically intractable (58). So we instead use an ordinary differential equation approach. A number of groups have adopted a similar strategy when modeling these dynamics mathematically (9,59,60). At the core of this extended regulatory network is a series of nonlinear positive feedback loops that are dependent on Nanog for their function (24,61). Because these feedback loops are central to Nanog regulation, and to maintain a tractable mathematical model of general relevance, we will focus on this aspect of Nanog regulation here. Such positive feedback mechanisms naturally give rise to switchlike dynamics; they are correspondingly central to many kinds of cell fate decisions (62–64). Therefore, the model of positive feedback that we outline below is of general relevance to the design of reporters for other similarly regulated lineages specifying master transcription factors.

Mathematical model

We consider the following set of ordinary differential equations as a simple model of Nanog protein dynamics in wild-type cells:

$$\frac{dn_1}{dt} = c_b + \frac{c_f n^H}{K^H + n^H} - c_d n_1, \tag{7}$$

$$\frac{dn_2}{dt} = c_b + \frac{c_f n^H}{K^H + n^H} - c_d n_2, \tag{8}$$

where n_i denotes the concentration of the Nanog protein output of allele $i = 1,2$. The first terms on the right-hand sides of these equations account for baseline production at a constant rate c_b; the second terms are Hill functions that account for feedback-enhanced production at a rate dependent on total Nanog concentration $n = n_1 + n_2$, up to a maximum rate c_f; and the third terms account for Nanog protein decay at constant rate c_d. Hill functions are commonly used to model feedback processes, and may be derived as the effective production rate from a directly autoregulated two-state gene with stochastic transcriptional bursting (65), or as the result of more complex indirect feedback mechanisms (66). These equations therefore implicitly account for both direct Nanog autoregulation (61) and indirect feedback mechanisms in the core ES cell circuit (24,67) and thereby the effects of auxiliary factors such as other transcriptional regulators via their effect on the model rate constants, but for mathematical simplicity, the expression of these factors is not modeled explicitly. Adding these equations, we obtain an ordinary differential equation for the total Nanog protein concentration n, as follows:

$$\frac{dn}{dt} = 2c_b + 2\frac{c_f n^H}{K^H + n^H} - c_d n. \tag{9}$$

To better understand the model dynamics, it is convenient to nondimensionalize this equation. Doing so, using the scalings $n = 2c_f c_d^{-1}\overline{n}$, and $t = c_d^{-1}\tau$, we obtain the following:

$$\frac{d\overline{n}}{d\tau} = \alpha + \frac{\overline{n}^H}{\gamma^H + \overline{n}^H} - \overline{n}, \tag{10}$$

where $\overline{n}$ is the dimensionless total Nanog concentration and τ is dimensionless time. The dimensionless constants $\alpha = c_b/c_f$ and $\gamma = \gamma_{\mathrm{wt}} = c_d K/2c_f$ describe the relative strength of the basal and positive feedback enhanced production rates, respectively. Equation 10 has either one or two stable equilibrium solutions depending on the relative sizes of α, γ, and the Hill coefficient H. In particular, for $H > 1$, two bifurcation curves in the $\alpha\gamma$ plane may be found (see Supporting Material). The case $H = 2$ suffices to illustrate the general structure of the resulting classification diagram (see Fig. 2 *A*). In this case, the bifurcation curves are as follows:

$$\gamma_{\pm}(\alpha) = -\left(\alpha^2 - \frac{5}{2}\alpha - \frac{1}{8}\right) \pm \left(\frac{1}{4} - 2\alpha\right)^{\frac{3}{2}}. \tag{11}$$

If the model parameters fall inside the region enclosed by these curves, then Nanog expression dynamics are bistable; if the model parameters fall outside this region, then Nanog expression dynamics are monostable. In the presence of molecular noise, which is inherent to the intracellular microenvironment, bistability can give rise to coexisting subpopulations of phenotypically distinct cells within an isogenic population under the same environmental conditions (63). Thus, both homogeneous and heterogeneous Nanog expression patterns are allowed by this model, depending on whether the underlying dynamics are monostable or bistable. It should therefore be expected that Nanog expression patterns in ES cell populations will vary substantially under different experimental conditions, as is commonly observed (4,19,23), depending on how they stimulate Nanog feedback mechanisms. More significantly, it should also be expected that any genetic interventions that perturb the kinetics of Nanog feedback have the potential to push the dynamics in or out of the bistable regime, thereby affecting a qualitative change in expression patterns.

To see this, consider the case of a heterozygous knock-in reporter, in which one allele produces an inert reporter and one allele is left intact. In this case, the wild-type kinetics described by Eqs. 7 and 8 are modified as follows:

$$\frac{dn}{dt} = c_b + \frac{c_f n^H}{K^H + n^H} - c_d n, \tag{12}$$

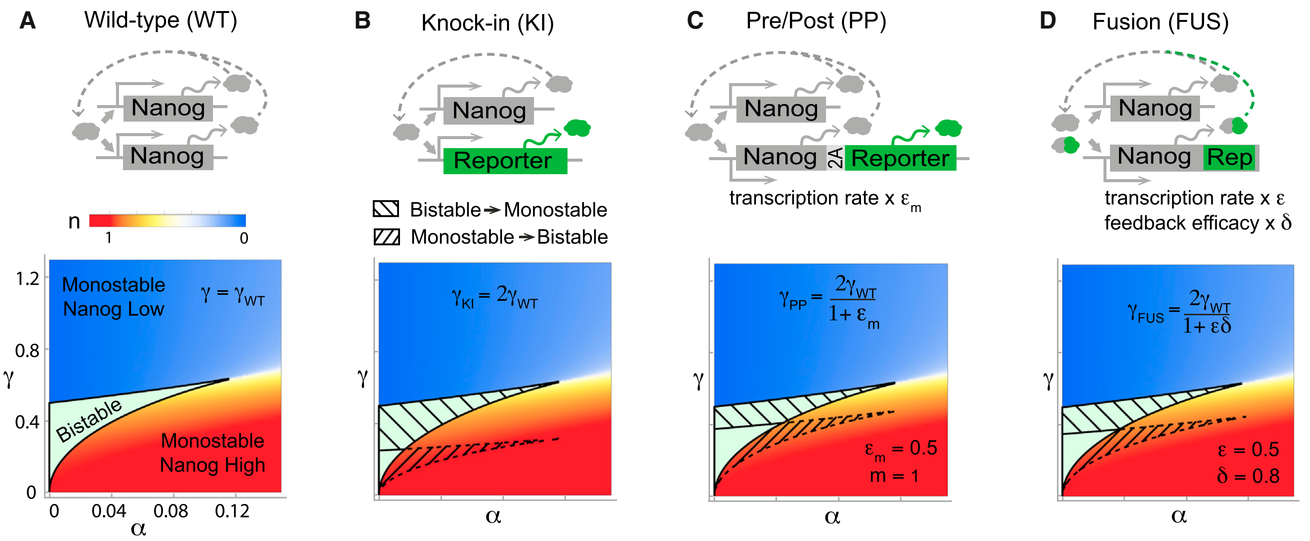

FIGURE 2 Perturbation of Nanog dynamics by reporters. (*A*) Given here is the wild-type: Nanog protein is produced from both alleles. Monostable or bistable dynamics can occur depending on α and γ. (*B*) Given here are knock-in reporters: one allele is left intact and one allele produces an inert reporter protein. Loss of one Nanog allele reduces Nanog production by a factor of 2, thereby doubling γ. (*C*) Given here are pre-/postreporters: both alleles encode for Nanog; *m* copies of a self-cleaving reporter protein are also transcribed from one allele. In the case shown, the transcription rate from the reporter allele is reduced by a factor $0 \leq \epsilon_m \leq 1$, thus increasing γ by a factor $2/(1 + \epsilon_m)$. If ϵ_m decreases with *m*, these reporters become more prone to systematic errors with each additional insert. (*D*) Given here are fusion reporters: one allele encodes for Nanog; the other for a fusion of Nanog and a reporter protein. Transcription rate from the reporter allele is altered by factor $0 \leq \epsilon_m \leq 1$ as for the PP reporter, but the reporter fusion also reduces Nanog feedback functionality by a factor $0 \leq \delta \leq 1$. Overall, this increases γ by a factor $2/(1 + \epsilon\delta)$. Hatching shows regions of the parameter plane at risk of qualitative changes in behavior when the reporter is introduced to the wild-type system. The upper-hatched regions are areas of parameter space for which the wild-type system is bistable, but the reporter system with the same underlying values of c_b, c_f, c_d, and K is monostable. The lower-hatched regions are areas of parameter space for which the wild-type system is monostable and the knock-in system is bistable. To see this figure in color, go online.

$$\frac{dr}{dt} = c_b + \frac{c_f n^H}{K^H + n^H} - c_d r, \tag{13}$$

where r is the reporter protein concentration. For simplicity we have assumed that the reporter and Nanog protein half-lives are perfectly matched. However, this assumption may be relaxed without altering conclusions qualitatively. Details of the dynamics when decay rates are mismatched are given in the Supporting Material. The dimensionless equation for total Nanog concentration in the reporter line is as follows:

$$\frac{d\overline{n}}{d\tau} = \alpha + \frac{\overline{n}^H}{\gamma_{\text{ki}}^H + \overline{n}^H} - \overline{n}, \tag{14}$$

where $\alpha = c_b/c_f$ as before, but $\gamma_{\text{ki}} = c_d K/c_f = 2\gamma_{\text{wt}}$ (see Supporting Material for details). In this case, the loss of Nanog production from one allele diminishes the Nanog production rate by a factor of 2, which weakens the endogenous feedback mechanisms and thereby doubles the parameter γ. Because for fixed α the magnitude of γ determines if the dynamics are monostable or bistable, and therefore if Nanog is homogeneously or heterogeneously expressed in the population, this change can induce a heterogeneous Nanog expression pattern in the reporter cell line that is not found in the wild-type (or vice versa). Areas in the $\alpha\gamma$ plane for which the map $(\alpha,\gamma) \mapsto (\alpha, 2\gamma)$ crosses one of the bifurcation curves in Eq. 11 are at risk of this kind of perturbation. Importantly, this problem is not restricted to knock-in lines: similar issues arise with a wide range of other reporters, in both single-allele and dual-allele reporter systems. Fig. 2 summarizes similar analyses for some other reporters. See Supporting Material for full details of calculations for these and a range of other reporter constructs.

For example, instead of replacing the Nanog protein coding region on one allele by that of a fluorescence reporter, the reporter construct may be inserted immediately pre/post the Nanog gene using a 2A self-cleaving peptide or internal ribosome entry site. If this insertion alters the Nanog mRNA transcription rate from that of the allele, then the resulting mismatch in transcription rates can lead to a perturbation similar to that of the knock-in. As there are many factors that influence transcription rate—including gene length, proximal pausing, and recruitment of RNA polymerase and cofactors (46,68)—it is reasonable to assume that insertion of a reporter construct has the potential to alter transcription rate through interference with one or more of these factors in a manner that is likely to be context-specific for each given gene. In this case, assuming that the transcription rate from the reporter allele is reduced by a factor of $0 \leq \epsilon \leq 1$, the insertion changes γ by a factor of $2/(1 + \epsilon)$ (see Fig. S2 and Supporting Material for full details). Thus, so long as the insertion does not completely

block transcription from the reporter allele (in which case, $\epsilon = 0$), pre-/postreporters are less likely than knock-in reporters to induce qualitative changes in Nanog expression dynamics, yet they are still subject to a similar type of systematic risk (compare the at-risk regions in Fig. 2, *B* and *C*). Furthermore, if multiple (m) reporters are inserted then this affect is compounded, assuming that the transcription rate from the reporter allele changes by a factor of $0 \leq \epsilon_m < \epsilon$. Thus, although multiple reporter additions may improve fluorescent signal, the risk of inducing a qualitative change in dynamics is increased with each additional reporter insert (see Supporting Material for more details). Similarly, fusion reporters, which encode a fusion of Nanog and a reporter protein from the reporting allele(s), are also susceptible to related problems. Assuming that the fusion reporter alters the transcription rate from the reporter allele by a factor of ϵ, and fusion of the reporter protein to Nanog reduces its functional efficacy by a factor $0 \leq \delta \leq 1$, fusion reporters change γ by a factor of $2/(1 + \epsilon\delta)$ (see Supporting Material for full details). In this case, the risk of a qualitative perturbation to the dynamics increases with both the extent to which the reporter perturbs transcription rate (ϵ) and the extent to which the attachment of the reporter protein to Nanog compromises Nanog function (δ).

Taken together, these theoretical considerations suggest that both technical and systematic errors can arise when using genetic reporters for Nanog. Technical errors occur due to the inevitable temporal mismatch between Nanog and reporter expression within individual cells, due, for example, to the effects of intrinsic noise on gene expression (as described in the previous section); systematic errors occur when unforeseen interactions between the reporter construct and the endogenous pluripotency regulatory circuitry induce qualitative changes in dynamics in the reporter cell line that are not representative of the wild-type (as described above).

Experimental results

To determine the extent to which these issues arise in experiment, we compared Nanog expression patterns in wild-type (male v6.5) mouse ES cells to those in a heterozygous knock-in reporter ES cell line with the same male v6.5 genetic background, in which the Nanog coding sequence was replaced with a GFP-IRES-puro reporter on one allele (34) (designated NHET cells). Cells were cultured in standard culture conditions (0i, serum plus LIF) and 2i conditions (0i conditions with the addition of mitogen-activated protein kinase and glycogen synthase kinase with three inhibitors), which maintain “ground state” pluripotency (69). Homogeneous Oct3/4 expression was confirmed via immunostaining in all culture conditions (Fig. S3). Nanog expression in individual cells was assessed via fluorescence immunolabeling and quantified by flow cytometry (Fig. 3) and image analysis (Fig. S4). Substantial variability in Nanog expression was observed in both v6.5 and NHET lines in 0i conditions (Fig. 3 *A*; Fig. S4). In accordance with previous reports, substantially less variability was observed in 2i conditions (Fig. 3 *A*; Fig. S4) (69). Distinctly bimodal GFP fluorescence was observed for NHET reporter cells in 0i cultures, with cell clusters containing both GFP high cells and GFP low cells present in abundance. In both conditions a clear mismatch between Nanog and GFP expression levels was observed in a substantial proportion of cells (Fig. 3 *B*). This was most apparent in 0i conditions, where, of the highest 20% of Nanog-expressing cells, 23% were GFP low and of the lowest 20% of Nanog-expressing cells, 11% were GFP high. In 2i conditions the percentage of GFP low cells was consistent across the Nanog distribution, suggesting that GFP status was not representative of Nanog expression. In addition, within the GFP high subset there was no clear association between Nanog and GFP expression levels (Fig. 3 *B*). Although 2i conditions showed more consistent Nanog-GFP coexpression patterns, there was a clear bias toward high GFP levels independently of Nanog expression (Fig. 3 *B*). These observations are indicative of technical errors due to expression noise (as described in the first section of this article) and we caution that some substantial contamination should be expected subsequent to cell sorting based on GFP signal as a proxy for Nanog when using such lines.

To determine whether Nanog expression was perturbed by introduction of the knock-in reporter, we compared Nanog distributions between NHET and wild-type v6.5 cell lines using immunostaining and flow cytometry. In NHET cells, the Nanog distribution in 0i conditions exhibited a wide, flattened distribution. Fitting of this data to a Gaussian mixture model with one or two components revealed that the two-component model best described the data, suggesting the presence of two coexisting subpopulations of cells characteristic of bistability in the underlying dynamics (Fig. 3 *B*). By contrast, in wild-type v6.5 cells, the Nanog distribution in 0i conditions was less broad and was better fit by a single-component model, suggesting monostability in the underlying dynamics (Fig. 3 *B*). To establish the robustness of these results, we also assessed Nanog expression using image analysis, and these broad conclusions were confirmed (Fig. S4 *A*). Taken together, these analyses suggest that the bimodal expression patterns observed in 0i conditions using the NHET line may be due to systematic perturbation of the Nanog regulatory network by the reporter, as predicted by theory. By contrast, both wild-type and NHET cells expressed similar, more compact, Nanog distributions in 2i conditions, with neither showing evidence of bimodality. This suggests that in 0i conditions, the wild-type system lies within the at-risk region of the $\alpha\gamma$ parameter plane, whereas in 2i conditions, the system lies outside the at-risk region.

To further investigate the extent to which environmental changes might affect the fidelity of the reporter output, we also sought to assess the association between Nanog and GFP during the process of cellular differentiation. Starting

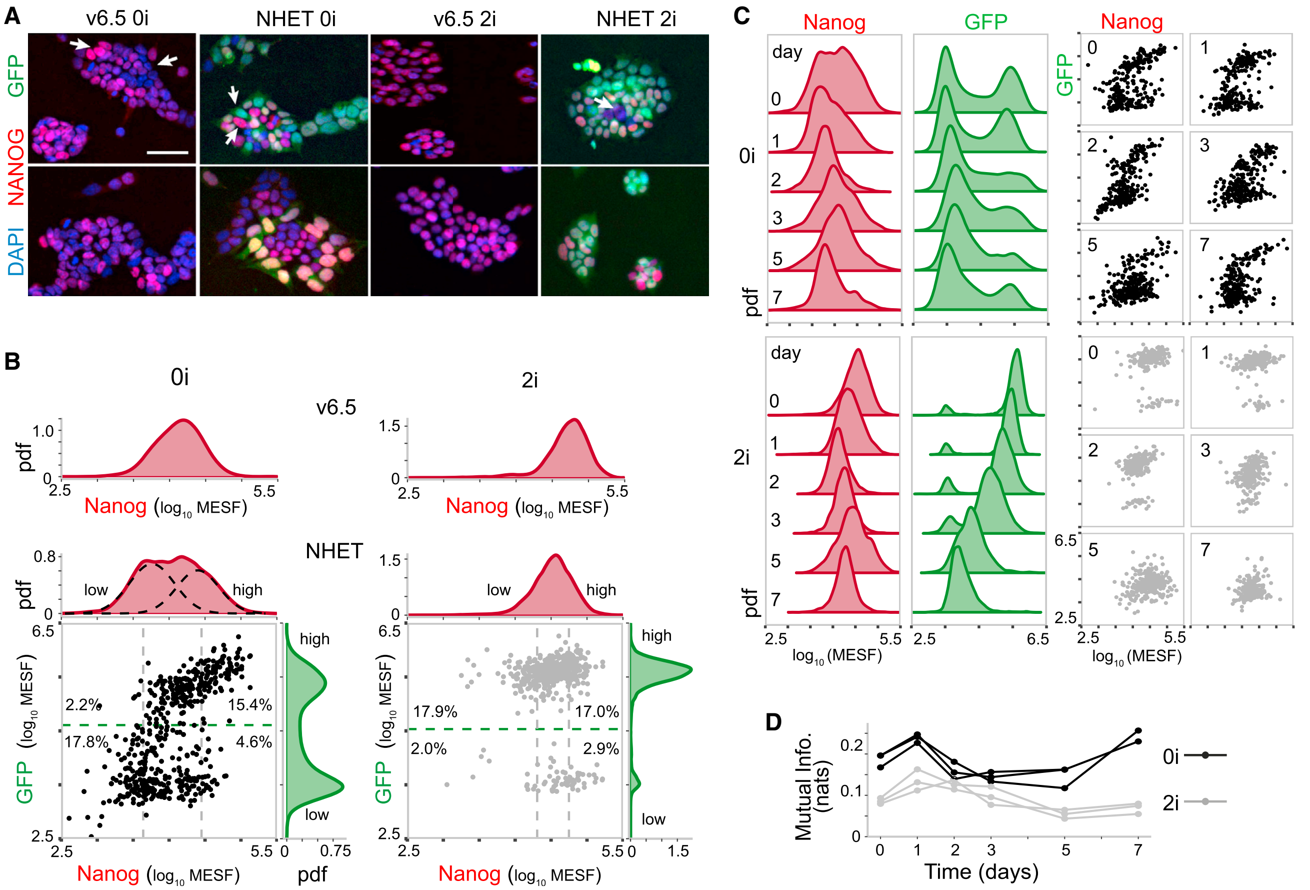

FIGURE 3 Nanog expression in wild-type and reporter cell lines. (*A*) Shown here are wild-type and Nanog reporter (NHET) cell cultures in 0i and 2i conditions. Nanog immunofluorescence is in red, and direct GFP fluorescence is in green. White arrows indicate Nanog low/high cells (wild-type) or cells in which there is a Nanog-GFP mismatch (NHET). Grayscale fluorescence signals are shown Fig. S3. Scale bar represents 50 μm. (*B*) Given here are representative flow cytometry distributions of Nanog in v6.5 wild-type cells (*top*) and Nanog-GFP joint distributions in NHET cells (*bottom*). Dashed-black lines show components of fit to a two-component Gaussian mixture model. Dashed-gray threshold lines indicate regions of Nanog high/low expression (highest 20% and lowest 20% of cells). Dashed-green lines indicate regions of GFP high/low expression (minimum between two peaks). Percentages show proportions of cells in the relevant subpopulations. (*C*) Shown here are changing Nanog and GFP distributions during undirected differentiation subsequent to LIF withdrawal starting from 0i (*top*) and 2i (*bottom*) cultures. Data from days 0, 1, 2, 3, 5, and 7 are shown. (*D*) Given here is MI between GFP and Nanog during differentiation. Results of three experimental repeats are shown. To see this figure in color, go online.

in 0i or 2i conditions, NHET cell cultures were allowed to undergo undirected differentiation by withdrawing LIF for a period of seven days. Fig. 3 *C* and Fig. S4 *B* show the evolving joint distributions for Nanog and GFP coexpression. From 0i conditions, the proportion of cells in the GFP high population gradually decreased over time and the corresponding Nanog distribution concomitantly evolved from an initial broad, flat distribution to a narrow distribution with lower average expression, indicating gradual loss of Nanog expression. From 2i conditions, both GFP and Nanog levels gradually declined over time without qualitative change in distribution shape. To quantify the association between Nanog and GFP levels, we calculated the mutual information (MI) between their expression patterns as follows:

$$I(n,r) = \int_0^\infty \int_0^\infty p(n,r)\log\left(\frac{p(n,r)}{p(n)p(r)}\right)dn\,dr, \qquad (15)$$

where $p(n,r)$ is the joint probability density function for Nanog and GFP coexpression, and $p(n)$ and $p(r)$ are the marginal probability density functions for Nanog and GFP expression, respectively. Mutual information is a powerful generalization of traditional measures of association, such as correlation, which is able to identify nonlinear relationships between variables (70). In this context, the MI provides an unbiased measure of the amount of information that knowledge of a cell's GFP status provides about its Nanog status (zero MI indicates complete independence; low values indicate near independence; high values indicate strong association). In all cases, the mutual information between Nanog and GFP exhibited a mild transient increase, indicating a slight increase in strength of association during the early stages of differentiation (Fig. 3 *D*; Fig. S4 *C*; compare the MI values in these plots with those in Fig. 1 for an informal assessment of their relative size). However,

mutual information was always low, indicating that Nanog and GFP signals are only weakly related both in and out of equilibrium (Fig. 3 *C*).

DISCUSSION

The advantages of genetic reporters are substantial: they provide a means to investigate expression dynamics of hard-to-monitor proteins and enable live cell observation, tracking, and selection. By assessing expression directly via fluorescence rather than indirectly via immunolabeling they also provide a more transparent way to assess protein activity, free of the reproducibility issues associated with the use of antibodies. However, it is generally accepted that genetic reporter systems are not perfect: quantification is normally relative, reporter fluorescence is an imperfect proxy-measurement for the variable of real interest, and it is known that wild-type dynamics may be compromised, for example by fusing cumbersome fluorescent proteins to (often relatively small) proteins of interest (71). To assess the importance of these issues, the advantages and disadvantages of different types of reporter are usually considered purely in terms of their technical characteristics, or with only limited concern for their regulatory context, for instance to match reporter half-life to that of the protein of interest (72). Here, we have explored how intrinsic and extrinsic noise and reporter interactions with endogenous regulatory mechanisms affect reporter accuracy, focusing on Nanog as an example. Although technical issues relating to noise and reporter protein mismatch are generally well accepted, the systematic limitations we have identified, have not been well appreciated. Yet, our results show that if such limitations are not taken into account then confounding results can follow. The example of Nanog shows how a whole field of study can become complicated by these issues.

Taken together, this work suggests several practical guidelines to help prevent unforeseen issues with reporter observations: first, the scale at which the reporter is used should be considered. In particular, for assays involving cell sorting based upon reporter signal, accuracy should be tested at the single cell level before subsequent functional assays. Second, systematic limitations of reporters, due to interactions between the reporter and its regulatory context, should also be considered. The most appropriate reporter strategy will be determined by a trade-off between the type of spatial and temporal information required, the strength of the reporter signal required, and the likelihood that the reporter chosen will qualitatively perturb the endogenous kinetics. For example, reporters that produce multiple copies of a fluorescent protein per copy of the protein of interest naturally produce a stronger fluorescence signal, yet their construction involves greater genetic intervention, so they also carry a correspondingly higher systematic risk (see Fig. 2; Supporting Material). Before designing or using such reporters the benefits of increased signal should therefore be weighed against the increased possibility of systematic errors. For genes that are regulated by positive feedback mechanisms—which includes many developmentally important factors (24,59,62,64)—the risk of systematic failures is greatest for knock-in reporters and least for BAC reporters, and single allele reporters carry less systematic risk than dual allele reporters (see Supporting Material).

Because systematic perturbations depend on the details of the regulatory kinetics of particular system under study, it is difficult to determine a priori when they will occur. One potential strategy is to engineer two separate reporter cell lines for the same factor: one in which expression of the gene of interest is monitored in one color, and expression of an inert downstream target of the gene of interest (which does not affect the dynamics of the upstream regulator either directly or indirectly) is monitored in a different color; and a second in which only the downstream gene is monitored and the gene of interest is left unperturbed. Potential systematic perturbations to the dynamics of the upstream gene may then be identified by careful comparison of the reporter distributions for the downstream target in the two reporter cell lines. In all cases, because reporter accuracy depends intimately on regulatory context, and the same reporter in the same cells may fail in some experimental conditions and succeed in others, quality controls should be conducted for all experimental conditions under consideration.

SUPPORTING MATERIAL

Supporting Materials and Methods, four figures, and two tables are available at http://www.biophysj.org/biophysj/supplemental/S0006-3495(17)30509-X.

AUTHOR CONTRIBUTIONS

R.C.G.S. and B.D.M. wrote the article. R.C.G.S. and P.S.S. did the experiments. R.C.G.S., P.S.S., S.J.R., and B.D.M. did the mathematical modeling. R.C.G.S., A.S., S.F., and H.A.H. analyzed the experimental data. R.C.G.S. and B.D.M. designed the study.

ACKNOWLEDGMENTS

We thank Neil Smyth for the provision of LIF and Jianlong Wang for the NHET cell line.

This work was funded by Biotechnology and Biological Sciences Research Council (BBSRC) grant No. BB/L000512/1 and Engineering and Physical Sciences Research Council (EPSRC) grant No. EP/K041096/1. H.A.H. acknowledges a Royal Society University Research Fellowship.

REFERENCES

1. Prasher, D. C., V. K. Eckenrode, …, M. J. Cormier. 1992. Primary structure of the *Aequorea victoria* green-fluorescent protein. *Gene.* 111:229–233.

2. Chalfie, M., Y. Tu, …, D. C. Prasher. 1994. Green fluorescent protein as a marker for gene expression. *Science.* 263:802–805.

3. Chalfie, M., and S. R. Kain. 2005. Green Fluorescent Protein: Properties, Applications and Protocols, 2nd Ed. John Wiley and Sons, Edison, NJ.

4. Faddah, D. A., H. Wang, …, R. Jaenisch. 2013. Single-cell analysis reveals that expression of Nanog is biallelic and equally variable as that of other pluripotency factors in mouse ESCs. *Cell Stem Cell.* 13:23–29.

5. Filipczyk, A., K. Gkatzis, …, T. Schroeder. 2013. Biallelic expression of Nanog protein in mouse embryonic stem cells. *Cell Stem Cell.* 13:12–13.

6. Wigner, E. 1963. The problem of measurement. *Am. J. Phys.* 31:6–15.

7. Potten, C. S., and M. Loeffler. 1990. Stem cells: attributes, cycles, spirals, pitfalls and uncertainties. Lessons for and from the crypt. *Development.* 110:1001–1020.

8. Chambers, I., J. Silva, …, A. Smith. 2007. Nanog safeguards pluripotency and mediates germline development. *Nature.* 450:1230–1234.

9. Kalmar, T., C. Lim, …, A. Martinez Arias. 2009. Regulated fluctuations in Nanog expression mediate cell fate decisions in embryonic stem cells. *PLoS Biol.* 7:e1000149.

10. Hayashi, K., S. M. C. S. Lopes, …, M. A. Surani. 2008. Dynamic equilibrium and heterogeneity of mouse pluripotent stem cells with distinct functional and epigenetic states. *Cell Stem Cell.* 3:391–401.

11. Canham, M. A., A. A. Sharov, …, J. M. Brickman. 2010. Functional heterogeneity of embryonic stem cells revealed through translational amplification of an early endodermal transcript. *PLoS Biol.* 8:e1000379.

12. Trott, J., K. Hayashi, …, A. Martinez-Arias. 2012. Dissecting ensemble networks in ES cell populations reveals micro-heterogeneity underlying pluripotency. *Mol. Biosyst.* 8:744–752.

13. Kumar, R. M., P. Cahan, …, J. J. Collins. 2014. Deconstructing transcriptional heterogeneity in pluripotent stem cells. *Nature.* 516:56–61.

14. Martinez Arias, A., and J. M. Brickman. 2011. Gene expression heterogeneities in embryonic stem cell populations: origin and function. *Curr. Opin. Cell Biol.* 23:650–656.

15. Cahan, P., and G. Q. Daley. 2013. Origins and implications of pluripotent stem cell variability and heterogeneity. *Nat. Rev. Mol. Cell Biol.* 14:357–368.

16. Torres-Padilla, M.-E., and I. Chambers. 2014. Transcription factor heterogeneity in pluripotent stem cells: a stochastic advantage. *Development.* 141:2173–2181.

17. Chambers, I., D. Colby, …, A. Smith. 2003. Functional expression cloning of Nanog, a pluripotency sustaining factor in embryonic stem cells. *Cell.* 113:643–655.

18. Mitsui, K., Y. Tokuzawa, …, S. Yamanaka. 2003. The homeoprotein Nanog is required for maintenance of pluripotency in mouse epiblast and ES cells. *Cell.* 113:631–642.

19. Miyanari, Y., and M.-E. Torres-Padilla. 2012. Control of ground-state pluripotency by allelic regulation of Nanog. *Nature.* 483:470–473.

20. Saunders, A., F. Faiola, and J. Wang. 2013. Concise review: pursuing self-renewal and pluripotency with the stem cell factor Nanog. *Stem Cells.* 31:1227–1236.

21. Hatano, S.-Y., M. Tada, …, T. Tada. 2005. Pluripotential competence of cells associated with Nanog activity. *Mech. Dev.* 122:67–79.

22. Abranches, E., E. Bekman, and D. Henrique. 2013. Generation and characterization of a novel mouse embryonic stem cell line with a dynamic reporter of Nanog expression. *PLoS One.* 8:e59928.

23. Silva, J., J. Nichols, …, A. Smith. 2009. Nanog is the gateway to the pluripotent ground state. *Cell.* 138:722–737.

24. MacArthur, B. D., A. Sevilla, …, I. R. Lemischka. 2012. Nanog-dependent feedback loops regulate murine embryonic stem cell heterogeneity. *Nat. Cell Biol.* 14:1139–1147.

25. Abranches, E., A. M. V. Guedes, …, D. Henrique. 2014. Stochastic NANOG fluctuations allow mouse embryonic stem cells to explore pluripotency. *Development.* 141:2770–2779.

26. Xenopoulos, P., M. Kang, …, A.-K. Hadjantonakis. 2015. Heterogeneities in Nanog expression drive stable commitment to pluripotency in the mouse blastocyst. *Cell Reports.* 10:1508–1520.

27. Toyooka, Y., D. Shimosato, …, H. Niwa. 2008. Identification and characterization of subpopulations in undifferentiated ES cell culture. *Development.* 135:909–918.

28. Kobayashi, T., H. Mizuno, …, R. Kageyama. 2009. The cyclic gene Hes1 contributes to diverse differentiation responses of embryonic stem cells. *Genes Dev.* 23:1870–1875.

29. Singer, Z. S., J. Yong, …, M. B. Elowitz. 2014. Dynamic heterogeneity and DNA methylation in embryonic stem cells. *Mol. Cell.* 55:319–331.

30. Skinner, S. O., H. Xu, …, I. Golding. 2016. Single-cell analysis of transcription kinetics across the cell cycle. *eLife.* 5:e12175.

31. Ochiai, H., T. Sugawara, …, T. Yamamoto. 2014. Stochastic promoter activation affects Nanog expression variability in mouse embryonic stem cells. *Sci. Rep.* 4:7125.

32. Filipczyk, A., C. Marr, …, T. Schroeder. 2015. Network plasticity of pluripotency transcription factors in embryonic stem cells. *Nat. Cell Biol.* 17:1235–1246.

33. Cannon, D., A. M. Corrigan, …, J. R. Chubb. 2015. Multiple cell and population-level interactions with mouse embryonic stem cell heterogeneity. *Development.* 142:2840–2849.

34. Maherali, N., R. Sridharan, …, K. Hochedlinger. 2007. Directly reprogrammed fibroblasts show global epigenetic remodeling and widespread tissue contribution. *Cell Stem Cell.* 1:55–70.

35. R Core Team. 2016. R: A Language and Environment for Statistical Computing. R Foundation for Statistical Computing, Vienna, Austria https://www.R-project.org/.

36. Wickham, H. 2009. ggplot2: Elegant Graphics for Data Analysis. Springer, New York http://ggplot2.org.

37. Kamentsky, L., T. R. Jones, …, A. E. Carpenter. 2011. Improved structure, function and compatibility for CellProfiler: modular high-throughput image analysis software. *Bioinformatics.* 27:1179–1180.

38. Bray, M. A., M. S. Vokes, and A. E. Carpenter. 2015. Using CellProfiler for automatic identification and measurement of biological objects in images. *Curr. Proto. Mol. Biol.* 109:14.17.1–14.17.13.

39. Hausser, J., and K. Strimmer. 2009. Entropy inference and the James-Stein estimator, with application to nonlinear gene association networks. *J. Mach. Learn. Res.* 10:1469–1484.

40. Scargle, J. D. 1998. Studies in astronomical time series analysis. V. Bayesian blocks, a new method to analyze structure in photon counting data. *Astrophys. J.* 504:405–418.

41. Friedman, N., L. Cai, and X. S. Xie. 2006. Linking stochastic dynamics to population distribution: an analytical framework of gene expression. *Phys. Rev. Lett.* 97:168302.

42. Sherman, M. S., K. Lorenz, …, B. A. Cohen. 2015. Cell-to-cell variability in the propensity to transcribe explains correlated fluctuations in gene expression. *Cell Syst.* 1:315–325.

43. Golding, I., J. Paulsson, …, E. C. Cox. 2005. Real-time kinetics of gene activity in individual bacteria. *Cell.* 123:1025–1036.

44. Chubb, J. R., T. Trcek, …, R. H. Singer. 2006. Transcriptional pulsing of a developmental gene. *Curr. Biol.* 16:1018–1025.

45. Raj, A., C. S. Peskin, …, S. Tyagi. 2006. Stochastic mRNA synthesis in mammalian cells. *PLoS Biol.* 4:e309.

46. Lenstra, T. L., J. Rodriguez, …, D. R. Larson. 2016. Transcription dynamics in living cells. *Annu. Rev. Biophys.* 45:25–47.

47. Iyer-Biswas, S., F. Hayot, and C. Jayaprakash. 2009. Stochasticity of gene products from transcriptional pulsing. *Phys. Rev. E Stat. Nonlin. Soft Matter Phys.* 79:031911.

48. Elowitz, M. B., A. J. Levine, …, P. S. Swain. 2002. Stochastic gene expression in a single cell. *Science.* 297:1183–1186.

49. Swain, P. S., M. B. Elowitz, and E. D. Siggia. 2002. Intrinsic and extrinsic contributions to stochasticity in gene expression. *Proc. Natl. Acad. Sci. USA.* 99:12795–12800.

50. Paulsson, J. 2004. Summing up the noise in gene networks. *Nature.* 427:415–418.

51. Kaern, M., T. C. Elston, …, J. J. Collins. 2005. Stochasticity in gene expression: from theories to phenotypes. *Nat. Rev. Genet.* 6:451–464.

52. Raj, A., and A. van Oudenaarden. 2008. Nature, nurture, or chance: stochastic gene expression and its consequences. *Cell.* 135:216–226.

53. Li, G.-W., and X. Sunney Xie. 2011. NIH Public Access. *Nat.* 475: 308–315.

54. Wang, J., S. Rao, …, S. H. Orkin. 2006. A protein interaction network for pluripotency of embryonic stem cells. *Nature.* 444:364–368.

55. Kim, J., J. Chu, …, S. H. Orkin. 2008. An extended transcriptional network for pluripotency of embryonic stem cells. *Cell.* 132:1049–1061.

56. Macarthur, B. D., A. Ma'ayan, and I. R. Lemischka. 2009. Systems biology of stem cell fate and cellular reprogramming. *Nat. Rev. Mol. Cell Biol.* 10:672–681.

57. Dunn, S.-J., G. Martello, …, A. G. Smith. 2014. Defining an essential transcription factor program for naïve pluripotency. *Science.* 344: 1156–1160.

58. van Kampen, N. G. 2007. Stochastic Processes in Physics and Chemistry, 3rd Ed. North-Holland, Amsterdam, the Netherlands.

59. MacArthur, B. D., C. P. Please, and R. O. C. Oreffo. 2008. Stochasticity and the molecular mechanisms of induced pluripotency. *PLoS One.* 3:e3086.

60. Glauche, I., M. Herberg, and I. Roeder. 2010. Nanog variability and pluripotency regulation of embryonic stem cells—insights from a mathematical model analysis. *PLoS One.* 5:e11238.

61. Wang, J., D. N. Levasseur, and S. H. Orkin. 2008. Requirement of Nanog dimerization for stem cell self-renewal and pluripotency. *Proc. Natl. Acad. Sci. USA.* 105:6326–6331.

62. Xiong, W., and J. E. J. Ferrell, Jr. 2003. A positive-feedback-based bistable 'memory module' that governs a cell fate decision. *Nature.* 426:460–465.

63. Tyson, J. J., K. C. Chen, and B. Novak. 2003. Sniffers, buzzers, toggles and blinkers: dynamics of regulatory and signaling pathways in the cell. *Curr. Opin. Cell Biol.* 15:221–231.

64. Becskei, A., B. Séraphin, and L. Serrano. 2001. Positive feedback in eukaryotic gene networks: cell differentiation by graded to binary response conversion. *EMBO J.* 20:2528–2535.

65. Walczak, A., A. Mugler, and C. H. Wiggins. 2012. Analytic methods for modeling stochastic regulatory networks. *In* Computational Modeling of Signaling Networks. Humana Press, Totowa, NJ, pp. 273–322.

66. Alon, U. 2007. An Introduction to Systems Biology: Design Principles of Biological Circuits. Chapman and Hall, London, UK.

67. Andrecut, M., J. D. Halley, …, S. Huang. 2011. A general model for binary cell fate decision gene circuits with degeneracy: indeterminacy and switch behavior in the absence of cooperativity. *PLoS One.* 6:e19358.

68. Jonkers, I., and J. T. Lis. 2015. Getting up to speed with transcription elongation by RNA polymerase II. *Nat. Rev. Mol. Cell Biol.* 16: 167–177.

69. Ying, Q.-L., J. Wray, …, A. Smith. 2008. The ground state of embryonic stem cell self-renewal. *Nature.* 453:519–523.

70. Cover, T. M., and J. A. Thomas. 2006. Elements of Information Theory, 2nd Ed. John Wiley and Sons, Edison, NJ.

71. Snapp, E. 2005. Design and use of fluorescent fusion proteins in cell biology. *Curr. Protoc. Cell Biol.* chapter 21 Unit 21.4. http://dx.doi.org/10.1002/0471143030.cb2104s27.

72. Day, R. N., and M. W. Davidson. 2009. The fluorescent protein palette: tools for cellular imaging. *Chem. Soc. Rev.* 38:2887–2921.

Biophysical Journal, Volume *112*

Supplemental Information

Nanog Fluctuations in Embryonic Stem Cells Highlight the Problem of Measurement in Cell Biology

Rosanna C.G. Smith, Patrick S. Stumpf, Sonya J. Ridden, Aaron Sim, Sarah Filippi, Heather A. Harrington, and Ben D. MacArthur

Nanog Fluctuations in Embryonic Stem Cells Highlight the Problem of Measurement in Cell Biology

Supporting Material

Rosanna C G Smith[1], Patrick S Stumpf[1], Sonya J Ridden[2], Aaron Sim[3], Sarah Filippi[4], Heather A Harrington[5], and Ben D MacArthur[1,2,6,*]

[1]Centre for Human Development, Stem Cells and Regeneration, Faculty of Medicine, University of Southampton, SO17 1BJ, UK.
[2]Mathematical Sciences, University of Southampton, SO17 1BJ, UK.
[3]Department of Life Sciences, Imperial College London, SW7 2AZ, UK.
[4]Department of Mathematics, Department of Epidemiology and Biostatistics, Imperial College London, SW7 2AZ, UK.
[5]Mathematical Institute, University of Oxford, OX2 6GG, UK.
[6]Institute for Life Sciences, University of Southampton, SO17 1BJ, UK.
[*]To whom correspondence should be addressed: bdm@soton.ac.uk

Contents

1 Mathematical details **1**
1.1 Allelic synchronization and mRNA co-expression dynamics . . . 1
1.1.1 Two single-state alleles with upstream regulation . . . 1
1.1.2 One two-state allele with upstream regulation . . . 3
1.1.3 Two two-state alleles with upstream regulation . . . 5
1.2 Bifurcation curves for Nanog dynamics . . . 7
1.3 Reporter perturbations . . . 8
1.4 Single allele reporter strategies . . . 8
1.4.1 Knock-in reporters . . . 8
1.4.2 Pre/post (PP) reporters . . . 9
1.4.3 Multiple pre/post (MPP) reporters . . . 9
1.4.4 Fusion reporters . . . 10
1.4.5 BAC reporters . . . 10
1.5 Dual allele reporter strategies . . . 10
1.6 Decay constant mismatch . . . 11

2 Supporting Tables and Figures **11**

1 Mathematical details

1.1 Allelic synchronization and mRNA co-expression dynamics

1.1.1 Two single-state alleles with upstream regulation

Consider the transcriptional dynamics of 2 alleles of the same gene in a single cell. Let M_1 denote the mRNA transcript associated with allele 1, let M_2 denote the mRNA transcripts associated with allele 2, and assume that expression of both alleles are governed by linear birth-death processes with production rates $k_b^{(1)}$, $k_b^{(2)}$ and decay rates $k_d^{(1)}$, $k_d^{(2)}$. Thus, we are concerned with the dynamics of the following system of reactions:

$$\emptyset \underset{k_d^{(1)}}{\overset{k_b^{(1)}}{\rightleftarrows}} M_1 \qquad \emptyset \underset{k_d^{(2)}}{\overset{k_b^{(2)}}{\rightleftarrows}} M_2. \tag{1}$$

The number of species M_1 and M_2 are given by m_1 and m_2 respectively. Since the alleles are not coupled together they act independently and the stationary joint probability mass function (PMF) for this process is a prodcut of two independent Poisson processes:

$$p(m_1, m_2) = \frac{\lambda_1^{m_1}}{m_1!}\mathrm{e}^{-\lambda_1} \cdot \frac{\lambda_2^{m_2}}{m_2!}\mathrm{e}^{-\lambda_2}, \tag{2}$$

where $\lambda_i = k_b^{(i)}/k_d^{(i)}$ for $i \in \{1, 2\}$. In order to couple the genes together we allow the transcription rates $k_b^{(1)}$ and $k_b^{(2)}$ to depend upon the concentration of a shared upstream regulator, gene X. Let x denote the concentration of X and let $\rho(x)$ be the stationary probability density function for x. Taking birth rate as $k_b^{(i)}x$, the stationary joint PMF is then obtained from Bayes' theorem:

$$\begin{aligned} p(m_1, m_2) &= \int_0^\infty p(m_1, m_2 \,|\, x)\rho(x)\,dx, \\ &= \int_0^\infty \frac{(\lambda_1 x)^{m_1}}{m_1!}\mathrm{e}^{-\lambda_1 x} \cdot \frac{(\lambda_2 x)^{m_2}}{m_2!}\mathrm{e}^{-\lambda_2 x}\rho(x)\,dx. \end{aligned} \tag{3}$$

If $x \sim \mathrm{Gamma}(r, \theta)$ then this gives

$$\begin{aligned} p(m_1, m_2) &= \int_0^\infty \frac{(\lambda_1 x)^{m_1}}{m_1!}\mathrm{e}^{-\lambda_1 x} \cdot \frac{(\lambda_2 x)^{m_2}}{m_2!}\mathrm{e}^{-\lambda_2 x} \cdot \frac{x^{r-1}e^{-\frac{x}{\theta}}}{\Gamma(r)\theta^r}\,dx, \\ &= \frac{\Gamma(m_1 + m_2 + r)}{m_1!\,m_2!\,\Gamma(r)}(1 - p - q)^r p^{m_1} q^{m_2}. \end{aligned} \tag{4}$$

where $p = \lambda_1\theta/[1+\theta(\lambda_1+\lambda_2)]$ and $q = ap$ with $a = \lambda_2/\lambda_1$. Thus, the joint PMF is a bivariate negative binomial distribution. Note that the marginal distributions are negative binomial distributions, with probability $p' = \lambda_1\theta/(1 + \lambda_1\theta)$ for allele 1 and $p'' = \lambda_2\theta/(1 + \lambda_2\theta)$ for allele 2. For instance, for allele 1:

$$\begin{aligned} p(m_1) &= \int_0^\infty p(m_1|x)\rho(x)\,dx, \\ &= \int_0^\infty \frac{(\lambda_1 x)^{m_1}}{m_1!}\mathrm{e}^{-\lambda_1 x} \cdot \frac{x^{r-1}e^{-\frac{x}{\theta}}}{\Gamma(r)\theta^r}\,dx, \\ &= \frac{\Gamma(m_1 + r)}{m_1!\Gamma(r)}p'^{m_1}(1 - p')^r. \end{aligned} \tag{5}$$

The covariance between m_1 and m_2,

$$\mathrm{Cov}(m_1, m_2) = \mathbb{E}(m_1 m_2) - \mathbb{E}(m_1)\mathbb{E}(m_2), \tag{6}$$

may be obtained from the probability generating function for $p(m_1, m_2)$, which in this case is:

$$\phi(u, v) = \mathbb{E}[u^{m_1} v^{m_2}] = [1 + \lambda_1\theta(1 - u) + \lambda_2\theta(1 - v)]^{-r}. \tag{7}$$

In particular,

$$\mathbb{E}(m_1) = \left.\frac{\partial\phi}{\partial u}\right|_{u,v=1} = \frac{rp}{1-(p+q)}, \tag{8}$$

$$\mathbb{E}(m_2) = \left.\frac{\partial\phi}{\partial v}\right|_{u,v=1} = \frac{arp}{1-(p+q)}, \tag{9}$$

$$\mathbb{E}(m_1 m_2) = \left.\frac{\partial^2\phi}{\partial u\partial v}\right|_{u,v=1} = \frac{ap^2 r(r+1)}{(1-(p+q))^2}, \tag{10}$$

and therefore,

$$\text{Cov}(m_1, m_2) = \mathbb{E}(m_1 m_2) - \mathbb{E}(m_1)\mathbb{E}(m_2) = \frac{arp^2}{(1-(p+q))^2}. \tag{11}$$

This may be expressed in an alternative form as

$$\text{Cov}(m_1, m_2) = \lambda_1\lambda_2 r\theta^2 = \lambda_1\lambda_2\text{Var}(x). \tag{12}$$

Thus, the covariance of the target genes is proportional to both the variance of the upstream regulator and the sensitivities of the two targets to the upstream regulator. The correlation between m_1 and m_2 may also be similarly calculated. We obtain:

$$\text{Corr}(m_1, m_2) = \sqrt{\frac{\lambda_1\lambda_2 F^2(x)}{(1+\lambda_1 F(x))(1+\lambda_2 F(x))}}, \tag{13}$$

where $F(x) = \text{Var}(x)/\mathbb{E}(x)$ is the Fano factor (also known as the index of dispersion) of the upstream regulator x. Since,

$$\lim_{F(x)\to 0}\text{Corr}(m_1, m_2) = 0 \qquad \text{and} \qquad \lim_{F(x)\to\infty}\text{Corr}(m_1, m_2) = 1, \tag{14}$$

over-dispersion in the upstream regulator increases the correlation between downstream targets and under-dispersion reduces the correlation between targets. If the alleles are kinetically identical ($\lambda_1 = \lambda_2 = \lambda$) then

$$\text{Corr}(m_1, m_2) = \frac{\lambda F(x)}{1+\lambda F(x)}. \tag{15}$$

and the correlation between the alleles grows hyperbolically with the dispersion of the upstream regulator.

While the form of joint PMF given in Eq.(4) depends upon the upstream regulator being Gamma distributed, Eqs.(12)-(13) hold true for any upstream distribution $\rho(x)$ with nonnegative support. In general the probability generating function for the joint PMF $p(m_1, m_2)$ has the form:

$$\begin{aligned}
\phi(u,v) &= \sum_{m_1=0}^{\infty}\sum_{m_2=0}^{\infty} p(m_1,m_2)u^{m_1}v^{m_2}, \\
&= \sum_{m_1=0}^{\infty}\sum_{m_2=0}^{\infty}\left[\int_0^{\infty}\frac{(\lambda_1 x)^{m_1}}{m_1!}\mathrm{e}^{-\lambda_1 x}\cdot\frac{(\lambda_2 x)^{m_2}}{m_2!}\mathrm{e}^{-\lambda_2 x}\rho(x)\,dx\right]u^{m_1}v^{m_2}, \\
&= \int_0^{\infty}\mathrm{e}^{-x(\lambda_1+\lambda_2)}\rho(x)\left[\sum_{m_1=0}^{\infty}\sum_{m_2=0}^{\infty}\frac{(\lambda_1 ux)^{m_1}(\lambda_2 vx)^{m_2}}{m_1!m_2!}\right]dx, \\
&= \int_0^{\infty}\mathrm{e}^{-x(\lambda_1+\lambda_2)}\,\rho(x)\,\mathrm{e}^{x(\lambda_1 u+\lambda_2 v)}\,dx, \\
&= \int_0^{\infty}\rho(x)\,\mathrm{e}^{x(\lambda_1(u-1)+\lambda_2(v-1))}\,dx.
\end{aligned} \tag{16}$$

Thus,

$$\mathbb{E}(m_1) = \left.\frac{\partial \phi}{\partial u}\right|_{u,v=1} = \int_0^\infty \lambda_1\, x\, \rho(x)\, dx = \lambda_1 \mathbb{E}(x), \tag{17}$$

$$\mathbb{E}(m_2) = \left.\frac{\partial \phi}{\partial v}\right|_{u,v=1} = \int_0^\infty \lambda_2\, x\, \rho(x)\, dx = \lambda_2 \mathbb{E}(x), \tag{18}$$

$$\mathbb{E}(m_1 m_2) = \left.\frac{\partial^2 \phi}{\partial u \partial v}\right|_{u,v=1} = \int_0^\infty \lambda_1\lambda_2\, x^2\, \rho(x)\, dx = \lambda_1\lambda_2 \left(\text{Var}(x) + \mathbb{E}(x)^2\right). \tag{19}$$

Therefore,

$$\text{Cov}(m_1, m_2) = \mathbb{E}(m_1 m_2) - \mathbb{E}(m_1)\mathbb{E}(m_2) = \lambda_1\lambda_2\, \text{Var}(x) \tag{20}$$

as before. To find the correlation of downstream targets, we also need to find Var(m_1) and Var(m_2). We do so by using:

$$\mathbb{E}(m_1(m_1-1)) = \left.\frac{\partial^2 \phi}{\partial u^2}\right|_{u,v=1} = \int_0^\infty \lambda_1^2\, x^2\, \rho(x)\, dx = \lambda_1^2 \left(\text{Var}(x) + \mathbb{E}(x)^2\right), \tag{21}$$

$$\mathbb{E}(m_2(m_2-1)) = \left.\frac{\partial^2 \phi}{\partial v^2}\right|_{u,v=1} = \int_0^\infty \lambda_2^2\, x^2\, \rho(x)\, dx = \lambda_2^2 \left(\text{Var}(x) + \mathbb{E}(x)^2\right). \tag{22}$$

Hence, as $\mathbb{E}(z(z-1)) = \text{Var}(z) - \mathbb{E}(z) - \mathbb{E}(z)^2$ we obtain

$$\text{Var}(m_1) = \mathbb{E}(m_1(m_1-1)) + \mathbb{E}(m_1) + \mathbb{E}(m_1)^2 = \lambda_1^2\, \text{Var}(x) + \lambda_1\, \mathbb{E}(x), \tag{23}$$

$$\text{Var}(m_2) = \mathbb{E}(m_2(m_2-1)) + \mathbb{E}(m_2) + \mathbb{E}(m_2)^2 = \lambda_2^2\, \text{Var}(x) + \lambda_2\, \mathbb{E}(x), \tag{24}$$

thus giving

$$\begin{aligned}\text{Corr}(m_1, m_2) &= \frac{\lambda_1\lambda_2 \text{Var}(x)}{\sqrt{(\lambda_1^2\, \text{Var}(x) + \lambda_1\, \mathbb{E}(x))(\lambda_2^2\, \text{Var}(x) + \lambda_2\, \mathbb{E}(x))}}, \\ &= \sqrt{\frac{\lambda_1\lambda_2 F^2(x)}{(1+\lambda_1 F(x))(1+\lambda_2 F(x))}},\end{aligned} \tag{25}$$

as before.

If the mRNA birth process is not linearly dependent on x, but instead is determined by some arbitrary dependence $f(x)$, then the probability generating function for $p(m_1, m_2)$ is given by

$$\phi(u, v) = \int_0^\infty \rho(x)\, \mathrm{e}^{f(x)\, (\lambda_1(u-1)+\lambda_2(v-1))}\, dx. \tag{26}$$

In this case, $\mathbb{E}(m_1)$, $\mathbb{E}(m_2)$, Var(m_1), Var(m_2) and Cov(m_1, m_2) take a similar form as above, but with $\mathbb{E}(f(x))$ and Var$(f(x))$ replacing $\mathbb{E}(x)$ and Var(x) respectively. Thus,

$$\text{Cov}(m_1, m_2) = \lambda_1\lambda_2 \text{Var}(f(x)),$$

and

$$\text{Corr}(m_1, m_2) = \sqrt{\frac{\lambda_1\lambda_2 F^2(f(x))}{(1+\lambda_1 F(f(x)))(1+\lambda_2 F(f(x)))}}. \tag{27}$$

1.1.2 One two-state allele with upstream regulation

Consider the following dynamics in which gene G transitions stochastically between in 2 different states G_+ and G_- at constant rates ω_+ and ω_-, with the rate of transcription of M depending upon the state of the gene:

$$G_- \underset{\omega_-}{\overset{\omega_+}{\rightleftarrows}} G_+, \qquad G_- \xrightarrow{k_b^-} G_- + M, \qquad G_+ \xrightarrow{k_b^+} G_+ + M, \qquad M \xrightarrow{k_d} \emptyset. \tag{28}$$

Without loss of generality we take $k_b^+ > k_b^-$. Let p_m^z denote the conditional probability $p(M = m|G = G_z)$ for $z \in \{+, -\}$. The dynamics are described by the master equation:

$$\frac{\partial p_m^z}{\partial t} = -k_b^z p_m^z - mk_d p_m^z + k_b^z p_{m-1}^z + k_d(m+1)p_{m+1}^z + \sum_{z' \neq z} \Omega_{zz'} p_m^{z'}, \tag{29}$$

where the matrix

$$\Omega_{zz'} = \begin{pmatrix} -\omega_+ & \omega_- \\ \omega_+ & -\omega_- \end{pmatrix} \tag{30}$$

is given in terms of the transition rates ω_+ and ω_- into the active and inactive states respectively. To solve Eq. (29) it is convenient to reformulate in terms of the probability generating functions $\phi_z(x) = \sum_n p_m^z x^n$, whence we obtain a pair of coupled partial differential equations for ϕ_+ and ϕ_-:

$$\frac{\partial \phi_\pm}{\partial t} = -y \left(\frac{\partial}{\partial y} - \lambda_\pm \right) \phi_\pm \pm \varepsilon_+ \phi_- \mp \varepsilon_- \phi_+, \tag{31}$$

where $y = x - 1$, $\lambda_\pm = k_b^\pm / k_d$, $\varepsilon_\pm = \omega_\pm / k_d$, and we have rescaled time with the degradation rate k_d. In the limit $\varepsilon_\pm \to 0$ (i.e. transition rates between gene states are small with respect to the mRNA degradation rate) we may obtain an approximation to the stationary solution to Eq. (31) by considering an asymptotic expansion of the form $\phi_\pm = \phi_\pm^0 + \varepsilon_\pm \phi_\pm^1 + \ldots$. Substituting this anzatz into Eq. (31) we obtain $\phi_\pm^0 = \exp(\lambda_\pm(x-1))$, which is the probability generating function for the Poisson distribution. Thus,

$$p_m^\pm = \frac{\lambda_\pm^m}{m!} \mathrm{e}^{-\lambda_\pm} + \mathcal{O}(\varepsilon_\pm). \tag{32}$$

The leading order stationary distribution $p(m)$ may then be obtained from Bayes' theorem:

$$\begin{aligned} p(m) &\sim \sum_z p(z) p(m|z), \\ &\sim \sum_z p(z) p_m^z, \\ &\sim w p_m^+ + (1-w) p_m^-, \end{aligned} \tag{33}$$

where w is the probability of the gene being in the positive state (and therefore $1 - w$ is the probability that the gene is in the negative state). By conservation of probability

$$\sum_{z'} \Omega_{zz'} \pi_{z'} = 0, \tag{34}$$

which gives $w = \omega_+ / (\omega_- + \omega_+)$. Thus, in the limit $\varepsilon_\pm \to 0$ the stationary pmf for y is approximated by a Poisson mixture:

$$p(m) = w \frac{\lambda_+^m}{m!} \mathrm{e}^{-\lambda_+} + (1-w) \frac{\lambda_-^m}{m!} \mathrm{e}^{-\lambda_-} + \mathcal{O}(\varepsilon^*), \tag{35}$$

where $\varepsilon^* = \max \varepsilon_\pm$. If we now allow the transcription rates of m from each state to be proportional to the Gamma distributed concentration of the upstream regulator X as before then via Bayes theorem we obtain:

$$p(m) \approx \int_0^\infty \left[w \frac{(\lambda_+ x)^m}{m!} \mathrm{e}^{-\lambda_+ x} + (1-w) \frac{(\lambda_- x)^m}{m!} \mathrm{e}^{-\lambda_- x} \right] \times \left[\frac{x^{r-1}}{\theta^k \Gamma(r)} \mathrm{e}^{-x/\theta} \right] dx. \tag{36}$$

Integrating gives,

$$p(m) \sim w \frac{\Gamma(m+r)}{\Gamma(r) m!} p_1^m (1-p_1)^r + (1-w) \frac{\Gamma(m+r)}{\Gamma(r) m!} p_2^m (1-p_2)^r, \tag{37}$$

where $p_1 = \theta\lambda_+ / (1 + \theta\lambda_+)$ and $p_2 = \theta\lambda_- / (1 + \theta\lambda_-)$. Thus, m follows a two-component negative binomial mixture, characterised by 4 parameters (w, r, p_1, p_2). This argument may be extended to a gene with n states, each with different sensitivities to the upstream regulator. In this case the target follows an n-component negative binomial mixture, i.e. $m \sim \sum_{i=1}^n w_i \mathrm{NB}(r, p_i)$, with $p_i = \theta\lambda_i / (1 + \theta\lambda_i)$ and $w_i = \omega_i / \sum_i \omega_i$.

1.1.3 Two two-state alleles with upstream regulation

Now consider the following dynamics in which there are 2 genes, $G^{(i)}$ ($i = 1, 2$), which both transition stochastically between in 2 different states, $G_+^{(i)}$ and $G_-^{(i)}$, at constant rates $\omega_+^{(i)}$ and $\omega_-^{(i)}$. Let m_1 denote the number of M_1 mRNA transcripts associated with gene $G^{(1)}$; and let m_2 denote the number of M_2 mRNA transcripts associated with gene $G^{(2)}$. Both genes respond to an upstream regulator X with concentration x which is Gamma distributed. The dynamics are as follows:

$$G_-^{(i)} \underset{\omega_+^{(i)}}{\overset{\omega_-^{(i)}}{\rightleftarrows}} G_+^{(i)}, \qquad G_- \xrightarrow{k_{b-}^{(i)}x} G_- + M_i, \qquad G_+^{(i)} \xrightarrow{k_{b+}^{(i)}x} G_+^{(i)} + M_i, \qquad M_i \xrightarrow{k_d^{(i)}} \emptyset, \tag{38}$$

for $i = 1, 2$. Assuming that $\omega_+^{(i)}$, $\omega_-^{(i)} \ll k_d^{(i)}$ the stationary marginal distributions are both approximated by two component negative binomial mixtures, characterised by the parameters $w^{(i)}, \lambda_+^{(i)}, \lambda_-^{(i)}, r, p_1^{(i)}, p_2^{(i)}$ (exactly as before, see Eq. (37)). Since the expression of M_1 and M_2 are independent conditioned on the concentration of the upstream regulator x [i.e. $p(m_1, m_2|x) = p(m_1|x)p(m_2|x)$] the leading order stationary joint distribution is given by:

$$\begin{aligned} p(m_1, m_2) = \int_0^\infty & \left[w^{(1)} \frac{(\lambda_+^{(1)}x)^{m_1} e^{-\lambda_+^{(1)}x}}{m_1!} + (1 - w^{(1)}) \frac{(\lambda_-^{(1)}x)^{m_1} e^{-\lambda_-^{(1)}x}}{m_1!} \right] \\ & \times \left[w^{(2)} \frac{(\lambda_+^{(2)}x)^{m_2} e^{-\lambda_+^{(2)}x}}{m_2!} + (1 - w^{(2)}) \frac{(\lambda_-^{(2)}x)^{m_2} e^{-\lambda_-^{(2)}x}}{m_2!} \right] \rho(x)dx. \end{aligned} \tag{39}$$

Assuming that $x \sim \text{Gamma}(r, \theta)$ we obtain:

$$\begin{aligned} p(m_1, m_2) \sim\ & w^{(1)}w^{(2)}\text{BNB}(r, p_a, q_a) \\ & + w^{(1)}(1 - w^{(2)})\text{BNB}(r, p_b, q_b) \\ & + w^{(2)}(1 - w^{(1)})\text{BNB}(r, p_c, q_c) \\ & + (1 - w^{(1)})(1 - w^{(2)})\text{BNB}(r, p_d, q_d), \end{aligned} \tag{40}$$

where

$$\begin{aligned} p_a &= \tfrac{\lambda_+^{(1)}\theta}{1+\theta(\lambda_+^{(1)}+\lambda_+^{(2)})}, & q_a &= \alpha_a p_a, & \alpha_a &= \tfrac{\lambda_+^{(2)}}{\lambda_+^{(1)}}, \\ p_b &= \tfrac{\lambda_+^{(1)}\theta}{1+\theta(\lambda_+^{(1)}+\lambda_-^{(2)})}, & q_b &= \alpha_b p_b, & \alpha_b &= \tfrac{\lambda_-^{(2)}}{\lambda_+^{(1)}}, \\ p_c &= \tfrac{\lambda_-^{(1)}\theta}{1+\theta(\lambda_-^{(1)}+\lambda_+^{(2)})}, & q_c &= \alpha_c p_c, & \alpha_c &= \tfrac{\lambda_-^{(2)}}{\lambda_-^{(1)}}, \\ p_d &= \tfrac{\lambda_-^{(1)}\theta}{1+\theta(\lambda_-^{(1)}+\lambda_-^{(2)})}, & q_d &= \alpha_d p_d, & \alpha_d &= \tfrac{\lambda_-^{(2)}}{\lambda_-^{(1)}} \end{aligned} \tag{41}$$

and $\text{BNB}(r, p, q)$ denotes the bivariate negative binomial distribution with PMF

$$p(m_1, m_2) = \frac{\Gamma(m_1 + m_2 + r)}{m_1!\, m_2!\, \Gamma(r)} (1 - p - q)^r p^{m_1} q^{m_2}. \tag{42}$$

Following a similar process as in section 1.1.2, it is possible to generalise this result for any upstream regulator distribution. Eq. (39) can be rewritten as

$$p(m_1, m_2) = \int_0^\infty (AC + BC + AD + BD)\rho(x)dx, \tag{43}$$

where

$$\begin{aligned} A &= w^{(1)} \tfrac{(\lambda_+^{(1)}x)^{m_1} e^{-\lambda_+^{(1)}x}}{m_1!}, & B &= (1 - w^{(1)}) \tfrac{(\lambda_-^{(1)}x)^{m_1} e^{-\lambda_-^{(1)}x}}{m_1!}, \\ C &= w^{(2)} \tfrac{(\lambda_+^{(2)}x)^{m_2} e^{-\lambda_+^{(2)}x}}{m_2!}, & D &= (1 - w^{(2)}) \tfrac{(\lambda_-^{(2)}x)^{m_2} e^{-\lambda_-^{(2)}x}}{m_2!}. \end{aligned} \tag{44}$$

The probability generating function can therefore be written as:

$$\begin{aligned}
\phi(u,v) &= \sum_{m_1=0}^{\infty}\sum_{m_2=0}^{\infty} p(m_1,m_2)u^{m_1}v^{m_2}, \\
&= \sum_{m_1=0}^{\infty}\sum_{m_2=0}^{\infty}\left[\int_0^{\infty}(AC+BC+AD+BD)\rho(x)\,dx\right]u^{m_1}v^{m_2}, \\
&= \int_0^{\infty}\rho(x)\left[\sum_{m_1=0}^{\infty}\sum_{m_2=0}^{\infty} ACu^{m_1}v^{m_2}\right] + \int_0^{\infty}\rho(x)\left[\sum_{m_1=0}^{\infty}\sum_{m_2=0}^{\infty} BCu^{m_1}v^{m_2}\right] \\
&+ \int_0^{\infty}\rho(x)\left[\sum_{m_1=0}^{\infty}\sum_{m_2=0}^{\infty} ADu^{m_1}v^{m_2}\right] + \int_0^{\infty}\rho(x)\left[\sum_{m_1=0}^{\infty}\sum_{m_2=0}^{\infty} BDu^{m_1}v^{m_2}\right], \\
&= X+Y+Z+T
\end{aligned} \tag{45}$$

Where

$$\begin{aligned}
X &= \int_0^{\infty}\rho(x)\left[\sum_{m_1=0}^{\infty}\sum_{m_2=0}^{\infty} ACu^{m_1}v^{m_2}\right], \\
&= \int_0^{\infty} w^{(1)}w^{(2)}\rho(x)e^{-x(\lambda_+^{(1)}+\lambda_+^{(2)})}\sum_{m_1=0}^{\infty}\sum_{m_2=0}^{\infty}\left[\frac{(\lambda_+^{(1)}ux)^{m_1}(\lambda_+^{(2)}vx)^{m_2}}{m_1!m_2!}\right]dx, \\
&= \int_0^{\infty} w^{(1)}w^{(2)}\rho(x)e^{-x(\lambda_+^{(1)}+\lambda_+^{(2)})}e^{x(\lambda_+^{(1)}u+\lambda_+^{(2)}v)}dx, \\
&= \int_0^{\infty} w^{(1)}w^{(2)}\rho(x)e^{x\lambda_+^{(1)}(u-1)}e^{x\lambda_+^{(2)}(v-1)}dx.
\end{aligned} \tag{46}$$

Similarly,

$$Y = \int_0^{\infty}(1-w^{(1)})w^{(2)}\rho(x)e^{x\lambda_-^{(1)}(u-1)}e^{x\lambda_+^{(2)}(v-1)}dx, \tag{47}$$

$$Z = \int_0^{\infty} w^{(1)}(1-w^{(2)})\rho(x)e^{x\lambda_+^{(1)}(u-1)}e^{x\lambda_-^{(2)}(v-1)}dx, \tag{48}$$

$$T = \int_0^{\infty}(1-w^{(1)})(1-w^{(2)})\rho(x)e^{x\lambda_-^{(1)}(u-1)}e^{x\lambda_-^{(2)}(v-1)}dx. \tag{49}$$

Expected values are found from the probability generating function:

$$\mathbb{E}(m_1) = \left.\frac{\partial\phi}{\partial u}\right|_{u,v=1} = \left.\frac{\partial X}{\partial u}\right|_{u,v=1} + \left.\frac{\partial Y}{\partial u}\right|_{u,v=1} + \left.\frac{\partial Z}{\partial u}\right|_{u,v=1} + \left.\frac{\partial T}{\partial u}\right|_{u,v=1}. \tag{50}$$

As

$$\begin{aligned}
\left.\frac{\partial X}{\partial u}\right|_{u,v=1} &= w^{(1)}w^{(2)}\int_0^{\infty}\rho(x)x\lambda_+^{(1)}dx = w^{(1)}w^{(2)}\lambda_+^{(1)}\mathbb{E}(x), \\
\left.\frac{\partial Y}{\partial u}\right|_{u,v=1} &= (1-w^{(1)})w^{(2)}\int_0^{\infty}\rho(x)x\lambda_-^{(1)}dx = (1-w^{(1)})w^{(2)}\lambda_-^{(1)}\mathbb{E}(x), \\
\left.\frac{\partial Z}{\partial u}\right|_{u,v=1} &= w^{(1)}(1-w^{(2)})\int_0^{\infty}\rho(x)x\lambda_+^{(1)}dx = w^{(1)}(1-w^{(2)})\lambda_+^{(1)}\mathbb{E}(x), \\
\left.\frac{\partial T}{\partial u}\right|_{u,v=1} &= (1-w^{(1)})(1-w^{(2)})\int_0^{\infty}\rho(x)x\lambda_-^{(1)}dx = (1-w^{(1)})(1-w^{(2)})\lambda_-^{(1)}\mathbb{E}(x).
\end{aligned} \tag{51}$$

This results in:

$$\mathbb{E}(m_1) = (w^{(1)}\lambda_+^{(1)} + (1 - w^{(1)})\lambda_-^{(1)})\mathbb{E}(x). \tag{52}$$

Similarly,

$$\mathbb{E}(m_2) = (w^{(2)}\lambda_+^{(2)} + (1 - w^{(2)})\lambda_-^{(2)})\mathbb{E}(x). \tag{53}$$

In order to find the covariance of the joint distribution we also need to calculate $\mathbb{E}(m_1, m_2)$:

$$\mathbb{E}(m_1, m_2) = \left.\frac{\partial \phi}{\partial u, \partial v}\right|_{u,v=1} = \left.\frac{\partial X}{\partial u, \partial v}\right|_{u,v=1} + \left.\frac{\partial Y}{\partial u, \partial v}\right|_{u,v=1} + \left.\frac{\partial Z}{\partial u, \partial v}\right|_{u,v=1} + \left.\frac{\partial T}{\partial u, \partial v}\right|_{u,v=1}. \tag{54}$$

Using,

$$\begin{aligned}
\left.\frac{\partial X}{\partial u, \partial v}\right|_{u,v=1} &= w^{(1)}w^{(2)}\lambda_+^{(1)}\lambda_+^{(2)} \int_0^\infty x^2\rho(x)dx = w^{(1)}w^{(2)}\lambda_+^{(1)}\lambda_+^{(2)}(\text{Var(x)} + \mathbb{E}(x)^2),\\
\left.\frac{\partial Y}{\partial u, \partial v}\right|_{u,v=1} &= (1 - w^{(1)})w^{(2)}\lambda_-^{(1)}\lambda_+^{(2)} \int_0^\infty x^2\rho(x)dx = (1 - w^{(1)})w^{(2)}\lambda_-^{(1)}\lambda_+^{(2)}(\text{Var(x)} + \mathbb{E}(x)^2),\\
\left.\frac{\partial Z}{\partial u, \partial v}\right|_{u,v=1} &= w^{(1)}(1 - w^{(2)})\lambda_+^{(1)}\lambda_-^{(2)} \int_0^\infty x^2\rho(x)dx = w^{(1)}(1 - w^{(2)})\lambda_+^{(1)}\lambda_-^{(2)}(\text{Var(x)} + \mathbb{E}(x)^2),\\
\left.\frac{\partial T}{\partial u, \partial v}\right|_{u,v=1} &= (1 - w^{(1)})(1 - w^{(2)})\lambda_-^{(1)}\lambda_-^{(2)} \int_0^\infty x^2\rho(x)dx = (1 - w^{(1)})(1 - w^{(2)})\lambda_-^{(1)}\lambda_-^{(2)}(\text{Var(x)} + \mathbb{E}(x)^2),
\end{aligned} \tag{55}$$

it can be seen that,

$$\mathbb{E}(m_1, m_2) = (w^{(1)}\lambda_+^{(1)} + (1 - w^{(1)})(w^{(2)}\lambda_+^{(2)} + (1 - w^{(2)})\lambda_-^{(2)}))(\text{Var}(x) + \mathbb{E}(x)^2). \tag{56}$$

In general, the covariance between the output mRNA of two bursting genes is given by:

$$\begin{aligned}
\text{Cov}(m_1, m_2) &= \mathbb{E}(m_1, m_2) - \mathbb{E}(m_1)\mathbb{E}(m_2),\\
&= (w^{(1)}\lambda_+^{(1)} + (1 - w^{(1)})(w^{(2)}\lambda_+^{(2)} + (1 - w^{(2)})\lambda_-^{(2)}))\,\text{Var}(x).
\end{aligned} \tag{57}$$

For two alleles of the same gene, we assume that the switching rates between gene states and the transcription rates from each state are the same; $\lambda_+^{(1)} = \lambda_+^{(2)} = \lambda_+$, $\lambda_-^{(1)} = \lambda_-^{(2)} = \lambda_-$ and $w^{(1)} = w^{(2)} = w$. Hence,

$$\text{Cov}(m_1, m_2) = (w\lambda_+ + (1 - w)\lambda_-)^2\,\text{Var}(x). \tag{58}$$

As for one-state genes, the covariance between allelic mRNA outputs of two-state genes is dependent on the variance of a common upstream regulator. Fig. S1 demonstrates how increasing variance of an upstream regulator (shown for Gamma-distributed regulator concentration, x), affects the joint distribution of downstream alleles for a two-state gene and increases covariance.

1.2 Bifurcation curves for Nanog dynamics

The dynamics for all the reporter strategies that we consider can be described by the following dimensionless ordinary differential equation (ODE) for total Nanog concentration $\bar{n}$ (see main text and below),

$$\frac{d\bar{n}}{d\tau} = \alpha + \frac{\bar{n}^H}{\gamma^H + \bar{n}^H} - \bar{n}. \tag{59}$$

Fixed points solutions, in which $d\bar{n}/d\tau = 0$, satisfy the polynomial

$$0 = \gamma^H(\alpha - \bar{n}) + (\alpha + 1)\bar{n}^H - \bar{n}^{H+1}. \tag{60}$$

This polynomial has either 1 or three real solutions, depending on the values of α and γ and the Hill coefficient H. When there is only one real solution the system has one stable fixed point and the resulting Nanog is unimodal; when there are three real

solutions, two of them are stable and the Nanog distribution is bimodal. The threshold between these regimes occurs when there is a repeated solution, which occurs when the discriminant Δ of Eq. (60) is zero. In the case $H = 2$

$$\Delta = \gamma^2 + \left(2\alpha^2 - 5\alpha - \frac{1}{4}\right)\gamma + \alpha\left(1 + \alpha^3\right). \tag{61}$$

Thus $\Delta = 0$ is a quadratic for γ which has roots

$$\gamma_{\pm}(\alpha) = -\left(\alpha^2 - \frac{5}{2}\alpha - \frac{1}{8}\right) \pm \left(\frac{1}{4} - 2\alpha\right)^{\frac{3}{2}}, \tag{62}$$

which are the bifurcation curves given in the main text. If the model parameters fall *inside* the region enclosed by these curves then Nanog expression is bimodal, corresponding to the coexistence of a Nanog high and Nanog low expressing sub-populations of cells; if the model parameters fall *outside* this region then Nanog expression is unimodal, corresponding to a homogeneous population of Nanog high or Nanog low expressing cells. A similar calculation may be performed for arbitrary $H \in \mathbb{Z}^+$.

1.3 Reporter perturbations

To understand how reporters may affect endogeneous Nanog dynamics we compared the dynamics of Nanog in various different reporter lines with those in wild-type ES cells. To recap from the main text, Nanog expression in wild-type cells is described by the following ODEs:

$$\frac{dn_1}{dt} = c_b + \frac{c_f n^H}{K^H + n^H} - c_d n_1, \tag{63}$$

$$\frac{dn_2}{dt} = c_b + \frac{c_f n^H}{K^H + n^H} - c_d n_2, \tag{64}$$

where n_i denotes the concentration of the Nanog protein output of allele $i \in \{1, 2\}$, $n = (n_1 + n_2)$ is total Nanog concentration. Combining these equations we obtain an ODE for the total Nanog protein concentration:

$$\frac{dn}{dt} = 2c_b + 2\frac{c_f n^H}{K^H + n^H} - c_d n \tag{65}$$

Nondimensionalizing using the scalings $n = 2c_f c_d^{-1}\bar{n}$, and $t = c_d^{-1}\tau$ we obtain:

$$\frac{d\bar{n}}{d\tau} = \alpha + \frac{\bar{n}^H}{\gamma_{\text{wt}}^H + \bar{n}^H} - \bar{n} \tag{66}$$

where $\bar{n}$ is the dimensionless total Nanog concentration and τ is dimensionless time. The dimensionless constants $\alpha = c_b/c_f$ and $\gamma = \gamma_{\text{wt}} = c_d K/2c_f$ measure the strength of the baseline production rate and the strength of the Nanog autoregulatory feedback loop respectively. We now consider how Nanog dynamics given by Eq.(66) are perturbed by a variety of different kinds of reporters. In all cases, for clarity of exposition, the reporter proteins are assumed to decay with the same kinetics as Nanog. This assumption may be weakened without affecting our conclusions. The results of this section are also summarised in Supporting Tables 1 & 2 which also give the relationships between reporter and Nanog concentrations at equilibrium as a measure of the quantitative accuracy of each reporter.

1.4 Single allele reporter strategies

1.4.1 Knock-in reporters

Knock-in reporters reporters remove the Nanog protein coding region from one allele and replace it with a reporter gene under the same promoter control. In this case, the kinetics described by Eqs. (65) are modified to

$$\frac{dn}{dt} = c_b + \frac{c_f n^H}{K^H + n^H} - c_d n, \tag{67}$$

$$\frac{dr}{dt} = c_b + \frac{c_f n^H}{K^H + n^H} - c_d r, \tag{68}$$

where n denotes Nanog concentration and r denotes reporter concentration. Using the scalings $n = c_f c_d^{-1}\bar{n}$ and $t = c_d^{-1}\tau$ the dimensionless equation for total Nanog concentration in the knock-in reporter line is:

$$\frac{d\bar{n}}{d\tau} = \alpha + \frac{\bar{n}^H}{\gamma_{\text{ki}}^H + \bar{n}^H} - \bar{n}, \tag{69}$$

where α is as before, but $\gamma_{\text{ki}} = 2\gamma_{\text{wt}}$. In this case, the loss of Nanog production from one allele has the effect of diminishing the functional Nanog production rate by a factor of two, which effectively weakens the endogenous feedback mechanisms and thereby doubles γ. Since the magnitude of γ determines if Nanog is homogeneously or heterogeneously expressed in the population, this change can induce a heterogeneous Nanog expression pattern in a reporter cell line that is not characteristic of the wild-type (or vice versa). From Eqs. (67)-(68) at equilibrium $r = n$ so it is expected that the knock-in reporter signal will faithfully represent Nanog expression in the engineered line.

1.4.2 Pre/post (PP) reporters

Single allele pre/post reporters insert the reporter gene either directly before or after the Nanog protein coding region on one Nanog allele. We assume that this insertion alters the Nanog production rate from the reporter allele by a factor $0 \leq \epsilon$. The following ODEs describe the dynamics in this case:

$$\frac{dn_1}{dt} = c_b + \frac{c_f n^H}{K^H + n^H} - c_d n_1, \tag{70}$$

$$\frac{dn_2}{dt} = \epsilon c_b + \epsilon \frac{c_f n^H}{K^H + n^H}, -c_d n_2 \tag{71}$$

$$\frac{dr}{dt} = \epsilon c_b + \epsilon \frac{c_f n^H}{K^H + n^H}, -c_d r \tag{72}$$

where n_i denotes the concentration of the Nanog protein output of allele $i \in \{1, 2\}$ and r denotes the reporter concentration (assumed without loss of generality to be produced from allele 2). Combining these equations for total Nanog $n = n_1 + n_2$ and using the scalings $n = c_f(1+\epsilon)c_d^{-1}\bar{n}$ and $t = c_d^{-1}\tau$, the dimensionless equation for total Nanog is:

$$\frac{d\bar{n}}{d\tau} = \alpha + \frac{\bar{n}^H}{\gamma_{\text{pp}}^H + \bar{n}^H} - \bar{n}, \tag{73}$$

where $\gamma_{\text{pp}} = 2\gamma_{\text{wt}}/(1+\epsilon)$. Thus, if the addition of the reporter gene completely halts transcription from allele 2 then $\epsilon = 0$ and $\gamma_{\text{pp}} = \gamma_{\text{ki}} = 2\gamma_{\text{wt}}$; if the reporter halves the rate of transcription from allele 2, as in Fig. 2C, then $\epsilon = 1/2$ and $\gamma_{\text{pp}} = 4\gamma_{\text{wt}}/3$; if the reporter does not affect the rate of transcription from allele 1, then $\epsilon = 1$ and $\gamma_{\text{pp}} = \gamma_{\text{wt}}$. For $0 < \epsilon < 1$ pre/post reporters are less likely than knock-in reporters to induce qualitative changes in Nanog expression dynamics, yet are still subject to similar systemic risk. Similar results are obtained for $1 < \epsilon$, see Fig. S2. From Eqs. (70)-(72) at equilibrium $r = \epsilon n/(1+\epsilon)$ so it is expected that, in addition to any qualitative perturbations, the PP reporter signal will quantitatively misrepresent Nanog expression by a factor $\epsilon/(1+\epsilon)$.

1.4.3 Multiple pre/post (MPP) reporters

If multiple (m) repeats of the reporter gene are inserted on the reporter allele then we assume that any production rates changes due to the reporter construct are compounded and transcription rate is altered by a factor ϵ_m where $0 \leq \epsilon_m \leq \epsilon < 1$ for a rate decrease and $1 < \epsilon \leq \epsilon_m$ for a rate increase. If m copies of the reporter transcript are produced (for non-tandem repeats) then the dynamics become

$$\frac{dn_1}{dt} = c_b + \frac{c_f n^H}{K^H + n^H} - c_d n_1, \tag{74}$$

$$\frac{dn_2}{dt} = \epsilon_m c_b + \epsilon_m \frac{c_f n^H}{K^H + n^H} - c_d n_2, \tag{75}$$

$$\frac{dr}{dt} = m\epsilon_m c_b + m\epsilon_m \frac{c_f n^H}{K^H + n^H} - c_d r. \tag{76}$$

Combining these equations and using the scalings $n = c_f(1+\epsilon_m)c_d^{-1}\bar{n}$ and $t = c_d^{-1}\tau$, the dimensionless equation for total Nanog is:

$$\frac{d\bar{n}}{d\tau} = \alpha + \frac{\bar{n}^H}{\gamma_{\text{mpp}}^H + \bar{n}^H} - \bar{n}, \tag{77}$$

where $\gamma_{\text{mpp}} = 2\gamma_{\text{wt}}/(1+\epsilon_m)$. If a single insert slows transcription by a factor $0 \leq \epsilon \leq 1$ and each of the m inserts is identical, then $\epsilon_m = \epsilon/(m(1-\epsilon)+\epsilon) \leq \epsilon$ (with equality if and only if $m = 1$). Thus, although multiple reporter additions improve fluorescent signal, the systemic risk is increased with each additional reporter insert. As m becomes large $\epsilon_m \to 0$ and this risk approaches that of the knock-in reporters. From Eqs. (74)-(76) at equilibrium $r = m\epsilon_m n/(1+\epsilon_m)$ so it is expected that, in addition to any qualitative perturbations, the MPP reporter signal will quantitatively misrepresent Nanog expression by a factor $m\epsilon_m/(1+\epsilon_m)$.

1.4.4 Fusion reporters

Fusion reporters produce a modified version of Nanog, which includes a fluorescence structure as part of the Nanog protein. For single allele fusion reporters, the fusion protein (concentration n_2) is produced from one allele, and the wild-type protein (concentration n_1) is produced from the other. This has two effects on the dynamics: (1) the rate of transcription from the reporter allele is reduced by a factor $0 \leq \epsilon \leq 1$ due to the additional DNA that must be transcribed, as for a PP reporter, and (2) the function of the Nanog from the reporter allele is compromised by a factor $0 \leq \delta \leq 1$ due to the addition of a cumbersome fluorescent protein to the native Nanog. The dynamics in this case are:

$$\frac{dn_1}{dt} = c_b + \frac{c_f n_{\text{eff}}^H}{K^H + n_{\text{eff}}^H} - c_d n_1, \tag{78}$$

$$\frac{dn_2}{dt} = \epsilon c_b + \epsilon\frac{c_f n_{\text{eff}}^H}{K^H + n_{\text{eff}}^H} - c_d n_2, \tag{79}$$

where $n_{\text{eff}} = n_1 + \delta n_2$. Combining these equations and nondimensionalising using the scalings $n_{\text{eff}} = c_f(1+\epsilon\delta)c_d^{-1}\bar{n}_{\text{eff}}$ and $t = c_d^{-1}\tau$ we obtain the following equation for the effective Nanog concentration:

$$\frac{d\bar{n}_{\text{eff}}}{d\tau} = \alpha + \frac{\bar{n}_{\text{eff}}^H}{\gamma_{\text{fus}}^H + \bar{n}_{\text{eff}}^H} - \bar{n}_{\text{eff}}, \tag{80}$$

where $\gamma_{\text{fus}} = 2\gamma_{\text{wt}}/(1+\epsilon\delta)$. If $\delta = 0$ then the Nanog-reporter fusion is not functional, while for $\delta = 1$ the Nanog-reporter fusion functions as the native Nanog protein. For $0 < \delta < 1$, $\gamma_{\text{fus}} > \gamma_{\text{pp}}$, therefore fusion reporters are more likely than pre/post reporters to induce qualitative changes in expression dynamics. From Eqs. (78)-(79) at equilibrium $r = \epsilon n/(1+\epsilon)$ so it is expected that, in addition to any qualitative perturbations, the fusion reporter signal will quantitatively misrepresent Nanog expression by a factor $r = \epsilon/(1+\epsilon)$.

1.4.5 BAC reporters

Bacterial artificial chromosome (BAC) reporters introduce a piece of extra-genomic DNA into the cell that encodes the Nanog gene under the control of the endogenous Nanog promoter and regulatory regions. Because this construct does not disturb the kinetics of either of the wild-type alleles, it (uniquely amongst the reporters we consider) does not directly affect the endogenous feedback mechanisms and is therefore the least likely reporter strategy to induce qualitative changes in Nanog dynamics. However, because the reporter construct is physically separated from the Nanog alleles, it is expected that the reporter protein expression is subject to extrinsic stochastic fluctuations which are independent to those of endogenous Nanog expression. For this reason we expect that BAC reporters are more susceptible to *technical* errors that the other constructs we consider.

1.5 Dual allele reporter strategies

Dual allele reporters can either express the same reporter molecule from both allele (e.g. both drive transcription of GFP) or may express different reporter molecules from different alleles (e.g. GFP from one allele, and a red fluorescent protein from the other). The analysis of the single allele reporters above may be easily modified to account for dual reporter strategies. The dynamics for total Nanog in dual reporter systems are given by:

$$\frac{d\bar{n}}{d\tau} = \alpha + \frac{\bar{n}^H}{\gamma_{\text{dual}}^H + \bar{n}^H} - \bar{n}, \tag{81}$$

where: (1) $\gamma_{\text{dual}} = \gamma_{\text{wt}}/\epsilon_m$ in the case of dual multiple pre/post reporters that produce m copies of the same fluorescent signal from both alleles (which reduces the rate of transcription from both alleles by a factor $0 \leq \epsilon_m \leq 1$ from both alleles); (2) $\gamma_{\text{dual}} = 2\gamma_{\text{wt}}/(\epsilon_{m1} + \epsilon_{m2})$ in the case of dual pre/post reporters that produce different reporters from the two alleles

(which reduces the rate of transcription from alleles 1 and 2 by factors $0 \leq \epsilon_{m1} \leq 1$ and $0 \leq \epsilon_{m2} \leq 1$ respectively); (3) $\gamma_{\text{dual}} = \gamma_{\text{wt}}/\epsilon\delta$ for dual fusion reporters that produce the same fusion protein from each allele (which reduces the rate of transcription from both alleles by a factor $0 \leq \epsilon \leq 1$ from both alleles, and compromises Nanog function by a factor δ); (4) $\gamma_{\text{dual}} = 2\gamma_{\text{wt}}/(\epsilon_1\delta_1 + \epsilon_2\delta_2)$ for dual fusion reporters that produce different fusion proteins from each allele (which reduce the rates of transcription by factors $0 \leq \epsilon_1 \leq 1$ and $0 \leq \epsilon_2 \leq 1$ from alleles 1 and 2 respectively, and compromise Nanog function by factors δ_1 and δ_2 respectively). It should be noted for dual allele fusion reporters there is no wild-type protein in the system at all which could have further unintended consequences, including off target effects. In all cases γ_{dual} is larger than the corresponding value of γ for the single allele reporters. Thus, while more technically accurate, dual allele reporters carry raised risk of systemic perturbations to the endogenous kinetics. In addition to any qualitative perturbations, dual reporter systems may also quantitatively misrepresent Nanog expression in similar ways to the corresponding single allele constructs. These perturbations are detailed in Supporting Table 2.

1.6 Decay constant mismatch

All the simplified reporters described above have identical decay rate constants for the reporter protein and Nanog. The effect of mismatched decay rates can be seen by examining the BAC reporter, which does not perturb α or γ. Governing ODEs are given below when Nanog and reporter molecules have decay rate constants c_{dn} and c_{dr} respectively:

$$\frac{dn}{dt} = 2c_b + 2\frac{c_f n^H}{K^H + n^H} - c_{dn}n, \tag{82}$$

$$\frac{dr}{dt} = c_b + \frac{c_f n^H}{K^H + n^H} - c_{dr}r. \tag{83}$$

Non-dimensionalisation using the scalings $t = c_{dn}^{-1}\tau$, $n = 2c_f c_{dn}^{-1}\bar{n}$ and $r = c_f c_{dr}^{-1}\bar{r}$, leads to the following dimensionless ODEs:

$$\begin{aligned} \frac{d\bar{n}}{d\tau} &= \alpha + \frac{\bar{n}^H}{\gamma_d^H + \bar{n}^H} - \bar{n}, \\ \frac{c_{dn}}{c_{dr}}\frac{d\bar{r}}{d\tau} &= \alpha + \frac{\bar{n}^H}{\gamma_d^H + \bar{n}^H} - \bar{r}, \end{aligned} \tag{84}$$

where $\gamma_d = \gamma_{\text{wt}} = c_{dn}K/2c_f$. Since the qualitative nature of the dynamics depends upon solutions to $d\bar{n}/d\tau = 0$, decay mismatch does not alter expression patterns qualitatively. However, the reporter concentration does depend quantitatively on the ratio c_{dr}/c_{dn}. In the same way, in PP and MPP reporters, if the reporter protein(s) have decay constants that are different to that of Nanog, then this will not change to α or γ, and so will not change the dynamics qualitatively. By contrast, in fusion reporters if the Nanog-reporter fusion has a different decay rate to that of the wild-type Nanog then there is the potential to cause a qualitative perturbation to the dynamics, as the value of c_{dn} and hence γ will be altered.

2 Supporting Tables and Figures

Supplementary Table 1

Reporter type	ODEs	Dimensionless variables	γ	Dimensionless ODEs	Reporter-Nanog relationship at eqbm.
BAC	$\frac{dn}{dt} = 2c_b + 2\frac{c_f n^H}{K^H + n^H} - c_d n$<br>$\frac{dr}{dt} = c_b + \frac{c_f n^H}{K^H + n^H} - c_d r$	$n = \frac{2c_f}{c_d}\bar{n}$<br>$r = \frac{c_f}{c_d}\bar{r}$	$\gamma_{\mathrm{b}} = \gamma_{\mathrm{wt}}$	$\frac{d\bar{n}}{d\tau} = \alpha + \frac{\bar{n}^H}{\gamma_{\mathrm{b}}^H + \bar{n}^H} - \bar{n}$<br>$\frac{d\bar{r}}{d\tau} = \alpha + \frac{\bar{n}^H}{\gamma_{\mathrm{b}}^H + \bar{n}^H} - \bar{r}$	$r = \frac{n}{2}$
Knock-in	$\frac{dn}{dt} = c_b + c_f\frac{n^H}{K^H + n^H} - c_d n$<br>$\frac{dr}{dt} = c_b + c_f\frac{n^H}{K^H + n^H} - c_d r$	$n = \frac{c_f}{c_d}\bar{n}$<br>$r = \frac{c_f}{c_d}\bar{r}$	$\gamma_{\mathrm{ki}} = 2\gamma_{\mathrm{wt}}$	$\frac{d\bar{n}}{d\tau} = \alpha + \frac{\bar{n}^H}{\gamma_{\mathrm{ki}}^H + \bar{n}^H} - \bar{n}$<br>$\frac{d\bar{r}}{d\tau} = \alpha + \frac{\bar{n}^H}{\gamma_{\mathrm{ki}}^H + \bar{n}^H} - \bar{r}$	$r = n$
Pre/post	$\frac{dn_1}{dt} = c_b + c_f\frac{n^H}{K^H + n^H} - c_d n_1$<br>$\frac{dn_2}{dt} = \epsilon c_b + \epsilon c_f\frac{n^H}{K^H + n^H} - c_d n_2$<br>$\frac{dr}{dt} = \epsilon c_b + \epsilon c_f\frac{n^H}{K^H + n^H} - c_d r$<br>$\frac{dn}{dt} = (1+\epsilon)\big(c_b + c_f\frac{n^H}{K^H + n^H}\big) - c_d n$	$n = \frac{c_f(1+\epsilon)}{c_d}\bar{n}$<br>$r = \frac{c_f\epsilon}{c_d}\bar{r}$	$\gamma_{\mathrm{pp}} = \frac{2\gamma_{\mathrm{wt}}}{1+\epsilon}$	$\frac{d\bar{n}}{d\tau} = \alpha + \frac{\bar{n}^H}{\gamma_{\mathrm{pp}}^H + \bar{n}^H} - \bar{n}$<br>$\frac{d\bar{r}}{d\tau} = \alpha + \frac{\bar{n}^H}{\gamma_{\mathrm{pp}}^H + \bar{n}^H} - \bar{r}$	$r = \frac{\epsilon n}{1+\epsilon}$
Fusion	$\frac{dn_1}{dt} = c_b + c_f\frac{n_{\mathrm{eff}}^H}{K^H + n_{\mathrm{eff}}^H} - c_d n_1$<br>$\frac{dn_2}{dt} = \epsilon c_b + \epsilon c_f\frac{n_{\mathrm{eff}}^H}{K^H + n_{\mathrm{eff}}^H} - c_d n_2$<br>$\frac{dn}{dt} = (1+\epsilon)\big(c_b + c_f\frac{n_{\mathrm{eff}}^H}{K^H + n_{\mathrm{eff}}^H}\big) - c_d n$<br>$\frac{dn_{\mathrm{eff}}}{dt} = (1+\epsilon\delta)\big(c_b + c_f\frac{n_{\mathrm{eff}}^H}{K^H + n_{\mathrm{eff}}^H}\big) - c_d n_{\mathrm{eff}}$	$n_1 = \frac{c_f(1+\epsilon\delta)}{c_d}\bar{n}_1$<br>$n_2 = \frac{c_f(1+\epsilon\delta)}{c_d}\bar{n}_2$<br>$n = \frac{c_f(1+\epsilon\delta)}{c_d}\bar{n}$<br>$n_{\mathrm{eff}} = \frac{c_f(1+\epsilon\delta)}{c_d}\bar{n}_{\mathrm{eff}}$	$\gamma_{\mathrm{fus}} = \frac{2\gamma_{\mathrm{wt}}}{1+\epsilon\delta}$	$\frac{d\bar{n}_1}{d\tau} = \frac{1}{1+\epsilon\delta}\big(\alpha + \frac{\bar{n}_{\mathrm{eff}}^H}{\gamma_{\mathrm{fus}}^H + \bar{n}_{\mathrm{eff}}^H}\big) - \bar{n}_1$<br>$\frac{d\bar{n}_2}{d\tau} = \frac{\epsilon}{1+\epsilon\delta}\big(\alpha + \frac{\bar{n}_{\mathrm{eff}}^H}{\gamma_{\mathrm{fus}}^H + \bar{n}_{\mathrm{eff}}^H}\big) - \bar{n}_2$<br>$\frac{d\bar{n}}{d\tau} = \frac{1+\epsilon}{1+\epsilon\delta}\big(\alpha + \frac{\bar{n}_{\mathrm{eff}}^H}{\gamma_{\mathrm{fus}}^H + \bar{n}_{\mathrm{eff}}^H}\big) - \bar{n}$<br>$\frac{d\bar{n}_{\mathrm{eff}}}{d\tau} = \alpha + \frac{\bar{n}_{\mathrm{eff}}^H}{\gamma_{\mathrm{fus}}^H + \bar{n}_{\mathrm{eff}}^H} - \bar{n}_{\mathrm{eff}}$	$r = n_2 = \frac{\epsilon n}{1+\epsilon}$

Supporting Table 1: **Perturbations by single allele reporters.** Summary of analysis of reporter perturbations to wild-type dynamics for single allele reporters.

Supplementary Table 2

Reporter type	ODEs	Dimensionless variables	γ		Dimensionless ODEs	Reporter-Nanog relation at eqbm.	
			Same reporters	Different reporters		Same reporters	Different reporters
Pre/post	$\frac{dn_1}{dt} = \epsilon_1 c_b + \epsilon_1 c_f \frac{n^H}{K^H + n^H} - c_d n_1$<br>$\frac{dn_2}{dt} = \epsilon_2 c_b + \epsilon_2 c_f \frac{n^H}{K^H + n^H} - c_d n_2$<br>$\frac{dr_1}{dt} = \epsilon_1 c_b + \epsilon_1 c_f \frac{n^H}{K^H + n^H} - c_d r_1$<br>$\frac{dr_2}{dt} = \epsilon_2 c_b + \epsilon_2 c_f \frac{n^H}{K^H + n^H} - c_d r_2$<br>$\frac{dn}{dt} = (\epsilon_1 + \epsilon_2)\big(c_b + c_f \frac{n^H}{K^H + n^H}\big) - c_d n$	$n = \frac{c_f(\epsilon_1 + \epsilon_2)}{c_d}\bar{n}$<br>$r_1 = \frac{c_f \epsilon_1}{c_d}\bar{r}_1$<br>$r_2 = \frac{c_f \epsilon_2}{c_d}\bar{r}_2$	$\frac{\gamma_{\mathrm{wt}}}{\epsilon}$	$\frac{2\gamma_{\mathrm{wt}}}{\epsilon_1 + \epsilon_2}$	$\frac{d\bar{n}}{d\tau} = \alpha + \frac{\bar{n}^H}{\gamma_{\mathrm{pp2}}^H + \bar{n}^H} - \bar{n}$<br>$\frac{d\bar{r}_1}{d\tau} = \alpha + \frac{\bar{n}^H}{\gamma_{\mathrm{pp2}}^H + \bar{n}^H} - \bar{r}_1$<br>$\frac{d\bar{r}_2}{d\tau} = \alpha + \frac{\bar{n}^H}{\gamma_{\mathrm{pp2}}^H + \bar{n}^H} - \bar{r}_2$	$r = n$	$r = r_1 + r_2 = n$<br>$r_1 = \frac{\epsilon_1 n}{\epsilon_1 + \epsilon_2}$<br>$r_2 = \frac{\epsilon_2 n}{\epsilon_1 + \epsilon_2}$
Fusion	$\frac{dn_1}{dt} = \epsilon_1 c_b + \epsilon_1 c_f \frac{n_{\mathrm{eff}}^H}{K^H + n_{\mathrm{eff}}^H} - c_d n_1$<br>$\frac{dn_2}{dt} = \epsilon_2 c_b + \epsilon_2 c_f \frac{n_{\mathrm{eff}}^H}{K^H + n_{\mathrm{eff}}^H} - c_d n_2$<br>$\frac{dn}{dt} = (\epsilon_1 + \epsilon_2)\big(c_b + c_f \frac{n_{\mathrm{eff}}^H}{K^H + n_{\mathrm{eff}}^H}\big) - c_d n$<br>$\frac{dn_{\mathrm{eff}}}{dt} = (\epsilon_1\delta_1 + \epsilon_1\delta_2)\big(c_b + c_f \frac{n_{\mathrm{eff}}^H}{K^H + n_{\mathrm{eff}}^H}\big) - c_d n_{\mathrm{eff}}$	$n_1 = \frac{c_f(\epsilon_1\delta_1 + \epsilon_2\delta_2)}{c_d}\bar{n}_1$<br>$n_2 = \frac{c_f(\epsilon_1\delta_1 + \epsilon_2\delta_2)}{c_d}\bar{n}_2$<br>$n = \frac{c_f(\epsilon_1\delta_1 + \epsilon_2\delta_2)}{c_d}\bar{n}$<br>$n_{\mathrm{eff}} = \frac{c_f(\epsilon_1\delta_1 + \epsilon_2\delta_2)}{c_d}\bar{n}_{\mathrm{eff}}$	$\frac{\gamma_{\mathrm{wt}}}{\epsilon\delta}$	$\frac{2\gamma_{\mathrm{wt}}}{\epsilon_1\delta_1 + \epsilon_2\delta_2}$	$\frac{d\bar{n}_1}{d\tau} = \frac{\epsilon_1}{\epsilon_1\delta_1 + \epsilon_2\delta_2}\big(\alpha + \frac{\bar{n}_{\mathrm{eff}}^H}{\gamma_{\mathrm{fus2}}^H + \bar{n}_{\mathrm{eff}}^H}\big) - \bar{n}_1$<br>$\frac{d\bar{n}_2}{d\tau} = \frac{\epsilon_2}{\epsilon_1\delta_1 + \epsilon_2\delta_2}\big(\alpha + \frac{\bar{n}_{\mathrm{eff}}^H}{\gamma_{\mathrm{fus2}}^H + \bar{n}_{\mathrm{eff}}^H}\big) - \bar{n}_2$<br>$\frac{d\bar{n}}{d\tau} = \frac{\epsilon_1 + \epsilon_2}{\epsilon_1\delta_1 + \epsilon_2\delta_2}\big(\alpha + \frac{\bar{n}_{\mathrm{eff}}^H}{\gamma_{\mathrm{fus2}}^H + \bar{n}_{\mathrm{eff}}^H}\big) - \bar{n}$<br>$\frac{d\bar{n}_{\mathrm{eff}}}{d\tau} = \alpha + \frac{\bar{n}_{\mathrm{eff}}^H}{\gamma_{\mathrm{fus2}}^H + \bar{n}_{\mathrm{eff}}^H} - \bar{n}_{\mathrm{eff}}$	$r = n$	$r = n_1 + n_2 = n$<br>$n_1 = \frac{\epsilon_1 n}{\epsilon_1 + \epsilon_2}$<br>$n_2 = \frac{\epsilon_2 n}{\epsilon_1 + \epsilon_2}$

Supporting Table 2: **Perturbations by dual allele reporters.** Summary of analysis of reporter perturbations to wild-type dynamics for dual allele reporters.

Figure S1

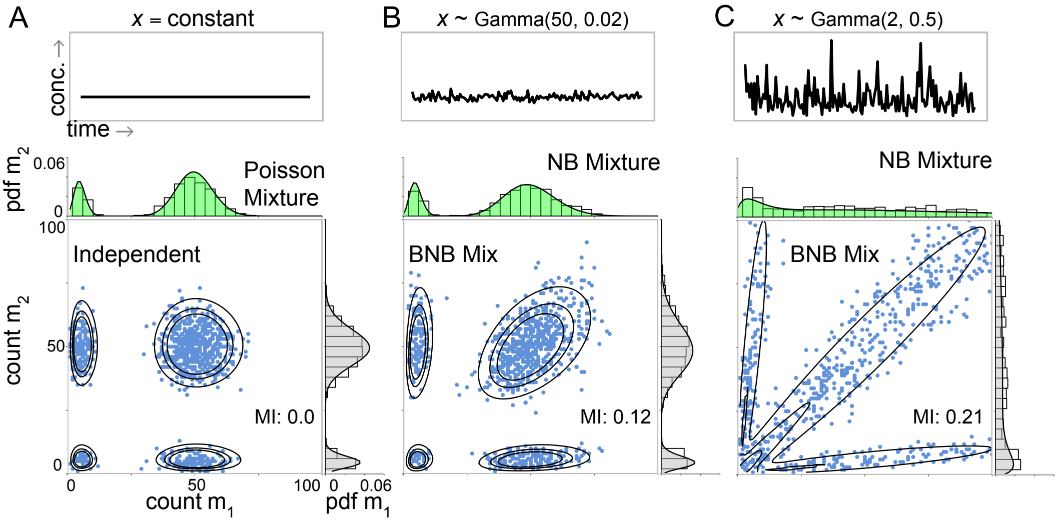

Supporting Figure 1: **Reporter accuracy depends upon regulatory context: 2-state genes.** Identical alleles of the same gene produce mRNA molecules M_1 and M_2. **Top panels**: Fluctuations of upstream regulator concentration, x. Panels show constant x (**A**) and $x \sim \text{Gamma}(r, \theta)$, for low regulator dispersion $\theta = 0.02$ (**B**) and high regulator dispersion $\theta = 0.5$ (**C**). **Bottom panels**: Joint and marginal distributions of m_1 and m_2 with upstream regulation given in top panel for two-state genes: Joint distribution given by the product of two Poisson mixtures (Eqn. (35)) or by bivariate BNB mixtures (Eqn. (40)), with $w = 0.8, \lambda_+ = 50$ and $\lambda_- = 5$ in all cases. w is the probability the gene is in the active state $(w_+/(w_+ + w_-))$ and λ_+, λ_- are the effective production rates in the 2 states. Marginal distributions are Poisson mixtures and negative binomial mixtures. For all joint distributions, contours show probabilities: 0.0001 inner, 0.0003 middle, 0.0005 outer. Scatter plots, histograms and mutual information (nats) are shown for a random sample of 1000 draws. The same scales apply to all comparable plots.

Figure S2

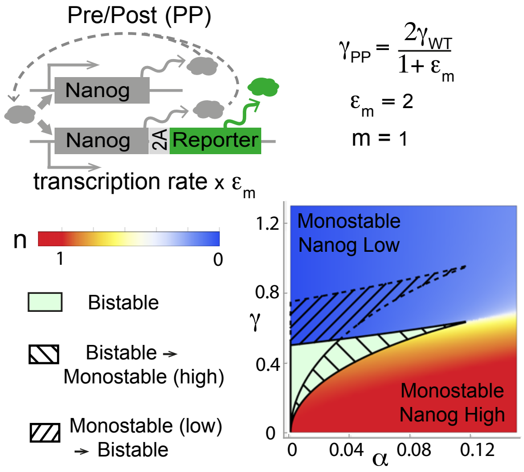

Supporting Figure 2: **Pre/Post (PP) Reporter with increased transcription rate.** The Pre/Post reporters can result in a change of transcription rate and this can either be a decrease (as shown in Figure 2) or an increase as shown in this case. When transcription rate increases by a factor of 2 ($\epsilon_m = 2$), γ_{pp} is reduced compared to γ_{wt}. Hatched areas indicate at risk regions of the parameter plane.

Figure S3

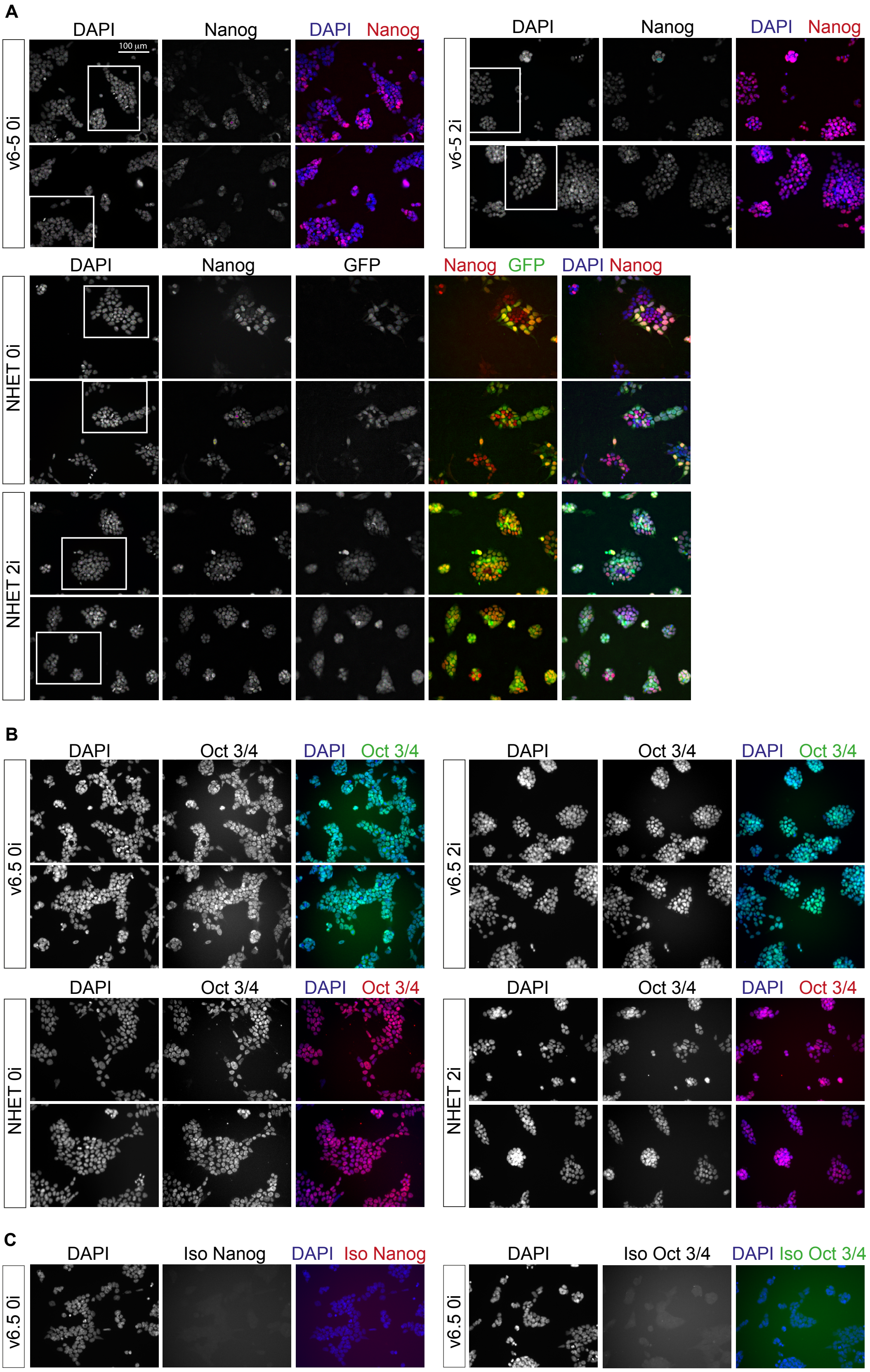

Supporting Figure 3: **Nanog and Oct3/4 immunofluorescence. A** Grayscale and composite RGB images of DAPI staining, Nanog immunofluorescence and GFP fluorescence in v6.5 and NHET cells in 0i and 2i conditions. Boxes indicate the regions of the image shown in Figure 1 main text. Variability of Nanog fluorescence can be seen for both v6.5 and NHET cells, with substantially greater variability in 0i conditions. **B** Grayscale and composite RGB images for Oct3/4 immunofluorescence from v6.5 and NHET cells in 0i and 2i cultures. Oct3/4 is less variably expressed than Nanog. **C** Grayscale and composite RGB images for Nanog and Oct3/4 antibody isotype controls for v6.5 0i cells.

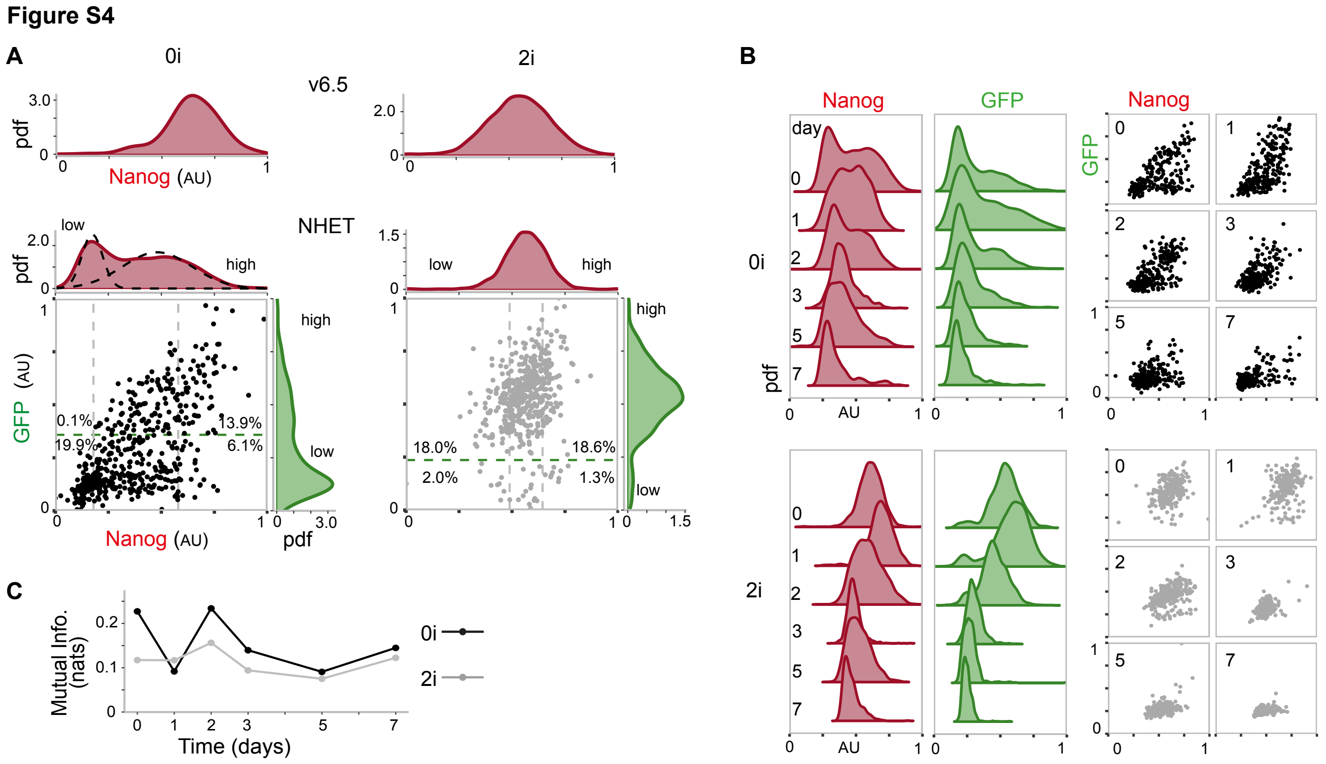

Supporting Figure 4: **Image analysis of Nanog variability in v6.5 and NHET cells. A** Examples of Nanog immunofluorescence distributions for v6.5 cells and joint Nanog-GFP distributions for NHET cells in 0i and 2i conditions, assessed by image analysis. NHET populations are split by GFP expression (high/low) and Nanog expression (highest 20% /lowest 20%). Percentages are shown for outer subpopulations. **B**. Assessment of joint Nanog-GFP distributions in NHET cells during differentiation subsequent to LIF-withdrawal starting from 0i and 2i cultures, using data assessed by image analysis. **C**. Mutual information between Nanog and GFP during differentiation, using data assessed by image analysis.
